# A biocompatible Lossen rearrangement in *Escherichia coli*

**DOI:** 10.1038/s41557-025-01845-5

**Published:** 2025-06-23

**Authors:** Nick W. Johnson, Marcos Valenzuela-Ortega, Thomas W. Thorpe, Yuta Era, Annemette Kjeldsen, Keith Mulholland, Stephen Wallace

**Affiliations:** 1 Institute of Quantitative Biology, Biochemistry and Biotechnology, School of Biological Sciences, University of Edinburgh, Edinburgh, UK; 2 Chemical Development, Pharmaceutical Technology and Development, Operations, AstraZeneca, Macclesfield, UK

**Keywords:** Biosynthesis, Biocatalysis, Metabolic engineering, Synthetic biology

## Abstract

Nature has evolved an exquisite yet limited set of chemical reactions that underpin the function of all living organisms. By contrast, the field of synthetic organic chemistry can access reactivity not observed in nature, and integration of these abiotic reactions within living systems offers an elegant solution to the sustainable synthesis of many industrial chemicals from renewable feedstocks. Here we report a biocompatible Lossen rearrangement that is catalysed by phosphate in the bacterium *Escherichia coli* for the transformation of activated acyl hydroxamates to primary amine-containing metabolites in living cells. Through auxotroph rescue, we demonstrate how this new-to-nature reaction can be used to control microbial growth and chemistry by generating the essential metabolite *para*-aminobenzoic acid. The Lossen rearrangement substrate can also be synthesized from polyethylene terephthalate and applied to whole-cell biocatalytic reactions and fermentations generating industrial small molecules (including the drug paracetamol), paving the way for a general strategy to bioremediate and upcycle plastic waste in native and engineered biological systems.

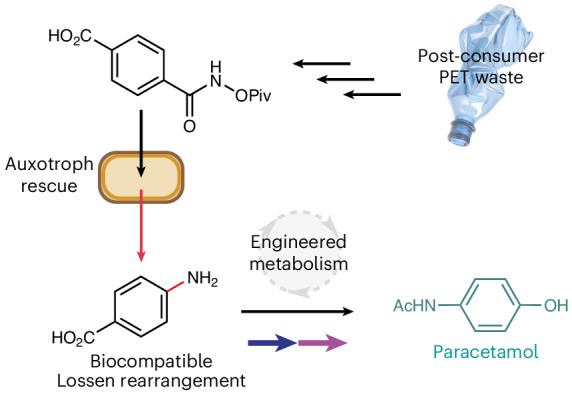

## Abstract (web summary)

Biocompatible chemistry merges chemo-catalytic reactions with cellular metabolism for sustainable small-molecule synthesis. Now a biocompatible Lossen rearrangement has been demonstrated to control bacterial cell growth and chemistry and applied to the remediation and upcycling of polyethylene terephthalate plastic waste in whole-cell reactions and fermentations to produce valuable industrial chemicals, including the drug paracetamol.

## Main

The development of biocompatible reactions—non-enzymatic chemical transformations that can be interfaced with cellular metabolism—is a nascent approach to expanding the synthetic repertoire of living systems^1–5^. Using synthetic strategies established in modern organic chemistry, biocompatible reactions can be applied to the control of cellular function^6–9^, the diversification of metabolites in vivo^10–12^ and biological access to otherwise recalcitrant feedstocks for industrial biotechnology^13–15^. This approach complements existing methods for abiotic catalysis in cells^16–18^, including directed evolution^19,20^, the creation of non-native active sites using artificial cofactors or unnatural amino acids^21^, and the bottom-up design of enzymes using computational methods^22^. However, the reconstitution of new-to-nature biocatalysts in vivo is challenging and has often limited their application to in vitro reactions. The metabolic integration of non-native chemistries in living cells and particularly within the context of cellular metabolism remains a great challenge in the field of chemical biotechnology^2^.

Strategies to increase the limited toolbox of metabolic chemistry for microbial synthesis would enable the bioproduction of an increased range of industrial small molecules from sustainable feedstocks using engineered biology, lowering existing chemical manufacturing routes reliance on diminishing fossil fuels. Recent work in this area is limited to the use of artificial metalloenzymes in microbial cells that have been metabolically engineered to generate substrate(s) and apoenzyme, followed by transport of a non-native cofactor to the cell interior for biosynthesis^23^. To this end, Huang et al. reported the non-natural cyclopropanation of metabolically derived limonene in engineered *Escherichia coli* using an intracellular Ir-CYP119 metalloenzyme and exogenous ethyl diazoacetate^24^. Import of the Ir-porphyrin cofactor for biosynthesis was enabled by co-expressing the *hug* operon haem transport system from *Plesiomonas shigelloides* alongside a heterologous limonene biosynthetic pathway and together enabled the bioproduction of an unnatural cyclopropane-containing terpenoid in 0.25 mg l^−1^ and a 1:7.0:2.3:1.5 ratio of diastereoisomers. Biocompatible cyclopropanation chemistry has also been demonstrated at the outer membrane of *E. coli* and in membrane-associated micelles using a non-enzymatic Fe-phthalocyanine catalyst to intercept styrene generated in vivo from d-glucose via engineered metabolism (95% yield, 553 mg l^−1^)^25^. More recently, however, Huang et al. have achieved the complete integration of non-native carbene-transfer-based cyclopropanation chemistry into microbial metabolism in the bacterium *Streptomyces albus* J1074^26^. Here, the authors combine a heterologous styrene biosynthesis pathway from L-phenylalanine with the native biosynthesis of the diazo-containing natural product azaserine and an Ir(Me)MPIX artificial metalloenzyme to enable the bioproduction of an unnatural cyclopropane-containing metabolite from entirely biobased building blocks in vivo (0.22 mg l^−1^). In addition to combining the end products of two metabolic pathways using an artificial enzyme, in a different report, Liu et al. used a hemin-catalysed oxidative decarboxylation reaction to convert metabolically generated α-acetolactate to diacetyl and subsequently (*S*,*S*)-2,3-butanediol in engineered *Lactococcus lactis*^27^. Most recently, Dennis et al. demonstrated the biocompatible organocatalytic α-methylenation of metabolically generated butyraldehyde, which was then subsequently reduced to 2-methylbutanal in engineered *E. coli*^28^. Together, these studies demonstrate the metabolic flexibility of microorganisms and how chemical principles can be used within metabolic pathways using abiotic catalysis to generate new-to-nature small molecules in cells.

**Fig. 1:**
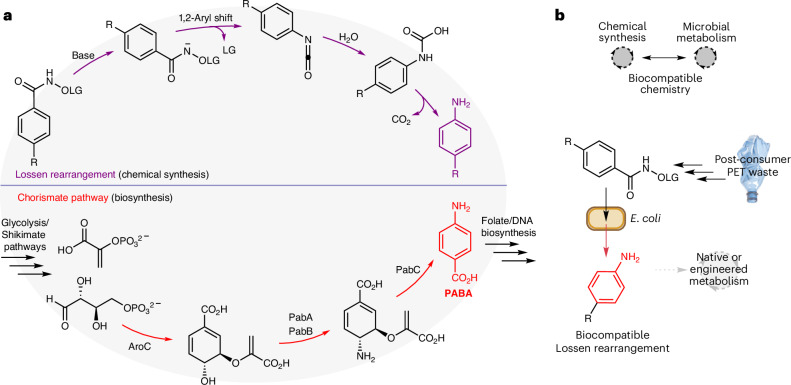
Aniline synthesis from carboxylic acids in vitro and in vivo*.* **a**, A comparison of strategies for C–N bond formation via Lossen rearrangement in synthetic organic chemistry or via chorismate pathways in cellular metabolism. **b**, The proposed merging of non-enzymatic Lossen rearrangement chemistry with cellular metabolism for sustainable synthesis and the bio-upcycling of plastic waste. LG, leaving group.

An unexplored area of biocompatible chemistry is the non-enzymatic rearrangement of activated carboxylate substrates and their integration with native and engineered metabolic pathways in cells. Discovered in 1872 by Wilhelm Lossen, the Lossen rearrangement is characterized by the thermal- or metal-catalysed expulsion of a carboxylate from a *bis*-acylated hydroxylamine substrate^29^ (Fig. 1a). The rearrangement typically involves a phenyl hydroxamate ester and proceeds under basic conditions via 1,2-aryl migration to form an isocyanate that rapidly reacts with water under aqueous conditions to form a carbamic acid, followed by decarboxylation to the primary amine product^30,31^. The reaction is synthetically useful as the Lossen rearrangement substrate can be formed from readily available carboxylic acids, avoids the use of azide reagents (cf. the Curtius rearrangement) and occurs under mild conditions^32,33^. Overall, the reaction generates primary amines from carboxylate substrates with an accompanying one-carbon contraction, contrasting with the enzymatic chemistry enabled by ammonia lyases and aminotransferases^34–36^. Lossen-type rearrangements have been observed in vitro as unproductive substrates^37^ using chymotrypsin and are found as intermediates responsible for hydroxamic acid toxicity in *Salmonella typhimurium* TA98^38^. However, to the best of our knowledge, the Lossen rearrangement has never been interfaced with microbial metabolism for biocompatible chemistry and therefore remains a functional group transformation that is unique to the field of synthetic organic chemistry.

Here, we report the biocompatible Lossen rearrangement of acyl hydroxamates in living cells and interface this abiotic reaction with cellular metabolism for native and de novo biosynthesis in *E. coli*. The reaction occurs in vivo, under ambient conditions, is non-toxic to *E. coli* and is catalysed by phosphate in cells. We go on to synthesize the Lossen rearrangement substrate from polyethylene terephthalate (PET) and show through auxotroph rescue experiments how *E. coli* growth and metabolism can be dependent upon a plastic-bottle-derived small molecule (Fig. 1b). Finally, metabolic cooperation with the Lossen rearrangement is demonstrated through the conditional biotransformation of exogenous alkenes and of PET-derived *para*-aminobenzoate (PABA) to the analgesic and antipyretic drug paracetamol (*para*-hydroxyacetanilide). Overall, this work expands the available toolbox of metabolic chemistry for small-molecule synthesis in native and engineered cells.

## Results and discussion

### Reaction screening via auxotroph rescue

**Fig. 2:**
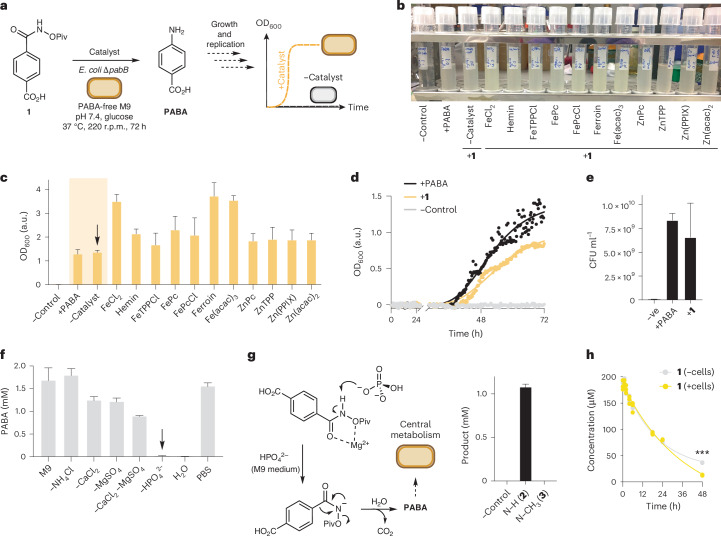
Reaction discovery by auxotroph rescue. **a**, Auxotroph rescue experiment via Lossen rearrangement. **b**, A photograph of cultures after the auxotroph recovery of *E. coli* BW25113*∆pabB* in the presence of **1** and various transition-metal catalysts. **c**, Culture density measurements by spectrophotometry. **d**, Auxotroph rescue growth curves from cultures of *E. coli* BW25113*∆pabB* grown in the presence of PABA or **1**. The extended lag phase is due to the 10^5^-fold dilution of the starting inoculum that is required to ensure no background growth without PABA. **e**, Plate-count assay from cultures of *E. coli* BW25113*∆pabB* grown in the presence of PABA or **1**. **f**, In vitro experiment examining the effect of M9 media components on the Lossen rearrangement of **1**. **g**, The proposed phosphate-catalysed Lossen rearrangement of **1** to PABA and in vitro reactivity of *O*-acylated and *N*-methylated control compounds **2** and **3**. **h**, Substrate depletion assay using 200 µM **1** in the presence and absence of metabolically active cells. ***P < 0.0005 (*t*-test). All data are presented as mean values ± s.d. of three biological replicates. Source data

Inspired by Li et al. on the Fe-phenanthroline-catalysed Lossen rearrangement of heteroauxin-derived substrates in dichloromethane at room temperature and the reported hemin-catalysed N–O insertion chemistry in artificial enzymes^39–42^, we reasoned that a biocompatible metal catalyst would be effective in generating an intermediate nitrenoid from an *O*-pivaloyl (*O*-Piv)-substituted benzhydroxamate substrate under aqueous conditions^39^. We therefore set out to investigate whether the Lossen rearrangement was biocompatible and whether it could be interfaced with microbial metabolism to enable non-natural biosynthesis. To test catalyst reactivity and biocompatibility simultaneously, we designed an auxotroph rescue experiment where a *para*-carboxyl *O*-pivalolylhydroxamate Lossen rearrangement substrate **1** would generate the essential metabolite PABA in situ. PABA is essential for folic acid biosynthesis in bacteria, and organisms deficient in PABA cannot grow due to defects in nucleotide and DNA metabolism^6,43^. Auxotrophic organisms (including humans) must therefore sequester these essential nutrients from their surrounding environment or from other (micro)organisms living in close proximity. Therefore, a successful Lossen rearrangement of **1** would generate PABA and result in growth of the auxotrophic cells that could be detected by optical density (OD_600_) measurement (Fig. 2a). Moreover, toxic catalysts would inhibit cell growth irrespective of PABA generation and, therefore, successful growth would be a positive indicator of both catalyst activity and biocompatibility. To this end, *O*-Piv benzhydroxamate **1** was synthesized in two steps from 4-formylbenzoic acid via amide bond formation with *O*-Piv hydroxylamine followed by aldehyde oxidation using periodic acid and catalytic pyridinium chlorochromate (Supplementary Scheme 1). We selected a series of K-12-derived Keio collection knockout strains including *E. coli* BW25113∆*pabB* deficient in the catalytic subunit of aminodeoxychorismate synthase that is involved in the penultimate step of PABA biosynthesis (Fig. 1a and Supplementary Fig. 1). The catalyst screen was populated with metal complexes that have been reported in the literature to promote the Lossen or Curtius rearrangement and/or analogous reactions under mild or aqueous reaction conditions^39,44,45^. These include FeCl_2_, hemin, iron phthalocyanine, ferroin, ZnTPP, Zn(PPIX) and Zn(acac)_2_. Lossen substrate **1** (10 µM) was added to growth tubes containing M9-glycerol medium and catalyst (10 mol%) followed by *E. coli* BW25113∆*pabB* inoculated from a saturated starter culture grown in M9-glycerol medium containing PABA (10^5^ dilution). Cultures were then incubated for 72 h at 37 °C at 220 r.p.m. (Fig. 2a). As expected, no cell growth was observed in the absence of PABA. However, we were surprised to observe that cell growth occurred in every other tube (Fig. 2b,c). Crucially, growth was observed in control cultures containing **1** and no catalyst, indicating that the Lossen rearrangement of **1** was biocompatible and either occurring spontaneously in growth media, being promoted by cellular components (for example, membranes) or being catalysed by a native enzyme in *E. coli* (Fig. 2d,e). To eliminate the latter two, PABA was quantified using a *N*-(1-napthyl)ethylenediamine colorimetric assay in control reactions performed in M9-glycerol only (Fig. 2f and Supplementary Fig. 2). Indeed, PABA was detected in the absence of cells and was not detected when **1** was incubated in ultrapure H_2_O. Sequential elimination of each component of M9 medium (NH_4_Cl, CaCl_2_, MgSO_4_ and HPO_4_^2−^) and comparison with PABA generation in phosphate-buffered saline (PBS) revealed that the Lossen rearrangement of **1** was mediated by phosphate. Aniline formation from *N*-methyl *O*-acetyl benzhydroxamic acid **3** or benzhydroxamic acid **4** was also abolished under these conditions (Fig. 2g and Supplementary Fig. 3), demonstrating that the reaction probably proceeds via initial N–H deprotonation followed by 1,2-aryl migration, rather than ester hydrolysis followed by a phosphate-catalysed rearrangement of the corresponding hydroxamic acid (Fig. 2g). Spectrophotometric analysis of the cultures revealed that the highest OD_600_ was observed in cultures incubated in the presence of **1** and either FeCl_2_, ferroin or Fe(acac)_3_ (Fig. 2c). Intriguingly, these complexes are the weakest Fe binders, and therefore the higher growth of these cultures is probably due to a non-enzymatic phosphate-catalysed Lossen rearrangement followed by increased Fe availability to the microorganism during the log-phase of growth, which is limiting in the tested conditions. Auxotroph rescue was also confirmed in Keio knockout strains *E. coli* BW25113*∆pabA* and *E. coli* BW25113*∆aroC*, and both displayed either an extended lag phase or lower final cell density when compared with *E. coli* BW25113*∆pabB* grown in the presence of PABA or **1** (Supplementary Fig. 1). The toxicity of **1** to *E. coli* BW25113*∆pabB* was determined by a serial dilution and plate count assay. All substrates were biocompatible with *E. coli* growth by OD_600_ and colony-forming units (CFU) per millilitre in the 10–1,000 µM range (Fig. 2e and Supplementary Fig. 4). A panel of *O*-acyl substrates containing hydrophilic and hydrophobic groups were screened and converted to aromatic amines with no notable rate increase by colorimetric assay, except for a rapid product formation when using pentafluorobenzyl substrate **S2** (Supplementary Fig. 3) and abolished Lossen reactivity using the hydrophilic *O*-succinyl substrate **S6** (Supplementary Fig. 3). Competing hydrolysis to benzhydroxamic acid **4** was observed for the *O*-Ac substrate and so **1** was selected for further study. Finally, high-performance liquid chromatography (HPLC) quantification over time in the presence and absence of replicating *E. coli* BW25113∆*pabB* cells revealed increased substrate consumption after >24 h at the point where cells enter log-phase growth, indicating that the Lossen rearrangement of **1** is not only biocompatible but also potentially accelerated in the presence of metabolically active cells (Fig. 2h).

### PET bioremediation

**Fig. 3:**
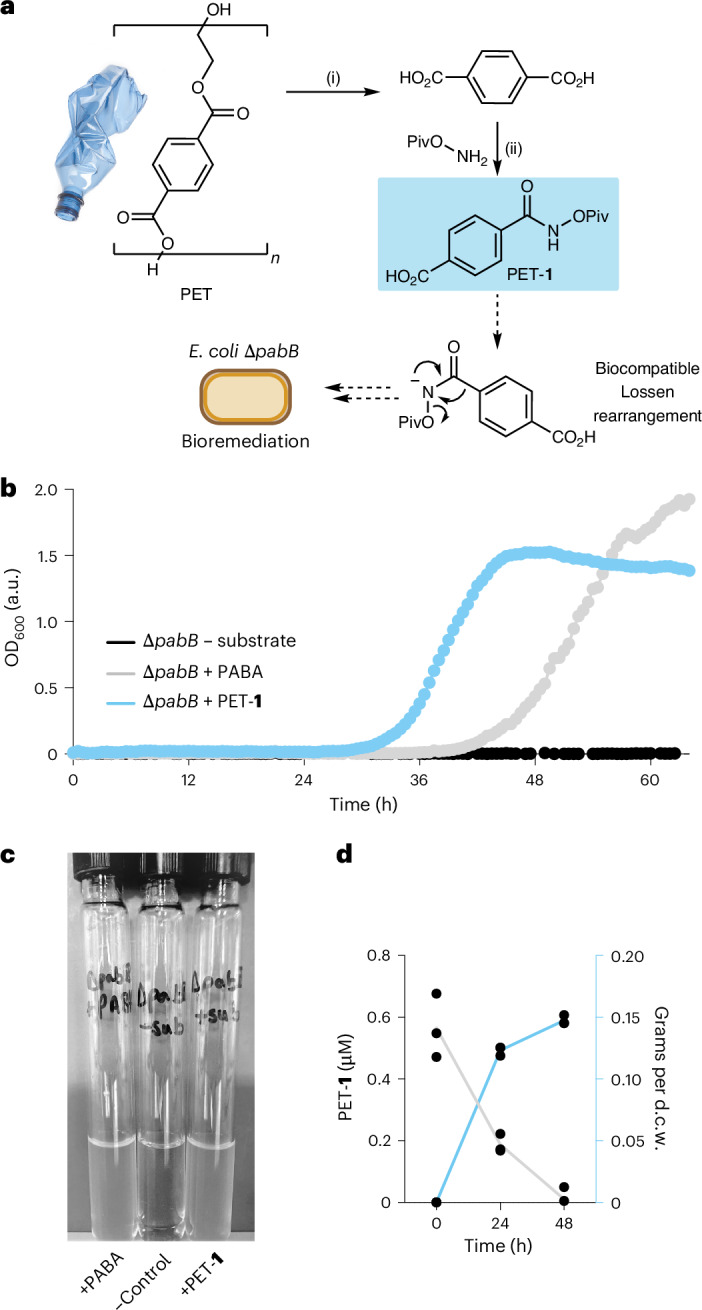
Substrate synthesis from PET plastic waste for bioremediation. **a**, Synthesis of the Lossen rearrangement substrate from PET waste and PET bioremediation strategy via auxotroph recovery. **b**, Growth curves from 96-well plate experiments during auxotroph rescue. Error bars are omitted for clarity. **c**, A photograph of auxotroph rescue experiments using a PET-derived substrate. **d**, Substrate depletion (grey) and dry cell weight (d.c.w.) production (blue) during growth experiments in Falcon tubes using 1 µM PET-**1**. A conversion factor of 0.33 grams per litre per OD_600_ was applied. All data are shown as triplicate experiments to one standard deviation. (i) NaOH, EtOH/H_2_O, reflux, 10 h. (ii) T3P, iPr_2_NEt, H_2_NOPiv·TfOH, tetrahydrofuran, 0 °C to room temperature, 16 h. Source data

Despite our initial focus on developing biocompatible reactions for synthesis in microbial cells, this intriguing observation suggested a method for the bioremediation of **1** derived from waste materials. For example, the synthesis of **1** can be envisioned from terephthalic acid—the depolymerization monomer of PET plastic waste. We therefore reasoned that we could synthesize **1** from PET and by introducing PABA auxotrophy into a microbial strain effectively condition cell growth to the presence of PET-derived small molecules, offering a strategy for the remediation of this prolific waste material and environmental pollutant into microbial biomass. Industrial PET production worldwide is currently 56 million tons per year, and approximately 80% of this is designed to be single-use, leading to around 24 million tons of PET waste each year that is either incinerated or sent to landfill^46^. To this end, a synthesis of **1** was optimized from a discarded plastic bottle in two steps via the hydrolysis of PET flakes to terephthalic acid, followed by amide coupling with *O*-Piv hydroxylammonium triflate and propylphosphonic anhydride (T3P) to generate PET-**1** (Fig. 3a and Supplementary Scheme 1). Auxotroph recovery and growth of *E. coli* BW25113∆*pabB* was observed using this PET-derived substrate, accompanied by quantitative reduction in substrate concentration and no detectable PABA after 48 h (Fig. 3b–d). Growth in the presence of PET-**1** had a comparable rate (0.25 h^−^^1^ for PABA and 0.33 h^−^^1^ for PET-**1** after 40 h), final cell density (OD_600_) and viability count (CFU ml^−1^) at stationary phase and led to a similar decrease in substrate compared with using **1** (Fig. 3d and Supplementary Fig. 5).

### Interfaced metabolic synthesis

**Fig. 4:**
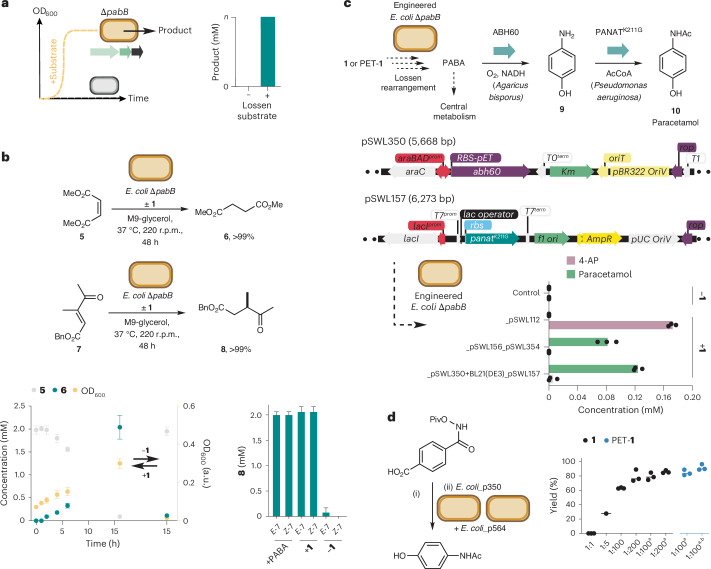
Interfacing the Lossen rearrangement with native and engineered metabolic pathways. **a**, Interfacing the Lossen rearrangement with native and engineered biosynthetic pathways in *E. coli*. **b**, Biotransformation of DMM (**5**) to dimethylsuccinate (**6**) and β-ketoacrylate **7** to γ-ketoester **8** using *E. coli* BW25113*∆pabB* and **1** or PET-**1**. **c**, A de novo biosynthetic pathway to 4-AP (**9**) and paracetamol **10** incorporating a non-enzymatic Lossen rearrangement, plasmid designs and whole-cell production experiments. **d**, Paracetamol synthesis by one-pot Lossen rearrangement and bacterial whole-cell synthesis. Strain *E. coli*_p350 expresses ABH60 and strain *E. coli*_p354 expresses PANAT^K211G^. Ratios refer to *E. coli*_p354 and *E. coli*_p350 in biotransformations (1:1, OD_600_ 26; 1:5, OD_600_ 16; 1:100 and 1:200, OD_600_ 12.5). ^a^Final cell density OD_600_ 25. OD refers to the final optical density at 600 nm. ^b^Cells induced with 0.5% l-arabinose. Reaction conditions: (i) substrate (0.5 mM), aqueous potassium phosphate (200 mM, pH 8.0), 50 °C, 48 h; (ii) *E. coli*_p354 and *E. coli*_p350 (1:10; OD_600_ 20). Biotransformations were analysed by ^1^H NMR relative to an internal standard of trimethoxybenzene or by HPLC relative to an internal standard of caffeine. All data are presented as mean values ± s.d. of three biological replicates. Source data

Having confirmed that the Lossen rearrangement was biocompatible and could be used to bioremediate PET-derived substrates, we next moved on to examine whether rescued cells could be used for biocatalytic reactions (Fig. 4a). We decided to focus this study initially on the whole-cell C=C bond reduction of maleates and keto-acrylates reported by Brewster et al. under fermentation conditions using native reductases in *E. coli*^47^. The substrates dimethyl maleate (DMM, **5**) and keto-acrylates (*E*)**-7** and (*Z*)-**7** were synthesized and added to cultures of *E. coli* BW25113∆*pabB* grown in the presence of **1**. Pleasingly, all the substrates were quantitatively reduced to dimethyl succinate **6** or γ-ketoester **8** by ^1^H nuclear magnetic resonance (NMR) after 24 h at 37 °C and product formation was observed only in the presence of PABA or **1**, indicating that the biocompatible Lossen rearrangement could be used to control the chemical output of a microbial fermentation (Fig. 4b and Supplementary Figs. 6 and 7). Having interfaced the Lossen rearrangement with cell growth, metabolism and native biosynthetic reactions, we moved on to assess whether the Lossen rearrangement product could be syphoned into a de novo metabolic pathway in vivo. To test this, we decided to target the biosynthesis of paracetamol (*para*-hydroxyacetanilide) **10** from **1** in engineered *E. coli*. Both **1** and **10** are non-toxic to *E. coli* BW25113∆*pabB* at concentrations up to 1 mM (265 mg l^−1^; **1**) and 6.6 mM (1 g l^−1^; **10**) (Supplementary Fig. 8). Paracetamol is the first-in-line World Health Organization-recommended treatment for pain and fever worldwide^48^. It is currently manufactured from phenol (derived from fossil fuels by the cumeme process) via nitration, reduction and *N*-acetylation with acetic anhydride before being formulated into an orally available medication^49^. By contrast, **10** can be synthesized in cells from PABA via 4-aminophenol (4-AP, **9**) by two enzymes: (1) an O_2_- and NADH-dependent aminobenzoate hydroxylase (ABH60) from the fungus *Agaricus bisporus* and (2) a K211G mutant of the acetyl CoA-dependent arylamine *N*-acyltransferase from the bacterium *Pseudomonas aeruginosa* (PANAT)^50^ (Fig. 4c). Paracetamol biosynthesis has been reported from d-glucose in *E. coli* but not from a waste feedstock or a PET-derived substrate. To this end, the genes encoding ABH60 and PANAT were synthesized and cloned into a Joint Universal Modular Plasmid (JUMP) vector generating plasmid pSWL112 (Supplementary Fig. 9 and Supplementary Table 3). The kanamycin resistance cassette from *E. coli* BW25113∆*pabB* Keio strain was removed using a pCP20-encoded flippase before transformation with pSWL112, generating an engineered and auxotrophic strain to produce paracetamol that could be recovered using **1** (Supplementary Fig. 10a). However, HPLC analysis of cultures of *E. coli* BW25113∆*pabB*_pSWL112 grown in the presence of **1** identified 4-acetamidobenzoic acid (4-AB, 31 mg l^−1^) as the sole product from the paracetamol pathway, resulting from unproductive *N*-acetylation of PABA by PANAT and acetyl-CoA (Fig. 4c and Supplementary Fig. 10b). To overcome this, a series of modified JUMP plasmids were designed containing *abh60* and *panat* genes under the control of constitutive and inducible promoters to optimize flux from PABA to paracetamol. Two optimization strategies were trialled: (1) growth of *E. coli* BW25113∆*pabB*_pSWL156_ pSWL354 or *E. coli* BW25113∆*pabB*_pSWL156_ pSWL355 in the presence of **1** with constitutive expression of *abh60* (pSWL156, J23100-*abh60*) and *panat* expression in plasmids pSWL354 (/P^bad^-*panat*) and pSWL355 (/P^tet^-*panat*) induced after 72 h (Supplementary Figs. 10b and 12); or (2) the separation of ABH60- and PANAT-catalysed reactions using 2 *E. coli* strains (BW25113∆*pabB*_pSWL156 or BW25113∆*pabB*_pSWL350, with BL21(DE3)_pSWL157 (/T7^IPTG^-*panat*)) (Supplementary Fig. 13) by the addition of pre-expressed *E. coli* BL21(DE3)_pSWL157 cells (OD_600_ of 20) after growth of the PABA auxotroph expressing ABH60 in the presence of **1**. Both approaches eliminated 4-AB production and generated paracetamol in 12 mg l^−1^ (Supplementary Fig. 10b) and 19 mg l^−1^ (29% yield), respectively, from **1** (Fig. 4c and Supplementary Fig. 14; see Supplementary Figs. 15 and 16 for a comparison with different promoter types and control reactions). To enhance the synthetic utility of paracetamol synthesis from **1** and PET-**1**, a one-pot two-step procedure was optimized where the Lossen rearrangement was first initiated at 50 °C in aqueous phosphate buffer (200 mM, 50 °C, pH 8.0) followed by addition of induced *E. coli* BW25113Δ*pabB*_p354 and *E. coli* BW25113Δ*pabB*_p350 whole cells expressing *panat* and *abh60*, respectively (1:200, 37 °C, OD_600_ of 12.5–25). Under these conditions, quantitative yield of paracetamol was observed from PABA and in 86% from **1** (Fig. 4d). Using the plastic-waste-derived substrate PET-**1** under analogous biotransformation conditions afforded paracetamol in 83% yield. Finally, reducing the arabinose concentration during protein expression enabled a reduction in the ratio of *panat* and *abh60* expressing strains to 1:100 and increased the final yield of paracetamol **10** to 92% from PET-**1** (Fig. 4d). Intensification of this process will focus on in situ biocatalytic depolymerization of industrial PET samples for both bioremediation and bio-upcycling at bioreactor scale to further improve overall productivity and product isolation. This will be accompanied by quantitative sustainability analyses via life cycle assessment to ensure process optimizations achieve maximum environmental sustainability gains. Future work will also focus on further optimization of the de novo pathway to maximize flux into paracetamol biosynthesis using synthetic biology approaches as well as applying the biocompatible Lossen rearrangement to other chemo-enzymatic cascades and fully integrating this new-to-nature reaction within metabolically evolved microorganisms.

## Conclusions

This study reports the discovery of a biocompatible Lossen rearrangement that can be interfaced with cellular metabolism in the bacterium *E. coli*. The non-enzymatic reaction proceeds in the presence of bacterial cells, is non-toxic and is catalysed by mono- or di-basic phosphate at neutral pH, highlighting a multifaceted role of phosphate ions in living cells for pH homeostasis, membrane biosynthesis and now biocompatible non-enzymatic chemistry. The biocompatible reaction also forms primary amine products in vivo via a mechanism that is distinct from known biosynthetic logic and, therefore, provides a useful tool in metabolic engineering for the generation of amine-containing metabolites. We demonstrate through auxotroph rescue how PABA can be generated by the Lossen rearrangement in vivo and used to control microbial growth and chemistry in fermentation and whole-cell reactions. Synthesis of the Lossen rearrangement substrate was achieved from a waste PET bottle and incorporated metabolically to generate biomass and control whole-cell biotransformations. The substrate was syphoned into a de novo biosynthetic pathway to paracetamol, demonstrating the production of this essential medication from plastic waste via a strategy that cannot be achieved using chemical synthesis or biological synthesis alone. Biocompatible chemistry should therefore be considered as complementary to nascent work in enzyme design and engineering for abiotic chemistry and integrated cooperatively as a tool in living cells to expand the synthetic chemistry that is possible within engineered biological systems.

### Reporting summary

Further information on research design is available in the Nature Portfolio Reporting Summary linked to this article.

## Online content

Any methods, additional references, Nature Portfolio reporting summaries, source data, extended data, supplementary information, acknowledgements, peer review information; details of author contributions and competing interests; and statements of data and code availability are available at 10.1038/s41557-025-01845-5.

## Supplementary information

Supplementary Information Materials and methods, Supplementary Figs. 1–16, Tables 1–5 and Scheme 1, and NMR spectroscopy data.

Reporting Summary

## Source data

Source Data Fig. 2 Raw data for Fig. 2.

Source Data Fig. 3 Raw data for Fig. 3.

Source Data Fig. 4 Raw data for Fig. 4.

## Data Availability

All data supporting the findings of this study are available in the article and its Supplementary Information. Source data are provided with this paper.
