# Supplementary material for: A biocompatible Lossen rearrangement in Escherichia coli
Source: Nat Chem. 2025 Jun 23;17(7):1020–6. doi: 10.1038/s41557-025-01845-5 (PMC12226347; doi:10.1038/s41557-025-01845-5)
Supplement: Supplementary file 1 — Materials and methods, Supplementary Figs. 1–16, Tables 1–5 and Scheme 1, and NMR spectroscopy data. [file 41557_2025_1845_MOESM1_ESM.pdf]

nature chemistry

Supplementary information

https://doi.org/10.1038/s41557-025-01845-5

A biocompatible Lossen rearrangement in *Escherichia coli*

In the format provided by the authors and unedited

Contents

S1. General Materials and Methods 2

1.1. Media Recipes and Microbiology 4

1.2. Molecular Biology 4

S2. Plasmid Construction 6

S3. Chemical Synthesis of Lossen Rearrangement Substrates 10

3.1. Synthesis of $PivONH_2 \cdot TfOH$ 10

3.2. Synthesis of *O*-Acyl Benzhydroxamic Acid Derivatives 11

3.2.1. 4-Carboxylate-*O*-Pivaloyl Benzhydroxamic Acid (1) from 4-Formylbenzoic Acid 11

3.2.2. *O*-Acetyl Benzhydroxamic Acid (2) from Benzhydroxamic Acid 12

3.2.3. *O*-Acetyl Benzhydroxamic Acid (3) from *N*-Boc-*N*-Methylhydroxylamine 12

3.2.4. *O*-pivaloyl benzhydroxamic acid (S1) from benzhydroxamic acid 13

3.2.5. *O*-pentafluorobenzoyl benzhydroxamic acid (S2) from benzhydroxamic acid 13

3.2.6. *O*-3,5-bis(trifluoromethyl)benzoyl benzhydroxamic acid (S3) from benzhydroxamic acid 14

3.3. O-Hexanoyl Benzhydroxamic Acid (S4) from Benzhydroxamic Acid 14

3.4. O-Benzoyl Benzhydroxamic Acid (S5) from Benzhydroxamic Acid 14

3.5. O-Succinoyl Benzhydroxamic Acid (S6) from Benzhydroxamic Acid 15

3.6. 4-carboxylate-*O*-pivaloyl benzhydroxamic acid (PET-1) from post-consumer polyethylene terephthalate 15

3.6.2. Hydrolysis of dimethyl terephthalate to monomethyl terephthalate (PET-S8) 16

3.6.3. Coupling of monomethyl terephthalate with *O*-pivaloyl hydroxylamine and hydrolysis to 4-carboxylate-*O*-pivaloyl benzhydroxamic acid (PET-1) 16

3.7. PET hydrolysis and coupling of terephthalic acid with $PivONH_2 \cdot TfOH$ to 4-carboxylate-O-pivaloyl benzhydroxamic acid (PET-1) 17

3.8. Synthesis of ketoacrylates (*E*)-7 and (*Z*)-7 18

S4. Quantitative Phosphate-Catalysed Lossen Rearrangement Reactions in Biological Media 18

S5. Rescue of PABA Auxotrophy 18

5.1. Catalyst Screen 18

5.2. Growth Curve, Plate Count and Toxicity Assay 18

5.3. Native alkene reduction by *E. coli* BW25113Δ*pabB* 19

S6. Paracetamol biosynthesis 19

6.1. Preparation of expressed *E. coli* resting whole cells 19

6.2. Lossen-dependent paracetamol biosynthesis 19

6.3. One-pot Two-step Chemo-enzymatic Cascade Reaction 20

S7. Supplementary Figures 21

S8. NMR Spectroscopy 33

S9. References 44

S1. General Materials and Methods

Unless stated otherwise, all reagents and solvents were purchased from commercial sources and used without further purification. All chemical synthesis was undertaken in round-bottomed flasks. All water (dH_2O) used experimentally was purified with a Suez Select Milli-Q (MQ) purification system (18 MΩ.cm, 0.2 µM filter). PET plastic bottle samples were collected from domestic waste in Edinburgh, UK.

NMR: Proton nuclear magnetic resonance (^{1}H NMR) spectra and carbon nuclear magnetic resonance (^{13}C NMR) spectra were recorded on an AVA400 – (DS) Bruker AVIII 400 (400 MHz, 100 MHz) or a AVA500 – (DS) Bruker AVIII 500 (500 MHz, 125 MHz) at 298 K. Chemical shifts are reported in parts per million (ppm). Data reported as follows: chemical shift, multiplicity (s = singlet, d = doublet, t = triplet, m = multiplet), and coupling constant. J values are given in Hz and reported chemical shifts (δ, ppm) are relative to the deuterated solvent or residual TMS peak.

HPLC: High Performance Liquid Chromatography (HPLC) chromatograms were recorded using an UltiMate 3000 LC System (Thermo Fisher®) equipped with a Thermo Scientific® Hypersil GOLD™ C18 HPLC Column (150 x 3 mm, 3 µm) or a Thermo Scientific® Acclaim™ Organic Acid HPLC Column (150 × 3 mm, 3 µm). Analytes were detected at 206 nm and quantified by comparison of the analyte peak area relative to the peak area of a caffeine internal standard (final concentration = 50 µM, see Figure S11 for calibration curves). All mobile phase solvents were of HPLC grade and purchased from commercial suppliers using the methods outlined (flow rate = 0.4 mL/min, 25 °C, 10 µL sample injection, see Table S1 for the gradient program). Sample preparation for each method was carried out as follows; Method A: 100 µL of the reaction or analyte stock was transferred to a 2 mL microcentrifuge tube before the addition of ACN (150 µL), PBS (200 µL) and caffeine in dH_2O (50 µL, 0.1 mg/ml), or Method B: 500 µL of culture or analyte stock was transferred to a 2 mL microcentrifuge tube before the addition of ACN (300 µL), dH_2O + 0.1% v/v TFA (100 µL) and caffeine in dH_2O (100 µL, 0.1 mg/ml). The resulting mixture was vortexed for 1 min, then centrifuged (17,900 xg) for 10 min before the supernatant was transferred to a 96-well plate for injection.

Table S1. HPLC Gradient Programmes

Method A: C18 HPLC Column used for aniline quantification.

Time (min)	Solvent A: Ammonium acetate (aq., 10 mM) pH 6.6 (%)	Solvent B: ACN (%)
0	95	5
5	95	5
25	50	50
30	50	50
35	95	5
40*(end of method)*	95	5

Method B: Organic Acid HPLC Column used for paracetamol biosynthesis quantification.

Time (min)	Solvent A: dH_2O + 0.1% v/v TFA (%)	Solvent B: ACN + 0.1% v/v TFA (%)
0	100	0
5	100	0
10	90	10
15	75	25
17.5	50	50
20	50	50
22.5	100	0
27.5*(end of method)*	100	0

Flash Chromatography: Flash chromatography was performed on the Teledyne ISCO CombiFlash NextGen® 100 purification system. Normal phase chromatography was performed on a RediSep Rf Gold® Silica gel column. Reverse phase chromatography was performed on a reusable RediSep Rf Gold® C18 Reversed Phase column.

Colorimetric Assay for Quantification of Aromatic Amines: Analyte concentration was determined using a *N*-1-naphthylethylenediamine azo dye colorimetric assay by comparison to known standards (see Figure S2 for calibration curves).[1] Briefly, 6 M HCl (50 µL, aq.) was added to the reaction mixture (50 µL, neat for aniline, 5 × dilution in dH_2O for PABA) in Nunc a flat-bottom 96-well plate before cooling on ice. Next, sequential addition at 3 min intervals of sodium nitrite (5 mM), ammonium sulfamate (50 mM), and *N*-1-naphthylethylenediamine dihydrochloride (5 mM). Next, the mixture was mixed gently, incubated at rt for 10 min and then the absorption at 525 nm was determined using a plate reader. A 5 mM aniline standard was run in parallel and used to normalise for small time deviation.

1.1. **Media Recipes and Microbiology**

General: Stock solutions of glycerol (20 or 50% w/v), $MgSO_4$ (1 M), $CaCl_2$ (1 M) and PABA (10 mM) were prepared by filter sterilisation. Sterile dH_2O was autoclaved at 121 °C for 20 min.

Lysogeny Broth (LB): For 1 L media: Bacto-tryptone (10 g), yeast extract (5 g) and NaCl (10 g) were dissolved in 1 L dH_2O and autoclaved at 121 °C for 20 min. This was stored at rt. Solid media was prepared using the same recipe but with the addition of agar (15 g/L).

M9-glycerol minimal media (M9): For 0.5 L media: Na_2HPO_4 (3 g), KH_2PO_4 (1.5 g), NH_4Cl (500 mg) and NaCl (250 mg) was dissolved in dH_2O (450 mL) and autoclaved at 121 °C for 20 min. Upon cooling to rt, under sterile conditions, $MgSO_4$ (1 mL, final concentration = 2 mM), $CaCl_2$ (50 µL, final concentration = 0.1 mM) and glycerol (12.5 mL, final concentration = 0.5% w/v) were added. Sterile dH_2O was added to a final volume of 0.5 L. The media was stored at 4 °C.

When indicated, this base media was further supplemented with PABA (10 µM). For *Escherichia coli* BW25113Δ*aroC* experiments the media was supplemented, when indicated, to contain *p*-hydroxybenzoic acid (final concentration = 10 µM), 2,3-hydroxybenzoic acid (final concentration = 10 µM), L-tryptophan (final concentration = 100 µM), L-phenylalanine (final concentration = 400 µM), and L-tyrosine (final concentration = 200 µM).

SOC media: For 0.1 L media: Tryptone (200 mg), yeast extract (500 mg), NaCl (58 mg) and KCl (19 mg) was dissolved in 80 mL dH_2O and autoclaved at 121 °C for 20 min. Upon cooling to rt, $MgSO_4$ (1 µL, 10 µM final concentration), $CaCl_2$ (1 µL, final concentration = 10 µM) and glucose (1.8 mL, final concentration = 20 mM) was added. Sterile dH_2O was added to a final volume of 0.1 L. The media was stored at rt.

Potassium phosphate (200 mM, pH 8.0): For 1 L media: K_2HPO_4 (32 g) and KH_2PO_4 (1.7 g) were dissolved in 1 L dH_2O and autoclaved at 121 °C for 20 min. This was stored at rt.

Culturing conditions: Unless otherwise stated *E. coli* was routinely cultured at 37 °C (220 rpm) from frozen glycerol stocks with a 5.1 cm orbital throw. When appropriate media was supplemented with the antibiotics (final concentration = 100 µg/mL for ampicillin, 34 µg/mL for chloramphenicol, and 50 µg/mL for kanamycin, unless stated otherwise). Glycerol stocks were prepared from starter cultures by 2 × dilution in 50% w/v filter-sterilised aq. glycerol and stored at -70 °C. *E. coli* starter cultures were prepared in in a 50 mL tube with appropriate supplements and incubated at 37 °C (220 rpm) for 16 h (LB) and 48 h (M9) ensuring $OD_{600} > 1.0$. Cell density was determined by measuring optical density (OD_{600}) using a DS-11 FX Spectrophotometer (DeNovix®) at 600 nm. Growth curves were obtained using a BMG FLUOstar® Omega Microplate Reader by measuring OD_{600} every 15 min at 37 °C (500 rpm).

***E. coli* K12 Knock-out Strains** *Escherichia coli* BW25113 knock-out strains of the *E. coli* K-12 Keio Collection Δ*pabA* (CGSC Strain #10483), Δ*pabB* (CGSC Strain #9507), and Δ*aroC* (CGSC Strain #9865) were obtained from Horizon Discovery Group Ltd.

1.2. **Molecular Biology**

General: *E. coli* DH5α was used for all recombinant plasmid DNA construction. All chemically competent cells were prepared using calcium chloride[2] and were transformed using heat-shock at 42 °C for 1 min then on ice for 2 min followed by a recovery step at 37 °C for 1 h in SOC medium before being plated and incubated on antibiotic-selective LB agar plates overnight at 37 °C. Synthetic genes were synthesised using GeneArt™ (Thermo Scientific). All oligonucleotide primers were synthesised by Integrated DNA Technologies. Products from PCR were gel purified with a Zymoclean Gel DNA Recovery Kit (Zymo Research). Recombinant plasmid DNA were purified using QIAprep plasmid Miniprep Kit (Qiagen) from *E. coli* DH5α. Restriction digests, unless stated, were carried out overnight

at rt using FastDigest™ (Thermo Fisher) restriction enzymes and FastDigest™ Green Buffer. Gibson Assembly was carried out using Gibson Assembly® Master Mix (New England Biolabs, NEB) according to the manufacturer's protocol. All generated plasmids were confirmed by colony PCR and Sanger sequencing (Azenta). Modular cloning was performed using JUMP (Joint Universal Modular Plasmids) backbones and plasmids.[3]

Agarose Gel Electrophoresis: For agarose gel electrophoresis, agarose (1% w/v) TAE gels containing SYBR Safe™ were used to analyse samples by comparing to GeneRuler™ 1 kB ladder (Thermo Scientific). Gels were run in 1 × TAE buffer at 100 V for 35 min. For SDS-PAGE, 12-well 12% acrylamide Bis-Tris NuPAGE gels (Thermo Scientific) were used to analyse samples by comparing to GeneRuler™ 1 kB ladder (Thermo Scientific). (see Figure S12 and S13 for representative gels). Gels were run in MES buffer (Novagen) at 200 V for 30 min and stained using Simply Blue™ SafeStain (Thermo Fisher).

PCR: All PCR reactions were carried out using ProFlex™ PCR System (Thermo Fisher). PCR reactions were performed using Phusion High-Fidelity DNA Polymerase (NEB). OneTaq (New England Biolabs, NEB) was used for colony PCRs. Colony PCR reactions were carried out using the standard protocol stated in Table S2.

Table S2. Standard colony PCR method

		Temp (°C)	Time (s)
	Initial Denaturation	95	300
30 cycles	Denaturation	95	30
	Annealing	50-68	30
	Extension	68	60 (per kb)
	Final Extension	68	300
	Hold	4	

Removal of the Kanamycin Resistance Gene: Plasmid pCP20 (containing the Flp recombinase gene from *Saccharomyces cerevisiae*) was obtained from the Coli Genetic Stock Center (CGSC). Chemically competent *E. coli* BW25113Δ*pabB* were transformed with plasmid pCP20. Cells were plated on LB-ampicillin agar and incubated overnight at 30 °C. A single colony was used to inoculate LB (5 mL) that was incubated overnight at 43 °C (220 rpm) before being streaked out onto LB agar and incubated overnight at 30 °C. Removal of the antibiotic resistance cassette was confirmed by streaking a single colony onto LB-kanamycin, -ampicillin and -antibiotic-free agar.

S2. Plasmid Construction

DNA sequences coding for 4-aminobenzoate hydroxylase (ABH60) from *Agaricus bisporus* and an arylamine *N*-acetyltransferase (PANAT) from *Pseudomonas aeruginosa* were synthesised as specified in[4] by Invitrogen (Table S3). Both coding sequences were synthesised as modular cloning level 0 parts as specified in[3], with PANAT being designed as a part type "O" part by replacing the starting ATG codon with an in-frame fusion site.

New parts coding for inducible promoters were generated as shown in Table S3. The arabinose inducible promoter (named Pbad(*araC*)) was amplified from *E. coli* MG1655 using primers in Table S5 including the gene coding for AraC. The anhydrotetracycline-inducible promoter (named Ptet(*tetR*)) was designed by combining the optimised *tetR*-controlled promoter and *tetR* optimised in[5]. Equivalently, an IPTG-inducible promoter was designed combining *lacI* and a LacI-controlled promoter, resulting in part Ptac(*lacI).* Ptet(*tetR*) and Ptac(*lacI)* were synthesised by Integrated DNA Technologies Ltd.

Table S3. List of plasmids generated in this study (see Figure S9 for plasmid maps).

Plasmid Name	Description
pSWL112	pJUMP29-1A backbone (pBR322/Rop origin, KanR marker) with Pbad(*araC*) polycistronic cassette coding ABH60 and PANAT.
pSWL156	pCS-His6-CAV1 backbone (p15a origin of replication, CmR marker, J23100 promoter) coding ABH60.
pSWL157	pET22b backbone (pB322/ROP OriV, AmpR, T7 IPTG induction) coding PANAT.
pSWL349	pJUMP29-1A backbone (pBR322/Rop origin, KanR marker) with Ptac(*lacI*) cassette coding ABH60 with RBS-pET, and L3S1P51_T terminator.
pSWL350	pJUMP29-1A backbone (pBR322/Rop origin, KanR marker) with Pbad(*araC*) cassette coding ABH60 with RBS-pET, and L3S1P51_T terminator.
pSWL351	pJUMP29-1A backbone (pBR322/Rop origin, KanR marker) with Ptet(*tetR*) cassette coding ABH60 with RBS-pET, and L3S1P51_T terminator.
pSWL354	pJUMP29-1B backbone (pBR322/Rop origin, KanR marker) with Pbad(*araC*) cassette coding PANAT with B0032-MV_RN as RBS, and B0015_CT terminator.
pSWL355	pJUMP29-1B backbone (pBR322/Rop origin, KanR marker) with Ptet(*tetR*) cassette coding PANAT with B0032-MV_RN as RBS, and B0015_CT terminator.

Plasmid pSWL112 combined ABH60 and PANAT parts in one operon. The plasmid was built in a level 1 assembly using backbone pJUMP29-1A, Pbad(*araC*) promoter, terminator L1U1H08_CT, RBS part RBS-pET_R (for ABH60) and a special part encoding RBS-pET used to connect ABH60 and PANAT parts. The special part was generated by annealing the following oligos (as indicated in[1]):

CGTCTCGGTCTCAGCTTTGTTTAACTTTAAGAAGGAGATATACAATGGTAGCCTGAGACC TGAGACG and CGTCTCAGGTCTCAGGCTACCATTGTATATCTCCTTCTTAAAGTTAAACAAAGCTGAGAC CGAGACG.

Plasmid pSWL156 was built by amplifying by ABH60 with primers in Table S5 and inserted in the NdeI and AvaI-cut backbone of pCS-His6-CAV1 using Gibson assembly. Plasmid pSWL157 was generated by amplifying PANAT with primers in Table S5 and inserted in the in the NcoI- and NdeI-

cut backbone of pET22b using Gibson assembly. Gibson assemblies were ligated using a 3:1 molar ratio insert:vector and standard conditions. All new plasmids were verified using colony PCR, restriction digestion and Sanger sequencing.

Table S4. Nucleotide sequences (5’ to 3’) of protein coding sequences used in this study.

**ABH60**
ATGGTTCAGGGTGAACGTAGCCATATTGCAATTATTGGTGCAGGTATTGTTGGTCTGGC<br>ATTTGCAGTTGCACTGAATGCACTGGATAAAGAACATAAATTTGCCATCGATCTGTAT<br>GAAGCAACACCGGAACTGGCAGAAATTGGCGCAGGCATTAATGTTTGGCCTCTGACAC<br>TGAGCATTCTGAAAGAAATGGGTCTGCATCAGACACTGATTCCGTTTTTCGATCATTAT<br>CCGGATCTGGAACGTCGTGTTATTTTTGGTCTGCGTAAAGCCGATGAGAAAAATGGCT<br>TTCATGTGTATGATGTGATGAATGAAGGTGGTGCACTGCGTATTCATCGTGCCGATCTG<br>CAGCGTGGTCTGATTCAGCATCTGCCGCTGAGCAAAAGCAATAAAGTTCATATTAACA<br>CCCCGTGCACCTTTCATCTGAATCATCGTCTGAAAGATTATACCCGTGATGCCAGCGAA<br>GATTTTGGTCGTATTAAACTGCATTTTGATGGTAAACCGAGCCGTGAATGTGATGTTCT<br>GATTGGTGCCGATGGTATTCATAGCACCGTTCGTCAGCTGTTTCTGAGCCGTCTGCCGA<br>GTCCGGAACGTTATGATAAATATCGTAAACCGGTGTGGTCAGGTCTGGTTGCATATCG<br>TGGTCTGGTTAGCCGTGAAGATCTGGAAGAAACCTATCCGGGTCATCGTGCACTGACC<br>CATCCGGGTCTGATTTATACCGGTAAAACCCGTTATGTTACCATTTATCCGGTTAGCGG<br>TGGCAAATTCATTAATGTGGTTGCAATTGCGCGTGATACCAGCAATGATACCACCGTTT<br>GGAAAGGTCCGTGGAAAGTTGAAGTTACCCAAGAAGAATTTTTCCACGTCTATCAGGG<br>CTTTGATGAAGAAGTTCTGGCACTGATTCATTGCATTAAAAAGCCGACCAAATGGGCA<br>CTGCATGTTCTGGATCATCTGGATATCTTTAGCAAACAGCAGGTTTTTCTGATGGGTGA<br>TGCAGCACATGCAATGCTGCCGCATTTAGGTGCCGGTGCAACCGTTGGTATTGAAGAT<br>GCATATATTCTGGCAAGCATGCTGACACATCAGAGCACCAGCCGTCCGCTGAATAGCG<br>AAAAGATTAAACTGATTAGCACCATCTATAACACCGTTCTGGTTCCGCATGCAACCCG<br>TATGAGCAAACTGACCAATGATACAGGTCATCTGCTGGATCTGACCGCACCGGGTTTT<br>GATTTAGAACGTTATACCCTGGGTGATCGTATTCCGCTGGAAACCCTGATTAATGCATT<br>TCGTCAGGTGGAACGTAATTGGATTTGGAGCAGCAGCGATCCGGAAGAAGATCGTCGT<br>AAAGTAGAAGATCTGCTTGAAAGTTGGAGCGGTCCGCGTCTGAGCATTAGCCATCAGT<br>AA
**PANAT**
ATGACTCCTCTGACCCCTGAACAGACCCATGCGTATCTGCATCATATTGGCATTGATGA<br>TCCTGGTCCTCCGAGCCTGGCGAATTTAGATCGCCTGATCGATGCGCATTTACGCCGCG<br>TTGCGTTTGAAAACCTGGATGTGCTGCTGGATCGCCCGATTGAAATTGATGCGGACAA<br>AGTGTTTGCGAAAGTGGTGGAAGGTAGCCGTGGCGGCTATTGCTTTGAACTGAACAGC<br>CTGTTTGCGCGTCTGTTACTGGCGTTAGGCTATGAACTGGAACTGCTGGTGGCACGTGT<br>TCGTTGGGGTTTACCTGATGATGCGCCGTTAACCCAGCAGAGCCATTTAATGCTGCGCC<br>TGTATCTGGCGGAAGGCGAATTTCTGGTGGATGTGGGCTTTGGTTCAGCGAACCCGCC<br>TCGTGCATTACCTTTACCGGGCGATGAAGCAGATGCGGGTCAGGTTCATTGTGTTCGCC<br>TGGTGGATCCTCATGCGGGCTTATATGAAAGCGCGGTGCGTGGTCGTAGCGGTTGGTT<br>ACCTCTGTATCGCTTTGATCTGCGCCCGCAGCTGTGGATTGATTATATTCCGCGCAACT<br>GGTATACCAGCACCCATCCGCATAGCGTGTTTCGCCAGGGTCTGGGCGCGGCGATTAC<br>CGAAGGTGATCTGCGTCTGACCTTAGCGGATGGTCTGTTTGGTCAGCGTGCGGGTAAC<br>GGTGAAACCCTGCAGCGTCAGTTACGCGATGTGGAAGAACTGCTGGATATTCTGCAGA<br>CCCGCTTTCGTTTACGTCTGGATCCGGCGTCAGAAGTTCCTGCACTGGCGCGTCGTTTA<br>GCGGGCTTAATTAGCGCGCTCGAGTAG

**Pbad(*araC*) promoter**
GGAGTTATGACAACTTGACGGCTACATCATTCACTTTTTCTTCACAACCGGCACGAAAC<br>TCGCTCGGGCTGGCCCCGGTGCATTTTTTAAATACTCGCGAGAAATAGAGTTGATCGTC<br>AAAACCAACATTGCGACCGACGGTGGCGATAGGCATCCGGGTAGTGCTCAAAAGCAG<br>CTTCGCCTGACTAATGCGTTGGTCCTCGCGCCAGCTTAAGACGCTAATCCCTAACTGCT<br>GGCGGAAAAGATGTGACAGACGCGACGGCGACAAGCAAACATGCTGTGCGACGCTGG<br>CGATATCAAAATTGCTGTCTGCCAGGTGATCGCTGATGTACTGACAAGCCTCGCGTAC<br>CCGATTATCCATCGGTGGATGGAGCGACTCGTTAATCGCTTCCATGCGCCGCAGTAAC<br>AATTGCTCAAGCAGATTTATCGCCAGCAGCTCCGAATAGCGCCCTTCCCCTTGCCCGGC<br>GTTAATGATTTGCCCAAACAGGTCGCTGAAATGCGGCTGGTGCGCTTCATCCGGGCGA<br>AAGAAACCCGTATTGGCAAATATTGACGGCCAGTTAAGCCATTCATGCCAGTAGGCGC<br>GCGGACGAAAGTAAACCCACTGGTGATACCATTCGCGAGCCTCCGGATGACGACCGTA<br>GTGATGAATCTCTCCTGGCGGGAACAGCAAAATATCACCCGGTCGGCAGACAAATTCT<br>CGTCCCTGATTTTTCACCACCCCCTGACCGCGAATGGTGAGATTGAGAATATAACCTTT<br>CATTCCCAGCGGTCGGTCGATAAAAAAATCGAGATAACCGTTGGCCTCAATCGGCGTT<br>AAACCCGCCACCAGATGGGCGTTAAACGAGTATCCCGGCAGCAGGGGATCATTTTGCG<br>CTTCAGCCATACTTTTCATACTCCCACCATTCAGAGAAGAAACCAATTGTCCATATTGC<br>ATCAGACATTGCCGTCACTGCGTCTTTTACTGGCTCTTCTCGCTAACCCAACCGGTAAC<br>CCCGCTTATTAAAAGCATTCTGTAACAAAGCGGGACCAAAGCCATGACAAAAACGCGT<br>AACAAAAGTGTCTATAATCACGGCAGAAAAGTCCACATTGATTATTTGCACGGCGTCA<br>CACTTTGCTATGCCATAGCATTTTTATCCATAAGATTAGCGGATCCTACCTGACGCTTT<br>TTATCGCAACTCTCTACTGTTTCTCCATACT
**Ptet(*tetR*) promoter**
GGAGGGGAGACCAGAAACAAAAAAAGGCCCCCCGTTAGGGAGGCCTTCAATAATTGG<br>CCAATTAGGATCCTTATCAGGACCCACTTTCACATTTAAGTTGTTTTTCTAATCCGCAT<br>ATGATCAATTCAAGGCCGAATAAGAAGGCTGGCTCTGCACCTTGGTGATCAAATAATT<br>CGATAGCTTGTCGTAATAATGGCGGCATACTATCAGTAGTAGGTGTTTCCCTTTCTTCT<br>TTAGCGACTTGATGCTCTTGATCTTCCAATACGCAACCTAAAGTAAAATGCCCCACAG<br>CGCTGAGTGCATATAATGCATTCTCTAGTGAAAAACCTTGTTGGCATAAAAAGGCTAA<br>TTGATTTTCGAGAGTTTCATACTGTTTTTCTGTAGGCCGTGTACCTAAATGTACTTTTGC<br>TCCATCGCGATGACTTAGTAAAGCACATCTAAAACTTTTAGCGTTATTACGTAAAAAA<br>TCTTGCCAGCTTTCCCCTTCTAAAGGGCAAAAGTGAGTATGGTGCCTATCTAACATCTC<br>AATGGCTAAGGCGTCGAGCAAAGCCCGCTTATTTTTTACATGCCAATACAATGTAGGC<br>TGCTCTACACCTAGCTTCTGGGCGAGTTTACGGGTTGTTAAACCTTCGATTCCGACCTC<br>ATTAAGCAGCTCTAATGCGCTGTTAATCACTTTACTTTTATCTAATCTGGACATATTCA<br>CCACCCTGAATTGACTCTCTTCCGGGCGCTATCATGCCATACCGCGAAAGGTTTTGCGC<br>CATTCGATGGCGCGCCGCCATAAGCTTATCATCGATAAGCTTGCATGCCTTTTCAGCAG<br>GACGCACTGACCTCCCTATCAGTGATAGAGATTGACATCCCTATCAGTGATAGAGATA<br>CTGAGCACTACT

**Ptac(lacI) promoter**
GGAGTCACGAACCCTGCCCGCTTTCCAGTCGGGAAACCTGTCGTGCCAGCTGCATTAA<br>TGAATCGGCCAACGCGCGGGGAGAGGCGGTTTGCGTATTGGGCGCCAGGGTGGTTTTT<br>CTTTTCACCAGTGACACGGGCAACAGCTGATTGCCCTTCACCGCCTGGCCCTGAGAGA<br>GTTGCAGCAAGCGGTCCACGCTGGTTTGCCCCAGCAGGCGAAAATCCTGTTTGATGGT<br>GGTTAACGGCGGGATATAACATGAGCTATCTTCGGTATCGTCGTATCCCACTACCGAG<br>ATATCCGCACCAACGCGCAGCCCGGACTCGGTAATGGCGCGCATTGCGCCCAGCGCCA<br>TCTGATCGTTGGCAACCAGCATAGCAGTGGGAACGATGCCCTCATTCAGCATTTGCAT<br>GGTTTGTTGAAAACCGGACATGGCACTCCAGTCGCCTTCCCGTTCCGCTATCGGCTGAA<br>TTTGATTGCGAGTGAGATATTTATGCCAGCCAGCCAGACGCAGACGCGCCGAGACAGA<br>ACTTAATGGGCCCGCTAACAGCGCGATTTGCTGGTGACCCAATGCGACCAGATGCTCC<br>ACGCCCAGTCGCGTACCATCTTCATGGGAGAAAATAATACTGTTGATGGGTGTCTGGT<br>CAGAGACATCAAGAAATAACGCCGGAACATTAGTGCAGGCAGCTTCCACAGCAATGG<br>CATCCTGGTCATCCAGCGGATAGTTAATGATCAGCCCACTGACGCGTTGCGCGAGAAG<br>ATTGTGCACCGCCGCTTTACAGGCTTCGACGCCGCTTCGTTCTACCATCGACACCACCA<br>CGCTGGCACCCAGTTGATCGGCGCGAGATTTAATCGCCGCGACAATTTGCGACGGCGC<br>GTGCAGGGCCAGACTGGAGGTGGCAACGCCAATCAGCAACGACTGTTTGCCCGCCAGT<br>TGTTGTGCCACGCGGTTGGGAATGTAATTCAGCTCCGCCATAGCCGCTTCCACTTTTTC<br>CCGCGTTTTCGCAGAAACGTGGCTGGCCTGGTTCACCACGCGGGAAACGGTCTGATAA<br>GAGACACCGGCATACTCTGCGACATCGTATAACGTTACTGGTTTGGCTTTCATATTCAC<br>CACCCTGAATTGACTCTCTTCCGGGCGCTATCATGCCATACCGCGAAAGGTTTTGCGCC<br>ATTCGATGGCGCGCCGCTCCTGTTGACAATTAATCATCGGCTCGTATAATGTGTGGAAT<br>TGTGAGCGCTCACAATTTACT

Table S5. Oligonucleotide primers used for plasmid construction.

**Plasmid or part construction**	**Sequence 5'-3'**
Pbad(*araC*) promoter	CGTCTCaGTCTGCGATGtttGGTCTCtGGAGttaTGACAACTTGACGGCTAC
	CGTCTCaGGTCGCGATGaaaGGTCTCaAGTATGGAGAAACAGTAGAGAGTTG
pSWL156	CTTTAAGAAGGAGATATACATATGGTTCAGGGTGAACGTAG
	TGGCCAGGACCCAACGCTGCTTACTGATGGCTAATGCTCAG
pSWL157	CTTTAAGAAGGAGATATACATATGGCCACTCCTCTGACC
	TCCGAATTAATTCCGATATCCTACTCGAGCGCGCTAATTAAG

S3. Chemical Synthesis of Lossen Rearrangement Substrates

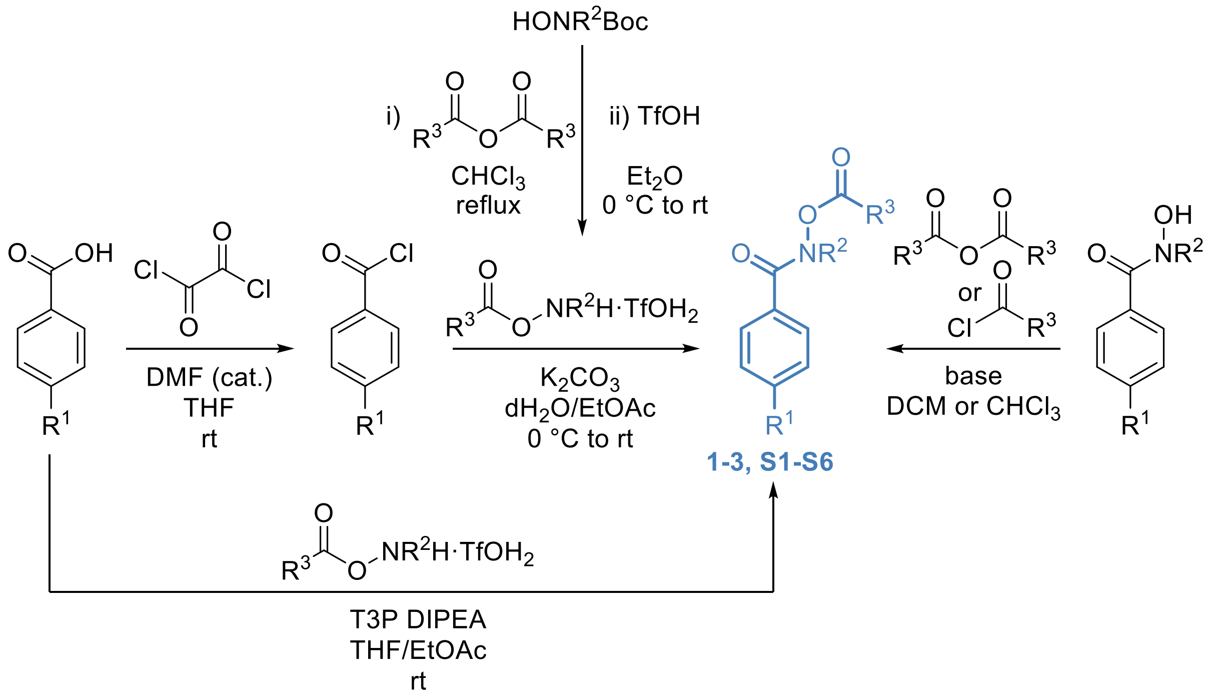

Scheme S1. General synthetic routes to Lossen rearrangement substrates **1-3** and **S1-S6**

3.1. Synthesis of PivONH$_2$·TfOH

PivONH$_2$·TFOH

The following procedure was carried out as reported.[6] Trimethylacetic anhydride (3.66 mL, 18.0 mmol, 1.2 equiv.) was added to a stirred solution of *N*-Boc-hydroxylamine (2.00 g. 15.0 mmol, 1.0 equiv.) in $CHCl_3$ (40 mL) before being heated under reflux. After 16 h, the reaction mixture was allowed to cool to rt before being quenched with sat. $NaHCO_3$ (aq.) and then the organic layer was separated before being dried over anhydrous $MgSO_4$, filtered, and concentrated by rotary evaporation to afford a colourless solid. The crude was dissolved in stirred anhydrous Et_2O (40 mL) in a fresh flask and cooled to 0 °C before the dropwise addition of trifluoromethanesulfonic acid (1.33 mL, 15.0 mmol, 1.0 equiv.). The resulting mixture was left to warm to rt for 1 h before the addition of 60 mL heptane and further cooling at 0 °C for 1 h to encourage precipitation. Finally, the precipitate was vacuum filtered, then washed with heptane (10 mL) to afford PivONH$_2$·TfOH (3.37 g, 12.6 mmol, 84% yield) as a white solid. ^{1}H NMR (500 MHz, DMSO-d$_6$) δ 5.50 (br. s, 2H) 1.21 (s, 9H). ^{13}C NMR (125 MHz, DMSO-d$_6$) δ 176.2, 125.0-117.3 (q, J = 320 Hz), 38.4, 27.0. Data corresponds to published data.[6]

3.2. **Synthesis of *O*-Acyl Benzhydroxamic Acid Derivatives**

3.2.1. **4-Carboxylate-*O*-Pivaloyl Benzhydroxamic Acid (1) from 4-Formylbenzoic Acid**

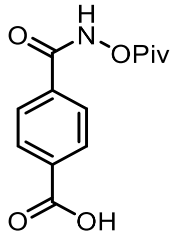

1

In a dry flask and under a nitrogen atmosphere, anhydrous DMF (*ca.* 0.1 mL) and then oxalyl chloride (0.38 mL, 4.49 mmol, 2.3 equiv.) was added to a stirred solution of 4-formylbenzoic acid (300 mg, 2.00 mmol, 1.0 equiv.) in anhydrous THF (7.5 mL) at 0 °C. The reaction mixture was left to warm to rt and after 16 h the reaction was concentrated by rotary evaporation. In a fresh flask, the crude acid chloride intermediate in minimal EtOAc was added dropwise to a stirred solution of K_2CO_3 (830 mg, 6.01 mmol, 3.0 equiv.) and $PivONH_2$·TfOH (533 mg, 1.99 mmol, 1.0 equiv.) in 1:2 dH_2O:EtOAc (36 mL) at 0 °C. The reaction mixture was left to warm to rt and after 16 h, the organic phase was removed before the aq. phase was further extracted with EtOAc (2 × 10 mL). The combined organic fractions were dried over anhydrous $MgSO_4$, filtered, and concentrated by rotary evaporation to afford a yellow solid that was purified by normal phase flash chromatography (20:1 to 1:1 hexane + 0.5% AcOH:EtOAc + 0.5% AcOH), to afford the *O*-pivaloyl hydroxamic acid intermediate as a yellow solid (319 mg). In a fresh flask, the intermediate was solubilised in MeCN (3.28 mL) and added dropwise to a stirred solution of periodic acid (319 mg, 1.40 mmol, 0.70 equiv.) in MeCN (8.2 mL) at 0 °C before dropwise addition of pyridinium chlorochromate (4.9 mg, 0.02 mmol, 1 mol%) in MeCN (4.92 mL). After 10 min the mixture was allowed to warm to rt and stirred for 2 h before being diluted with EtOAc (20 mL) and then washed with 1:1 brine:water (2 × 5 mL), sat. $NaHSO_3$ (2 × 5 mL, aq.) and with brine (2 × 5 mL). The organic layer was then dried over anhydrous $MgSO_4$, filtered, and concentrated by rotary evaporation before being purified by reverse phase flash chromatography (20:1 then 1:1 dH_2O + 0.1% v/v TFA:MeCN + 0.1% v/v TFA), to afford **1** (63 mg, 0.2 mmol, 12% yield) as a white solid after freeze drying. ^{1}H NMR (500 MHz, DMSO-d_6) δ 12.46 (br. s, 1H), 8.11 – 7.97 (m, 2H), 7.93 – 7.88 (m, 2H), 1.30 (s, 9H). ^{13}C NMR (125 MHz, DMSO-d_6) δ 176.1, 167.1, 164.3, 135.3, 134.4, 130.0, 128.1, 38.3, 27.3. HRMS (ESI) m/z: $[M + H]^+$ calc. for $C_{13}H_{16}NO_5$: 266.1023; Found 266.1020.

3.2.2. ***O*-Acetyl Benzhydroxamic Acid (2) from Benzhydroxamic Acid**

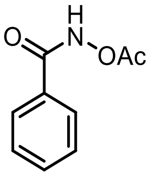

2

Sodium hydroxide (48 mg, 1.2 mmol, 1.2 equiv.) was added to a stirred solution of benzhydroxamic acid (137 mg, 1.00 mmol, 1.0 equiv.) in DCM (3 mL) at rt for 5 min before acetic anhydride (113 µL, 1.20 mmol, 1.2 equiv.) was added dropwise. The resulting solution was stirred at rt for 16 h before being diluted with DCM (15 mL), quenched with sat. $NaHCO_3$ (10 mL, aq.) and then extracted with DCM (3 × 15 mL). The combined organic fractions were dried over anhydrous $MgSO_4$, filtered, and concentrated by rotary evaporation to reveal a crude that was purified by normal phase chromatography (7:3 hexane:EtOAc) to afford **2** (49 mg, 0.3 mmol, 27% yield) as a colourless crystalline solid. ^{1}H NMR (500 MHz, DMSO) δ 12.30 (br. s, 1H), 7.83 – 7.80 (m, 2H), 7.64 – 7.59 (m, 1H), 7.55 – 7.50 (m, 2H), 2.24 (s, 3H).^{13}C NMR (126 MHz, DMSO) δ 169.1, 168.6, 132.7, 131.5, 129.1, 127.8, 18.6. HRMS (ESI) m/z: $[M + H]^+$ calc. for $C_9H_{10}NO_3$: 180.0655; Found 180.0667.

3.2.3. ***O*-Acetyl Benzhydroxamic Acid (3) from *N*-Boc-*N*-Methylhydroxylamine**

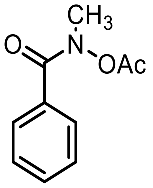

3

Acetic anhydride (693 µL, 7.33 mmol, 1.2 equiv.) was added to a stirred solution of *N*-Boc-*N*-methylhydroxylamine (900 mg, 6.12 mmol, 1.0 equiv.) in $CHCl_3$ (50 mL) before being heated under reflux. After 16 h, the reaction was quenched with sat. $NaHCO_3$ (20 mL, aq.), before the aq. layer was removed and the organic fraction was washed with further sat. $NaHCO_3$ (20 mL, aq.) and then dried over anhydrous $MgSO_4$, filtered, and concentrated by rotary evaporation to afford a colourless solid crude (1.0 g). In a fresh flask, this intermediate was solubilised in anhydrous Et_2O (25 mL) and cooled to 0 °C before the dropwise addition of trifluoromethanesulfonic acid (540 µL, 6.10 mmol, 1.0 equiv.). The mixture was allowed to warm to rt and after 1 h the mixture was separated with heptane (40 mL) before being dried over anhydrous $MgSO_4$, filtered, and concentrated by rotary evaporation to afford a brown oil (2.0 g). In a fresh flask, half of this second intermediate was taken forward and added to a stirred solution of K_2CO_3 (1.26 g, 9.12 mmol, 3.0 equiv.) in 1:2 dH_2O:EtOAc (45 mL) before cooling to 0 °C and then dropwise addition of benzoyl chloride (351 µL, 3.02 mmol, 1.0 equiv.). The reaction mixture was left to warm to rt and after 16 h the organic phase was separated and the aq. phase was further extracted with EtOAc (2 × 10 mL). The combined organic fractions were dried over anhydrous $MgSO_4$, filtered, and concentrated by rotary evaporation to afford a white crude that was purified by normal phase chromatography (10:1 hexane:EtOAc) to afford **2** (17 mg, 88 µmol, 1.4% yield) as a colourless oil. ^{1}H NMR (500 MHz, MeOD) δ 7.59 – 7.56 (m, 2H), 7.54 – 7.51 (m, 1H), 7.49 – 7.45 (m, 2H), 3.40 (s, 3H), 2.02 (s, 3H). ^{13}C NMR (126 MHz, MeOD) δ 171.0, 168.3, 133.2, 130.9, 128.0, 127.5, 36.2, 16.7. HRMS (ESI) m/z: $[M+H]^+$ calc. for $C_{10}H_{12}NO_3$ 194.0812; Found 194.0814.

3.2.4. ***O*-pivaloyl benzhydroxamic acid (S1) from benzhydroxamic acid**

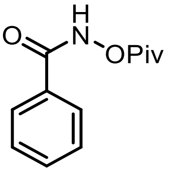

S1

NaOH (48 mg, 1.2 mmol, 1.2 equiv.) was added to a solution of benzhydroxamic acid (137 mg, 1.00 mmol, 1.0 equiv.) in DCM (3 mL) and stirred at rt for 5 min before the dropwise addition of trimethylacetic anhydride (203 µL, 186 mg, 1.00 mmol, 1.0 equiv.). After 16 h the reaction mixture was diluted with DCM (15 mL) and then quenched with sat. $NaHCO_3$ (10 mL, aq.) before extraction with DCM (3 × 15 mL). The combined organic fractions were dried over anhydrous $MgSO_4$, filtered, and concentrated by rotary evaporation to reveal the crude product that was purified by normal phase chromatography (10:1 then 4:1 hexane:EtOAc), to afford **S1** (77 mg, 0.35 mmol, 35% yield) as a colourless solid. ^{1}H NMR (500 MHz, DMSO) δ 12.26 (br. s, 1H), 7.84 – 7.79 (m, 2H), 7.64 – 7.59 (m, 1H), 7.55 – 7.50 (m, 2H), 1.30 (s, 9H). ^{13}C NMR (126 MHz, DMSO) δ 176.2, 164.9, 132.7, 131.6, 129.1, 127.8, 38.3, 27.3. HRMS (ESI) m/z: $[M + H]^+$ calc. for $C_{12}H_{16}NO_3$ 222.1125; Found 222.1135

3.2.5. ***O*-pentafluorobenzoyl benzhydroxamic acid (S2) from benzhydroxamic acid**

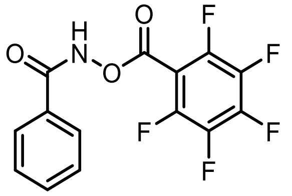

S2

KO^{t}Bu (22 mg, 1.08 mmol, 1.1 equiv.) was added to a stirred solution of benzhydroxamic acid (137 mg, 1.00 mmol, 1.0 equiv.) in 10 mL Et_2O (10 mL). After 20 min, 2,3,4,5,6-pentafluorobenzoyl chloride (144 µL, 1.00 mmol, 1.0 equiv.) was added dropwise and left at rt for 24 h, before the reaction mixture was diluted with EtOAc (15 mL) and then quenched with sat. $NaHCO_3$ (10 mL, aq.). The resulting mixture was then extracted with EtOAc (3 × 15 mL) and then the organic fractions were combined, dried over anhydrous $MgSO_4$, filtered, and then concentrated by rotary evaporation to reveal the crude product that was purified by normal phase chromatography (10:1 hexane:EtOAc), to afford **S2** (113 mg, 0.341 mmol, 34% yield) as a colourless solid. ^{1}H NMR (500 MHz, DMSO) δ 13.02 (br. s, 1H), 7.91 – 7.85 (m, 2H), 7.71 – 7.62 (m, 1H), 7.62 – 7.52 (m, 2H). ^{13}C NMR (126 MHz, DMSO) δ 165.3, 157.9, 133.2, 130.8, 129.3, 128.0 (environments for *C*–F are not observed). HRMS (ESI) m/z: $[M + H]^+$ calc. for $C_{14}H_7F_5NO_3$ 332.03441; Found 332.0353.

3.2.6. ***O*-3,5-bis(trifluoromethyl)benzoyl benzhydroxamic acid (S3) from benzhydroxamic acid**

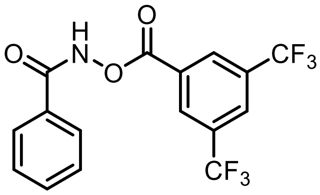

S3

In a dry flask K_2CO_3 (138 mg, 1.00 mmol, 1.0 eq.) was added to a solution benzhydroxamic acid (137 mg, 1.00 mmol, 1.0 equiv.) in Et_2O (10 mL) at 0 °C, before dropwise addition of 3,5-bis(trifluoromethyl)benzoyl chloride (181 µL, 1.00 mmol, 1.0 eq.) that was stirred for 1 min at 0 °C. The reaction was then allowed to warm to rt and, after 2 h, dH_2O (10 mL) was added before extraction with Et_2O (2 × 15 mL). The combined organic fractions were dried over anhydrous $MgSO_4$, filtered, and concentrated by rotary evaporation to afford **7** (310 mg, 0.82 mmol, 82% yield) without further purification as a colourless solid. ^{1}H NMR (500 MHz, DMSO) δ 12.96 (br. s, 1H), 8.65 – 8.59 (m, 1H), 7.89 (app. d, J = 7.1 Hz, 2H), 7 7.70 – 7.63 (m, 1H), 7.60 – 7.54 (m, 2H). ^{13}C NMR (126 MHz, DMSO) δ 162.57, 133.02, 131.83 (q, J = 33.9 Hz), 131.06, 130.47 (app. d, J = 3.5 Hz), 130.15 – 130.01 (m), 129.26, 128.59 – 128.44 (m), 127.96, 123.21 (q, J = 273.3 Hz). HRMS (ESI) m/z: $[M + H]^+$ calc. for $C_{16}H_{10}F_6NO_3$: 378.0559; Found 378.0559.

3.3. **O-Hexanoyl Benzhydroxamic Acid (S4) from Benzhydroxamic Acid**

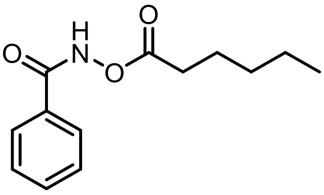

S4

NaOH (48 mg, 1.2 mmol, 1.2 equiv.) was added to a stirred solution of benzhydroxamic acid (137 mg, 1.00 mmol, 1.0 equiv.) in DCM (6 mL) at rt for 10 min before hexanoic anhydride (214 mg, 1.00 mmol, 1.0 equiv.) was added. The resulting solution was stirred at rt for 16 h before being diluted with DCM (20 mL), quenched with sat. $NaHCO_3$ (10 mL, aq.) and then extracted with DCM (3 × 15 mL). The combined organic fractions were dried over anhydrous $MgSO_4$, filtered, and concentrated by rotary evaporation to afford **S4** (190 mg, 0.81 mmol, 81% yield) without further purification as a colourless crystalline solid. ^{1}H NMR (500 MHz, DMSO-d_6) δ 12.28 (br. s, 1H), 7.85 – 7.80 (m, 2H), 7.64 – 7.59 (m, 1H), 7.54 – 7.51 (m, 2H), 1.63 (p, J = 7.4 Hz, 2H), 1.40 – 1.28 (m, 4H), 0.91 – 0.88 (m, 3H). ^{13}C NMR (126 MHz, DMSO) δ 171.8, 165.0, 132.7, 131.6, 129.1, 127.8, 31.4, 30.9, 24.6, 22.2, 14.3. HRMS (ESI) m/z: $[M + H]^+$ calc. for $C_{13}H_{18}NO_3$: 236.1281; Found 236.1279.

3.4. **O-Benzoyl Benzhydroxamic Acid (S5) from Benzhydroxamic Acid**

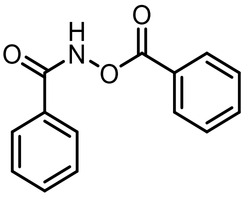

S5

NaOH (48 mg, 1.2 mmol, 1.2 equiv.) was added to a stirred solution of benzhydroxamic acid (137 mg, 1.00 mmol, 1.0 equiv.) in DCM (3 mL) at rt for 5 min before benzoic anhydride (226 mg, 1.00 mmol, 1.0 equiv.) was added. The resulting solution was stirred at rt for 16 h before being diluted with DCM (15 mL), quenched with sat. $NaHCO_3$ (10 mL, aq.) and then extracted with DCM (3 × 15 mL). The combined organic fractions were dried over anhydrous $MgSO_4$, filtered, and concentrated by rotary evaporation to afford **S5** (181 mg, 0.75 mmol, 75% yield) without further purification as a colourless crystalline solid. ^{1}H NMR (500 MHz, DMSO-d_6) δ 12.62 (br. s, 1H), 8.12 – 8.07 (m, 2H), 7.90 – 7.86 (m, 2H), 7.80 – 7.77 (m, 1H), 7.67 – 7.61 (m, 2H), 7.59 – 7.53 (m, 2H). ^{13}C NMR (126 MHz, DMSO) δ 165.3, 164.8, 134.9, 132.8, 131.5, 130.0, 129.7, 129.2, 127.9, 127.4. HRMS (ESI) m/z: $[M + H]^+$ calc. for $C_{14}H_{12}NO_3$: 242.0812; Found 242.0817.

3.5. **O-Succinoyl Benzhydroxamic Acid (S6) from Benzhydroxamic Acid**

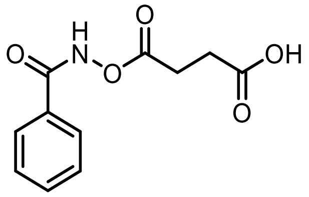

S6

NaOH (48 mg, 1.2 mmol, 1.2 equiv.) was added to a stirred solution of benzhydroxamic acid (137 mg, 1.00 mmol, 1.0 equiv.) in DCM (3 mL) at rt for 5 min before succinic anhydride (50 mg, 0.50 mmol, 0.5 equiv.) and resulting solution was stirred at rt for 48 h. Further succinic anhydride was added during the overall time course of the reaction in a stepwise manner with 50 mg (0.50 mmol, 0.5 equiv.) being introduced at 6 h, 24 h, 26 h and 28 h. The reaction mixture was then diluted with DCM (20 mL), quenched with sat. $NaHCO_3$ (10 mL, aq.) and then extracted with DCM (1 × 15 mL) and EtOAc (2 × 15 mL). The resulting aqueous phase was acidified with 1 M HCl and then extracted EtOAc (3 × 15 mL). The combined organic fractions were dried over anhydrous MgSO4, filtered, and concentrated by rotary evaporation to reveal the crude product. Using a Büchner funnel, the crude product was washed with 1 M HCl (aq., 20 mL) and then DCM (10 mL) to afford **S6** (58 mg, 0.24 mmol, 24% yield) as a colourless crystalline solid. ^{1}H NMR (500 MHz, DMSO-d_6) δ 12.41 (br. s, 1H), 7.86 – 7.79 (m, 2H), 7.64 – 7.58 (m, 1H), 7.55 – 7.49 (m, 2H), 2.77 – 2.74 (m, 2H), 2.60 – 2.57 (m, 2H). ^{13}C NMR (126 MHz, DMSO) δ 173.4, 171.3, 164.9, 132.7, 131.5, 129.1, 127.8, 28.9, 27.0. HRMS (ESI) m/z: $[M + H]^+$ calc. for $C_{11}H_{12}NO_5$: 238.0710; Found 238.0701.

3.6. **4-carboxylate-*O*-pivaloyl benzhydroxamic acid (PET-1) from post-consumer polyethylene terephthalate**

3.6.1. Methanolysis of PET plastic bottle to dimethyl terephthalate **PET-S7**

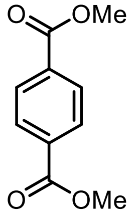

PET-S7

The following procedure was carried out as reported.[7] In a dry flask, K_2CO_3 (144 mg, 1.04 mmol, 0.2 equiv.) was added to a stirred mixture of anhydrous MeOH (10.5 mL) and anhydrous DCM (16.6 mL), before being heated to 25 °C. After 15 min, PET flakes (1 g, ca. 5.2 mmol, surface area = ~0.50 cm^2,

manually cut Lidl® Naturis Pure Squeezed Orange Juice Smooth post-consumer PET plastic bottle washed with ethanol and acetone then dried at 90 °C for 4 h) were added. After 20 h, the mixture was vacuum filtered, and the precipitate was washed with MeOH (2 × 20 mL). The filtrate was concentrated by rotary evaporation before the addition of dH_2O (25 mL) followed by cooling to 0 °C. After stirring for 1 h the precipitate was collected by filtration and washed with cold dH_2O (4 × 10 mL) to afford **S7** (719 mg, 3.70 mmol, 71% yield) as a white solid. ^{1}H NMR (500 MHz, $CDCl_3$) δ 8.12 (s, 4H), 3.97 (s, 6H). ^{13}C NMR (126 MHz, $CDCl_3$) δ 166.3, 134.0, 129.6, 52.4.

3.6.2. **Hydrolysis of dimethyl terephthalate to monomethyl terephthalate (PET-S8)**

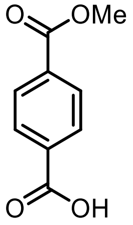

PET-S8

The following procedure was carried out as reported.[8] In a dry flask, 710 mg (3.66 mmol, 1.0 equiv.) of **S4** was added to a stirred solution of 205 mg (3.65 mmol, 1.0 equiv.) KOH in 3.7:4.1 anhydrous methanol:toluene (15.6 mL) before being heated to 50 °C. After 4 h the reaction mixture was cooled to rt before being concentrated by rotary evaporation and then filtered and washed with DCM (3 × 10 mL) The crude solid was acidified with 6 M HCl (aq.) before being extracted into EtOAc (4 × 15 mL). The combined organic fractions were dried over anhydrous $MgSO_4$, filtered, and concentrated by rotary evaporation to afford **S8** (449 mg, 2.49 mmol, 68% yield) as a white solid. ^{1}H NMR (400 MHz, DMSO-d_6) δ 8.07 (app. s, 4H), 3.89 (app. s, 3H). ^{13}C NMR (126 MHz, DMSO-d_6) δ 167.0, 166.1, 135.3, 133.6, 130.1, 129.8, 52.9.

3.6.3. **Coupling of monomethyl terephthalate with *O*-pivaloyl hydroxylamine and hydrolysis to 4-carboxylate-*O*-pivaloyl benzhydroxamic acid (PET-1)**

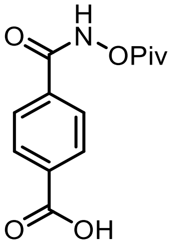

PET-1

In a dry flask, anhydrous DMF (ca. 0.1 mL) then oxalyl chloride (427 µL, 5.05 mmol, 2.0 equiv.) was added dropwise to a solution of **S5** (449 mg, 2.49 mmol, 1.0 equiv.) in THF (10 mL) at 0 °C. The reaction mixture was left to warm to rt and, after 16 h, the crude acyl chloride intermediate was concentrated by rotary evaporation. In a fresh flask, K_2CO_3 (1.0 g, 7.5 mmol, 3.0 equiv.) and then $PivONH_2$·TfOH (665 mg, 2.49 mmol, 1.0 equiv.) were dissolved in 1:2 dH_2O:EtOAc (36 mL) before being cooled to 0 °C and the dropwise addition of the crude intermediate in minimal EtOAc. The reaction mixture was left to warm to rt and after 5 h the organic phase was removed before the aq. phase was extracted with EtOAc (2 × 10 mL). The combined organic fractions were washed with 1 M HCl (aq., 2 × 10 mL) before being dried over anhydrous $MgSO_4$, filtered, and concentrated by rotary evaporation to afford the intermediate ester. This intermediate was added to a fresh dry flask containing a stirred solution of KOH (269 mg, 4.79 mmol, 1.8 equiv.) in anhydrous 5.3:6 methanol:toluene (11.3 mL). The mixture was heated to 50 °C for 5 h before being allowed to cool to rt, and filtration of the resulting precipitate, that was subsequently acidified with 2 M HCl (aq.) and extracted with EtOAc (4

× 10 mL). The combined organic fractions were dried over anhydrous $MgSO_4$, filtered, and concentrated by rotary evaporation before being purified by reverse phase flash chromatography (20:1 then 1:1 dH_2O:MeCN + 0.1% v/v TFA) to afford **PET-1** (106 mg, 0.40 mmol, 16% yield) as a white solid after freeze drying. (^{1}H NMR (500 MHz, DMSO-d_6) δ 12.46 (br. s, 1H), 8.10 – 8.03 (m, 2H), 7.94 – 7.88 (m, 2H), 1.30 (s, 9H). ^{13}C NMR (126 MHz, DMSO) δ 176.1, 167.1, 164.3, 135.3, 134.4, 130.0, 128.1, 38.3, 27.3. HRMS (ESI) m/z: $[M + H]^+$ calc. for $C_{13}H_{16}NO_5$: 266.1023; Found 266.1020.

3.7. PET hydrolysis and coupling of terephthalic acid with $PivONH_2$·TfOH to 4-carboxylate-O-pivaloyl benzhydroxamic acid (PET-1)

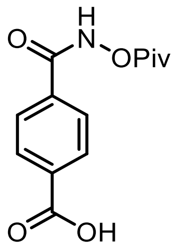

PET-1

PET plastic bottle waste was cleaned and cut into approximately 2 cm^2 segments. PET fragments (10.9 g, ca. 56.7 mmol) were added to a mixture of EtOH (72 mL) and of NaOH solution (288 mL, 10% w/v) and stirred at reflux for 10 h before being filtered under vacuum. The filtrate was acidified using HCl solution (37% aq. 100 mL) and the white precipitate was collected and dried by vacuum filtration (5.58 g, 83% purity by ^{1}H NMR using TMB as internal standard, 27.9 mmol, 49% yield). The resulting powder was used without further purification. Coupling was undertaken as reported[9] but with the following modifications. Propanephosphonic acid anhydride (1.19 mL, 2.00 mmol, 2.20 equiv., 50% w/v in EtOAc) and diisopropylethylamine (0.95 mL, 5.40 mmol, 6.00 equiv.) was added to a stirred solution of PET-derived terephthalic acid (150 mg, 0.90 mmol, 1.00 equiv.) in THF (9 mL) at 0 °C for 30 mins before $PivONH_2$·TfOH (240 mg, 0.90 mmol, 1.0 equiv.) was added. The reaction was allowed to warm to rt and, after 16 h, the reaction mixture was diluted with PBS (30 mL). The pH of the mixture was adjusted to 9 with 1 M HCl (aq.) before being extracted with EtOAc (3 × 20 mL). The pH of the resultant aqueous phase was then adjusted to pH 6.5 with 1 M HCl (aq.) and extracted with EtOAc (3 × 20 mL). The combined organic fractions were dried over anhydrous $MgSO_4$, filtered, and concentrated by rotary evaporation to reveal the crude product. Using a Büchner funnel, the crude product was washed with 1 M HCl (aq., 10 mL) and then DCM (5 mL) to afford **PET-1** (27 mg, 0.10 mmol, 11% yield from terephthalic acid) as a colourless solid. ^{1}H NMR (500 MHz, DMSO-d_6) δ 13.26 (br. s, 1H) 12.46 (br. s, 1H), 8.09 – 8.04 (m, 2H), 7.94 – 7.88 (m, 2H), 1.30 (s, 9H). ^{13}C NMR (126 MHz, DMSO) δ 176.1, 167.1, 164.2, 135.3, 134.4, 130.0, 128.1, 38.3, 27.3.

3.8. **Synthesis of ketoacrylates (*E*)-7 and (*Z*)-7**

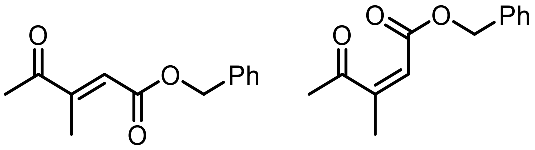

(*E*)-**7** (*Z*)-**7**

The following procedure was carried out as reported.[10] 2,3-butanedione (431 mg, 5.01 mmol, 1.0 equiv.) and benzyl (triphenylphosphoranylidene)acetate (2.05 g, 5.01 mmol, 1.0 equiv.) were dissolved in DCM (25 mL) and stirred at rt. After 48 h, the reaction mixture was concentrated by rotary evaporation before purification of the crude (*E/Z* isomer ratio of 7:3) by normal phase chromatography (10:1 pet ether:EtOAc) to afford (*E*)-**7** and (*Z*)-**7** as colourless oils: (*E*)-**7** (496 mg, 2.27 mmol, 45% yield) and (*Z*)-**7** (81 mg, 0.37 mmol, 7.4% yield). (*E*)-**7** ^{1}H NMR (500 MHz, $CDCl_3$) δ 7.44 – 7.35 (m, 5H), 6.64 (q, *J* = 1.4 Hz, 1H), 5.25 (s, 2H), 2.40 (s, 3H), 2.26 (d, *J* = 1.4 Hz, 3H). ^{13}C NMR (126 MHz, $CDCl_3$) δ 199.8, 166.0, 151.1, 135.5, 128.7, 128.5, 126.0, 66.7, 26.2, 13.2. HRMS (ESI) m/z: $[n+H]^+$ calc. for $C_{13}H_{15}O_3$: 219.10157; Found 219.1024:$[M + Na]^+$; calc. for $C_{13}H_{14}NaO_3$: 241.0835; Found 241.0842. (*Z*)-**7** ^{1}H NMR (500 MHz, $CDCl_3$) δ 7.43 – 7.33 (m, 5H), 5.77 (q, *J* = 1.7 Hz, 1H), 5.16 (s, 2H), 2.35 (s, 3H), 2.02 (d, *J* = 1.7 Hz, 3H). ^{13}C NMR (126 MHz, $CDCl_3$) δ 206.1, 164.9, 157.8, 135.5, 128.6, 128.4, 116.7, 66.6, 28.6, 20.3. HRMS (ESI) m/z: $[M + H]^+$; calc. for $C_{13}H_{15}O_3$: 219.10157; Found 219.1022: $[M + Na]^+$; calc. for $C_{13}H_{14}NaO_3$: 241.0835; Found 241.0840.

S4. Quantitative Phosphate-Catalysed Lossen Rearrangement Reactions in Biological Media

In 1.5 mL microcentrifuge tubes, **1**, **2**, **3**, **4**, or **S1-S6** (5 µL of a 500 mM stock in DMSO, final concentration = 5 mM) was added to M9 or PBS (495 µL), in triplicate, before being vortexed and then incubated horizontally at 37 °C (220 rpm) for 72 h before being analysed by HPLC or colorimetric assay. Control reactions were performed identically except that the stated media components were omitted.

S5. Rescue of PABA Auxotrophy

5.1. **Catalyst Screen**

An *E. coli* starter culture in M9 was diluted (10^5 ×) in M9 and used to inoculate fresh M9 (10 µL in 5 mL) in triplicate containing *O*-pivaloyl hydroxamic acid **1** (5 µL of 10 mM DMSO stock, final concentration = 10 µM), and chemical catalyst (5 µL of 1 mM DMSO stock, final concentration = 1 µM) that were then incubated at 37 °C (220 rpm) for 72 h whilst monitoring OD_{600}. In parallel, positive controls (without catalyst, as well as **1** replaced with PABA only) and a negative control (without **1** or catalyst) was performed under the same conditions.

5.2. **Growth Curve, Plate Count and Toxicity Assay**

An *E. coli* starter culture in M9 was diluted (10^5 ×) in M9 and used to inoculate M9 (0.4 µL in 200 µL) in triplicate containing *O*-pivaloyl hydroxamic acid **1** or **10** (0.2 µL of 10 mM DMSO stock, final concentration = 10 µM), and incubated at 37 °C (500 rpm) for 72 h in sterile Nunc flat-bottom 96-well plate whilst monitoring OD_{600}. In parallel, negative control (without substrate) and positive control (**1** replaced with PABA) were performed under the same conditions. Serial dilution (10^4-10^7 ×) of the resulting cultures in sterile dH_2O were plated (200 µL) on LB-kanamycin agar and incubated overnight at 37 °C before colonies were counted (see Figure S5).

For toxicity assessment the conditions were modified such that the concentration of **1** was increased only (see Figure S8)

5.3. **Native alkene reduction by *E. coli* BW25113Δ*pabB***

A glycerol stock of *E. coli* BW25113Δ*pabB* was used to inoculate M9 (5 mL for dimethyl maleate **5** and 2 mL for keto-acrylate **7**) containing **1** (10 mM DMSO stock, final concentration = 10 µM) and were incubated at 37 °C (220 rpm) for 24 h before each culture was transferred to sterile glass Hungate tubes. Next, **5** or **7** (2 M DMSO stock, final concentration = 2 mM) were added before the tubes were sealed with a sterile butyl rubber stopper and incubated horizontally at 37 °C (220 rpm) for 24 h. Reactions were run in triplicate and were cooled at 4 °C for 15 min before extraction into $CDCl_3$ containing 3 mM 1,3,5-trimethoxybenzene as internal standard (for **5**, 1 mL of the reaction was extracted into 3 × 333 µL, and for **7** the entire reaction volume was extracted into 3 × 666 µL) by being vortexed for 1 min and then centrifuged (17,900 xg) for 3 min. The combined organic fractions were dried over anhydrous $NaSO_4$, filtered and then analysed by ^{1}H NMR (see Figures S6 and S7).

For time courses, a glycerol stock of *E. coli* BW25113Δ*pabB* was used to inoculate M9 (40 mL) that was that were aliquoted (1 mL) into 15 mL centrifuge tubes and incubated at 37 °C (220 rpm). After 24 h **5** (2 M DMSO stock, final concentration = 2 mM) was added to the remaining cultures and reactions were incubated horizontally at 37 °C (220 rpm) for 16 h whilst measuring OD_{600}, as well as substrate and reduced product concentration by ^{1}H NMR at the stated timepoints.

S6. Paracetamol biosynthesis

6.1. **Preparation of expressed *E. coli* resting whole cells**

A starter culture using the appropriate strain of *E. coli* was used to inoculate LB (2 mL in 100 mL) in a sterile 500 mL Erlenmeyer flask that was incubated at 37 °C (200 rpm). Once the cell density reached OD_{600} 0.6-0.8, the culture was cooled to 4 °C for 10 min before the addition of the inducer (IPTG (50 µL, 1 M sterilised-dH_2O stock, final concentration = 0.5 mM) for pSWL157, arabinose (100 µL, 20% w/v sterilised-dH_2O stock, final concentration = 0.1% w/v) for pSWL350 and pSWL354) and anhydrotetracycline (25 µL, 2 mM 1:1 filter sterilised-dH_2O:EtOH stock, final concentration = 0.5 µM) for pSWL351 and incubated at rt (220 rpm). When indicated, pSWL350 cells were induced with 0.5% arabinose. After 20 h, the cells were harvested by centrifugation (8,166 xg) for 10 min, washed with media (20 mL) by centrifugation (8,166 xg) for 10 min and then the cell pellet was resuspended in 20 mL media. (*E. coli* BL21(DE3)_pSWL157 whole cells were resuspended in M9 media and *E. coli* BW25113Δ*pabB*_pSWL350 and *E. coli* BW25113Δ*pabB*_pSWL354 whole cells were resuspended in potassium phosphate (200 mM, pH 8.0)). Cell density was measured followed by further centrifugation (8,166 xg) for 10 min and resuspended in the required media for OD_{600} = 100.

6.2. **Lossen-dependent paracetamol biosynthesis**

Glycerol stocks of *E. coli* BW25113Δ*pabB* with the appropriate plasmids were used to inoculate M9 media (1 mL) in a 15 mL tubes in triplicate containing *O*-pivaloyl hydroxamic acid **1** (1 µL, 500 mM DMSO stock, final concentration = 0.5 mM), and incubated at 37 °C (220 rpm). ABH expression was induced after 24 h with arabinose (5 µL, 20% arabinose, for pSWL112 and pSWL350) or IPTG (0.5 µL, 1 M sterilised-dH_2O stock for pSWL349) or anhydrotetracycline (0.25 µL, 2 mM 1:1 filter sterilised-dH_2O:EtOH stock, final concentration = 0.5 µM, for pSWL351). PANAT expression was induced after 72 h with same concentrations of arabinose (for pSWL354) or anhydrotetracycline (for pSWL355) (see Figure S10 and S15). For cultures containing expression plasmid pSWL112, ABH and PANAT expression was induced after 24 h with the same concentrations of arabinose. HPLC analysis (see Section S1 for details) was undertaken at 76 h for the cultures containing expression cassettes for ABH60 and PANAT.

For cultures containing expression plasmids for ABH only, 800 µL of the initial culture was transferred to a 2 mL microcentrifuge tube before the addition of resuspended *E. coli* BL21(DE3)_pSWL157 expressed whole cells (200 µL, OD_{600} = 100) (see Section S6.1). The cultures were further incubated at

37 °C (220 rpm) for 24 h, before HPLC analysis (see Section S1). In parallel, negative control (without **1**) and positive control (**1** replaced with PABA) were performed under the same conditions (see Figure S16).

6.3. **One-pot Two-step Chemo-enzymatic Cascade Reaction**

In a 1.5 mL microcentrifuge tube, potassium phosphate (49.5 µL, 200 mM, pH 8.0) was added along with **1** (0.5 µL of a 50 mM stock in DMSO, final concentration = 0.5 mM) before being vortexed and then incubated horizontally at 50 °C (220 rpm) for 48 h. Resuspended *E. coli* BW25113Δ*pabB*_pSWL350 and *E. coli* BW25113Δ*pabB*_pSWL354 expressed whole cells (OD_{600} = 100) were added to the reactions at the specified ratio and optical density along with glycerol (5 µL, aq., 20% w/v) (see Section S6.1). Unless stated, the reactions were run in triplicate. Potassium phosphate (200 mM, pH 8.0) was added to a final volume of 80 µL and the reaction mixtures were further incubated at 37 °C (220 rpm) for 24 h, before HPLC analysis (see Section S1).

S7. Supplementary Figures

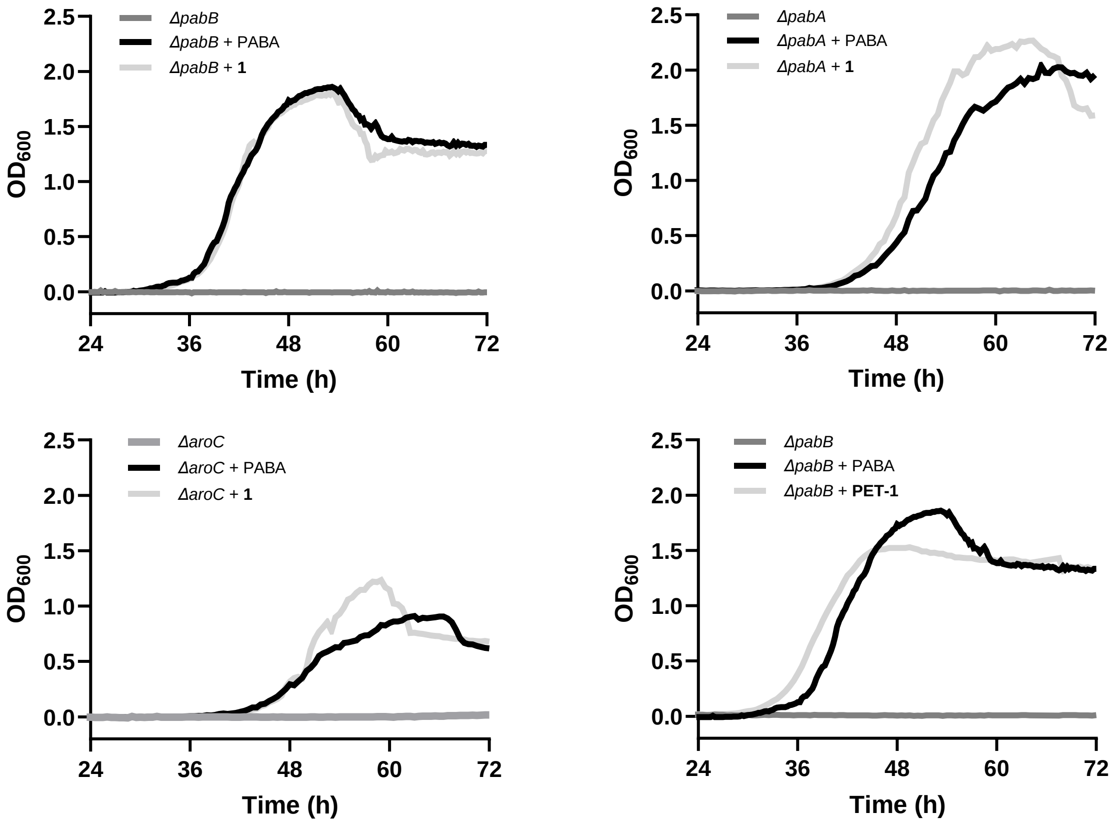

Figure S1. Lossen-dependent auxotrophic rescue growth curves of *E. coli* BW25113 mutants containing **1** or PET-**1** or PABA. All data are shown as triplicate experiments displaying the mean value at each data point.

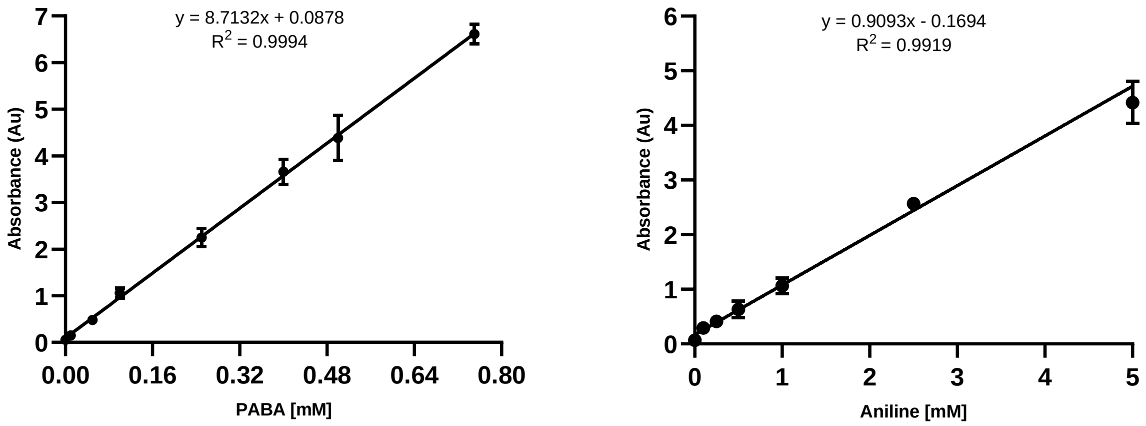

Figure S2. Quantitative colorimetric assay calibration curves.

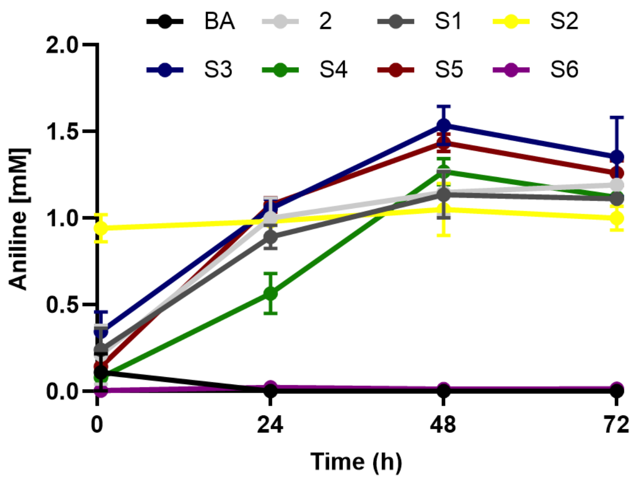

Figure S3. Time course of aniline production from Lossen rearrangement substrates **2**, **4**, and **S1-6** (5 mM) incubated in PBS at 37 °C. All data are shown as triplicate experiments to one standard deviation and all concentrations were determined using colorimetric assay.

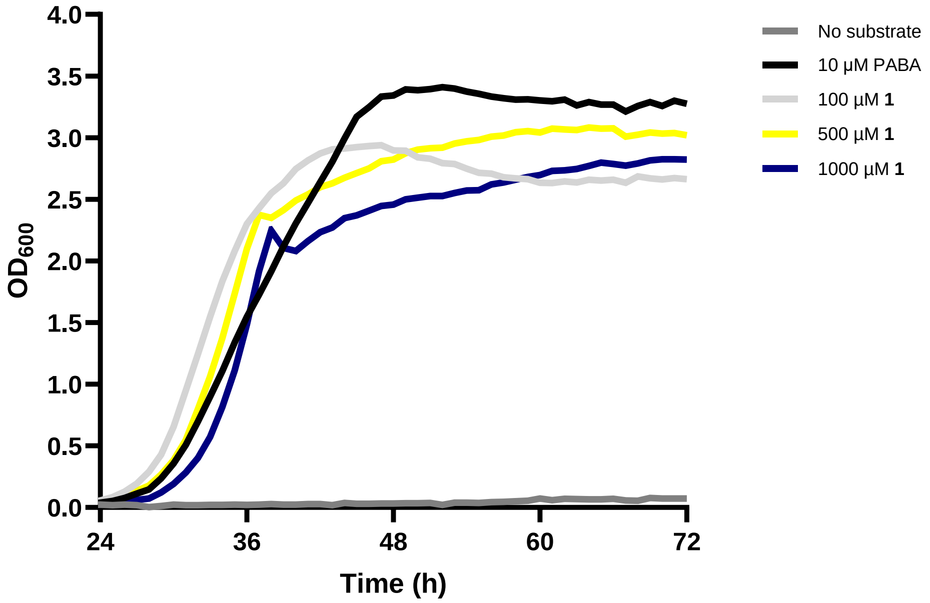

Figure S4. Growth curve of *E. coli* BW25113Δ*pabB* in the presence of **1** or PABA. All data are shown as triplicate experiments displaying the mean value at each data point.

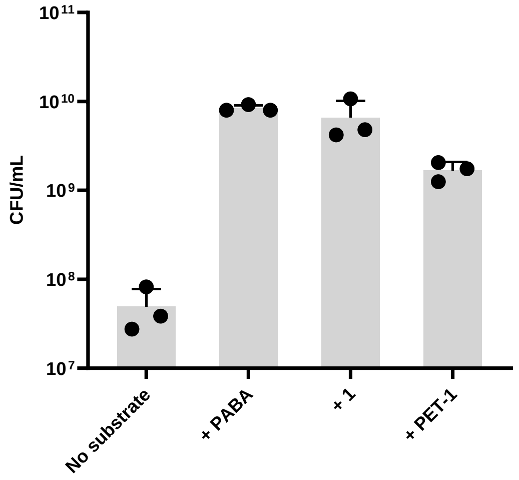

Figure S5. A plate count assay of *E. coli* BW25113Δ*pabB* auxotrophic rescue experiments cultures grown over 72 h containing to **1**/PET-**1** (10 µM). All data are shown as triplicate experiments to one standard deviation.

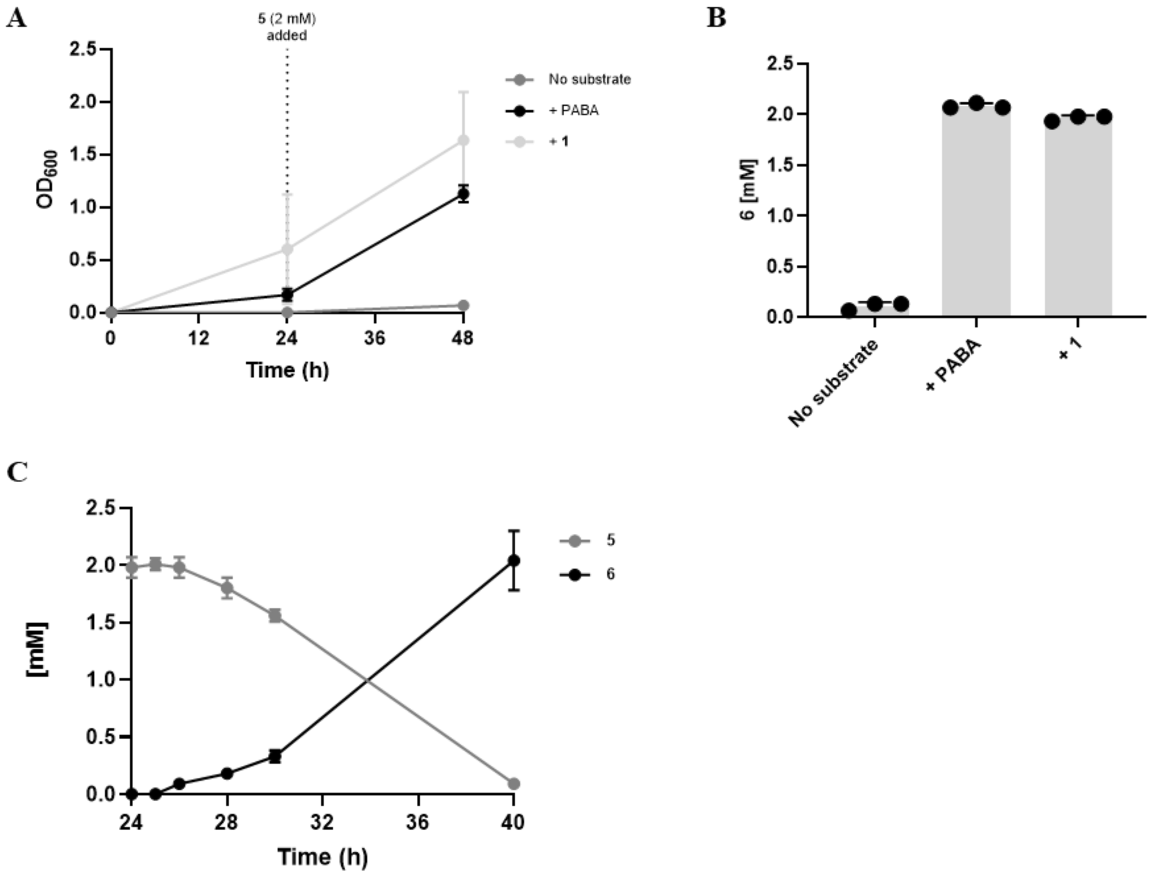

Figure S6. Reduction of dimethyl maleate **5** using Lossen-dependent *E. coli* BW25113Δ*pabB* growth. **A** Growth curve with **5** (2 mM) added at 24 h. **B** Concentration of dimethyl succinate **6** after 48 h when adding **5** (2 mM) at 24 h. **C** Time course of the conversion of **5** to **6**. All data are shown as triplicate experiments to one standard deviation.

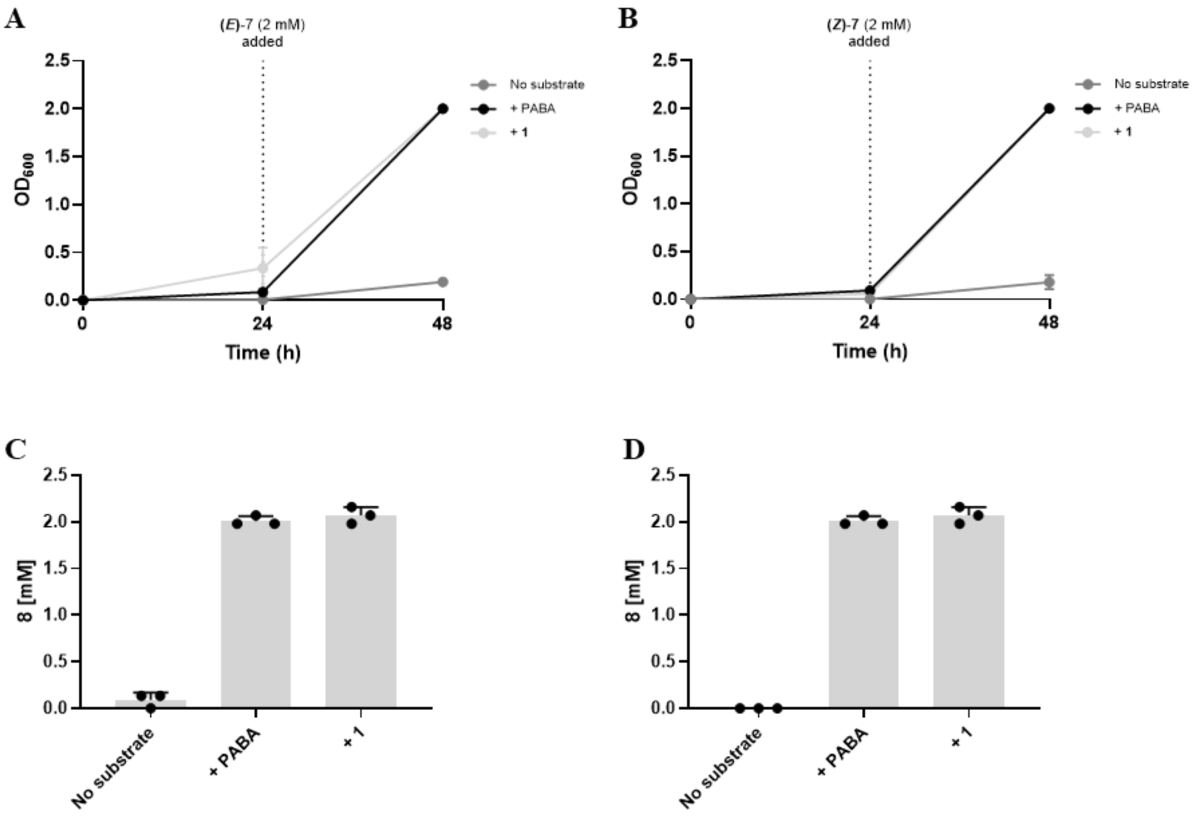

Figure S7. Reduction of keto-acrylates using Lossen-dependent *E. coli* BW25113Δ*pabB* growth. **A** Growth curve with (*E*)-**7** (2 mM) added at 24 h. **B** Growth curve with (*Z*)-**7** (2 mM) added at 24 h. **C** Concentration of **8** at 48 h when using (*E*)-**7** (2 mM). **D** Concentration of **8** at 48 h when adding (*Z*)-**7** (2 mM) at 24 h. All data are shown as triplicate experiments to one standard deviation.

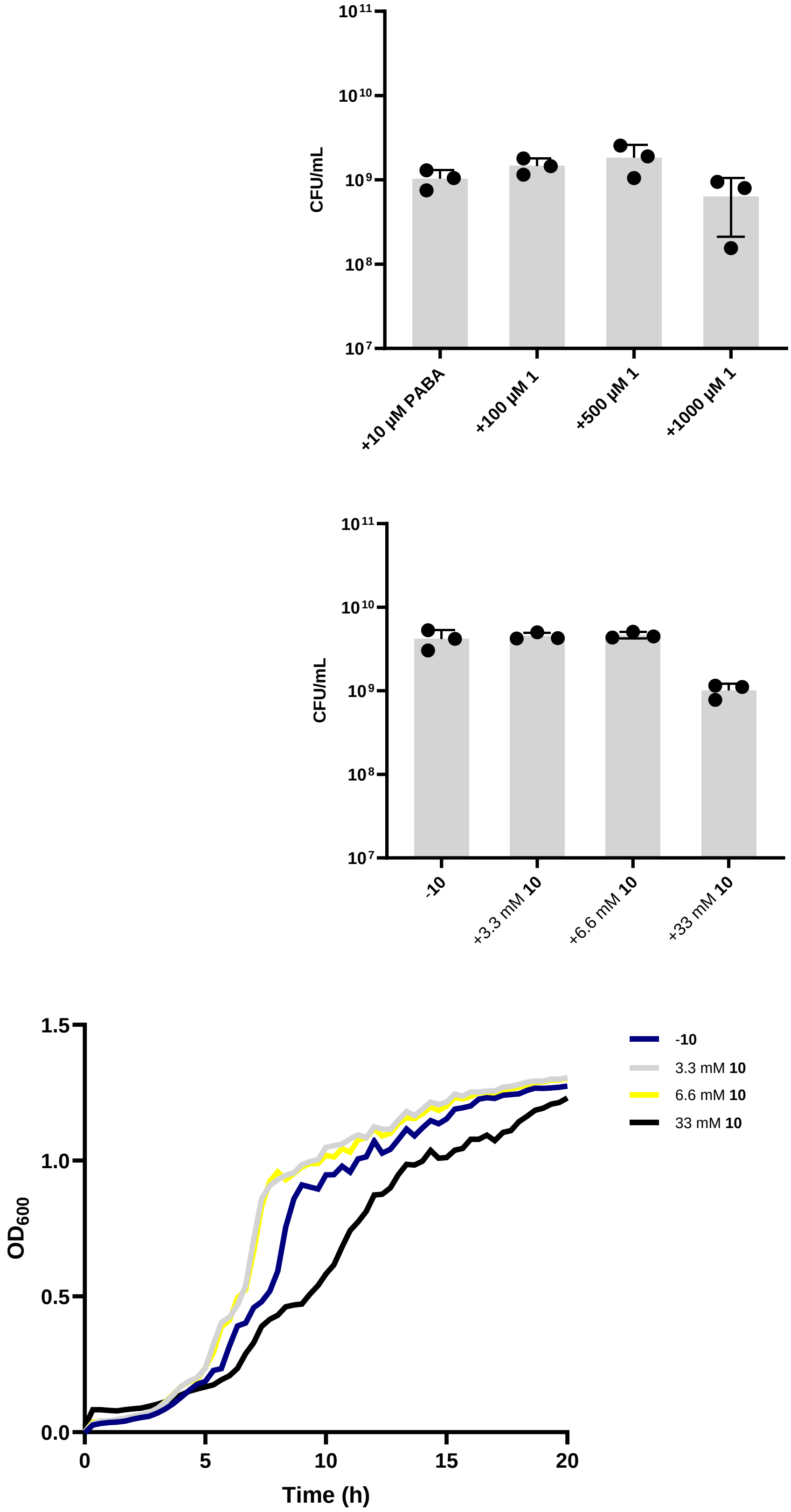

Figure S8. A plate count assay of *E. coli* BW25113Δ*pabB* cultures grown over 72 h containing **1** or **10** and growth curves of *E. coli* BW25113Δ*pabB* incubated in the presence of **10**. All data are shown as triplicate experiments to one standard deviation apart from the growth curve which displays the mean value at each data point.

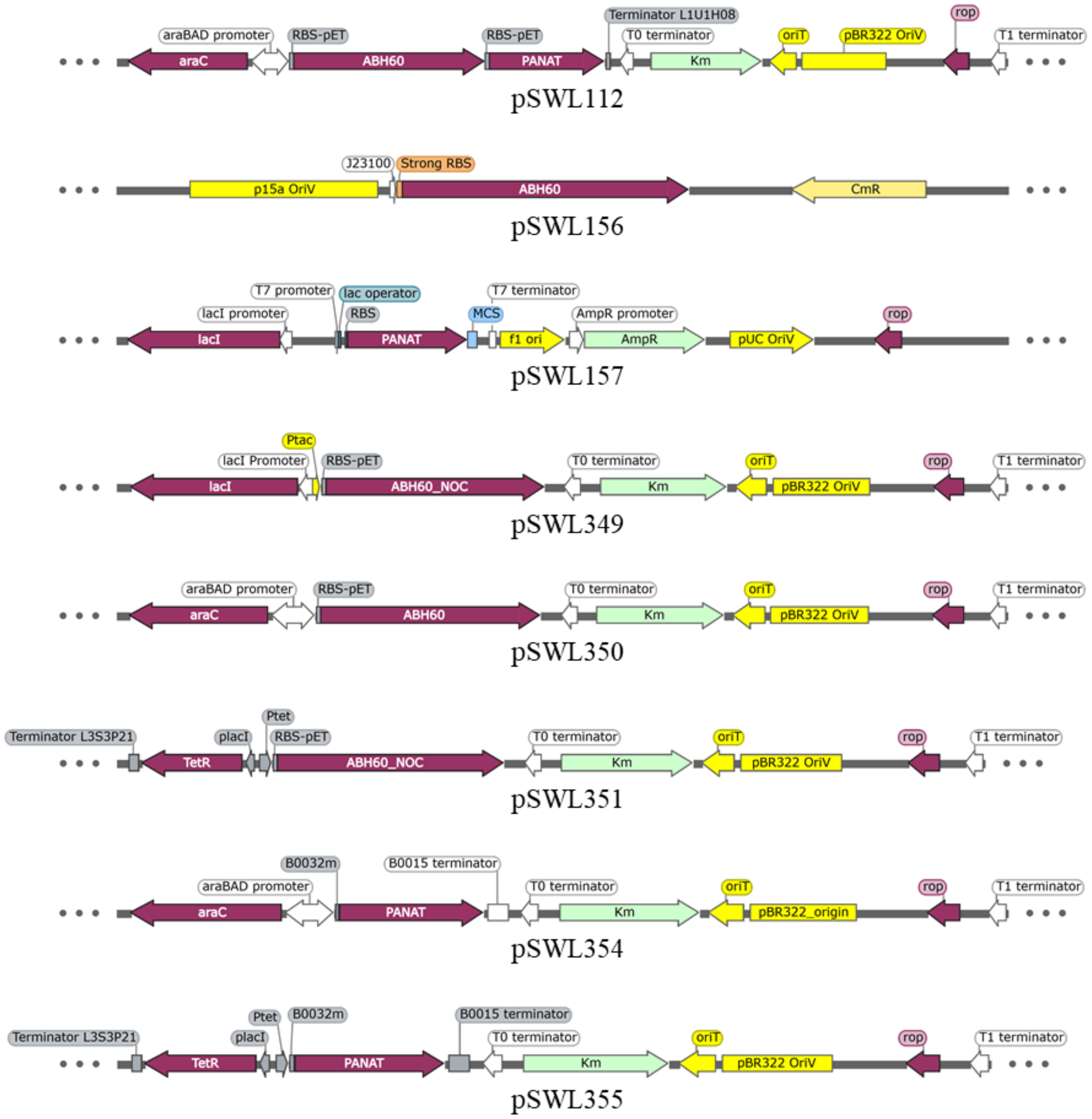

Figure S9. Plasmid maps of constructs used in this study.

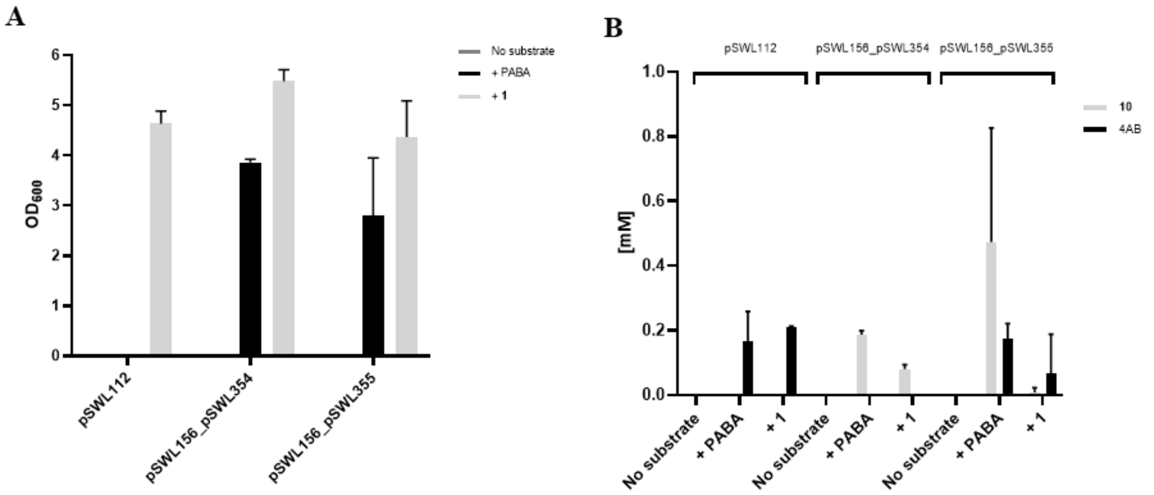

Figure S10. Biosynthesis of **10** from **1** (0.5 mM) or PABA (0.5 mM) using Lossen-dependent growth of strains *E. coli* BW25113Δ*pabB* co-expressing ABH60 and PANAT. **A** Cell density of the cultures measured at 76 h **B** Concentration of **10** and 4-AB at 76 h. Time of induction for pSWL112: 24 h, and for pSWL156_pSWL354 and pSWL156_pSWL355: 72 h. All data are shown as triplicate experiments to one standard deviation.

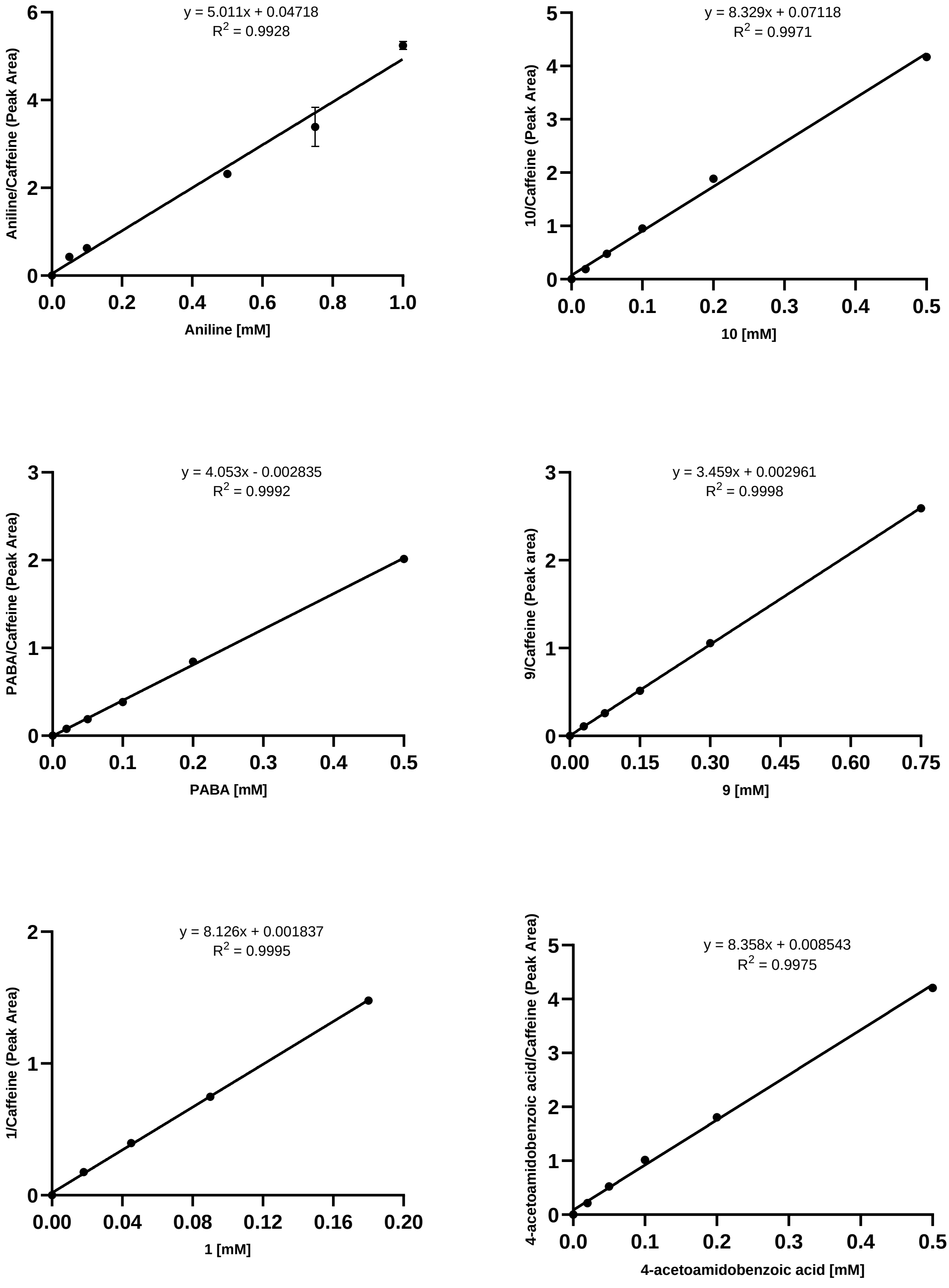

Figure S11. HPLC calibration curves for aniline (Method A) and for **1**, **9**, **10**, and 4-acetoamidobenzoic acid (Method B). At each analyte concentration the mean value is plotted. All data are shown as triplicate experiments to one standard deviation.

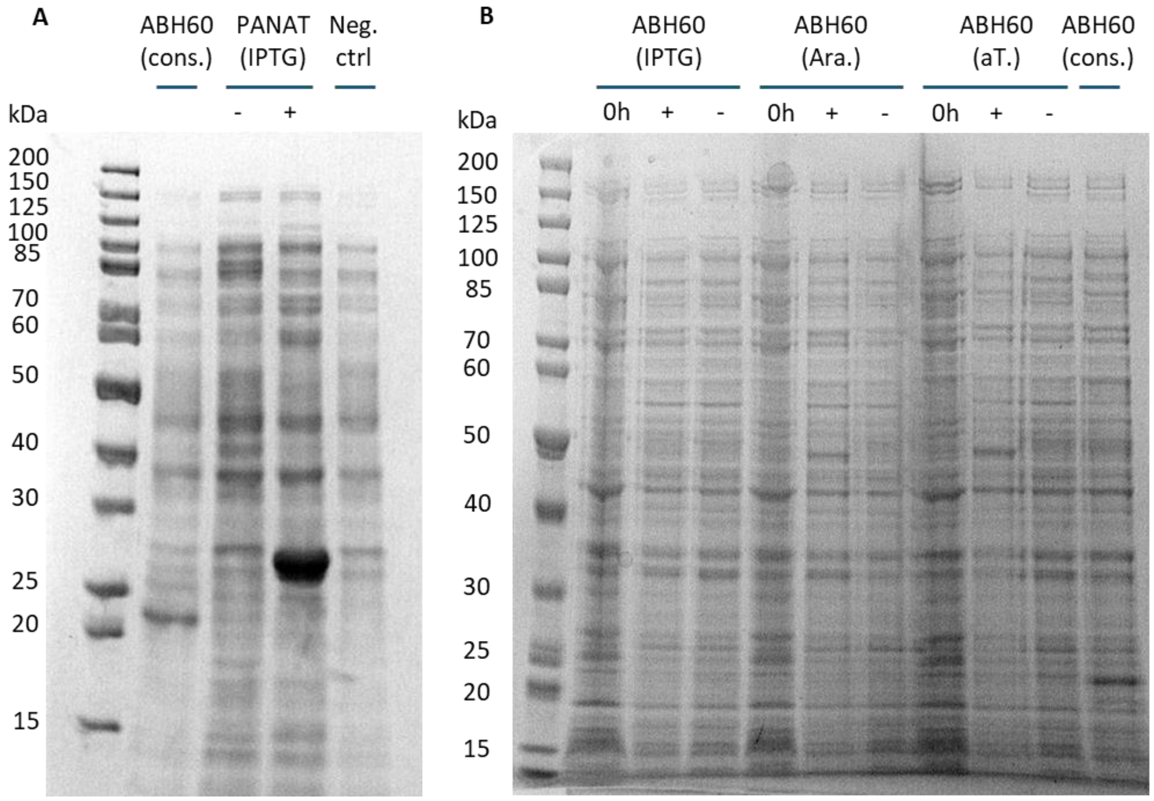

Figure S12. SDS-PAGE gel analysis of expression of ABH or PANAT. Constitutive expression (cons.) ABH60 (from pSWL156), and inducible expression of PANAT with IPTG (pSWL157), and inducible expression of ABH60 with IPTG (pSWL349), arabinose (ara., pSWL350), and anhydrotetracycline (aT., pSWL351) displaying: pre-induction samples (0h), induced for 20 h (+) or non-induced for 20 h (-). BW25113Δ*pabB* was used with all plasmids (without plasmid for the negative control) except for pSWL157, which was expressed using BL21(DE3). Expected masses: ABH (53 kDa), PANAT (28 kDa).

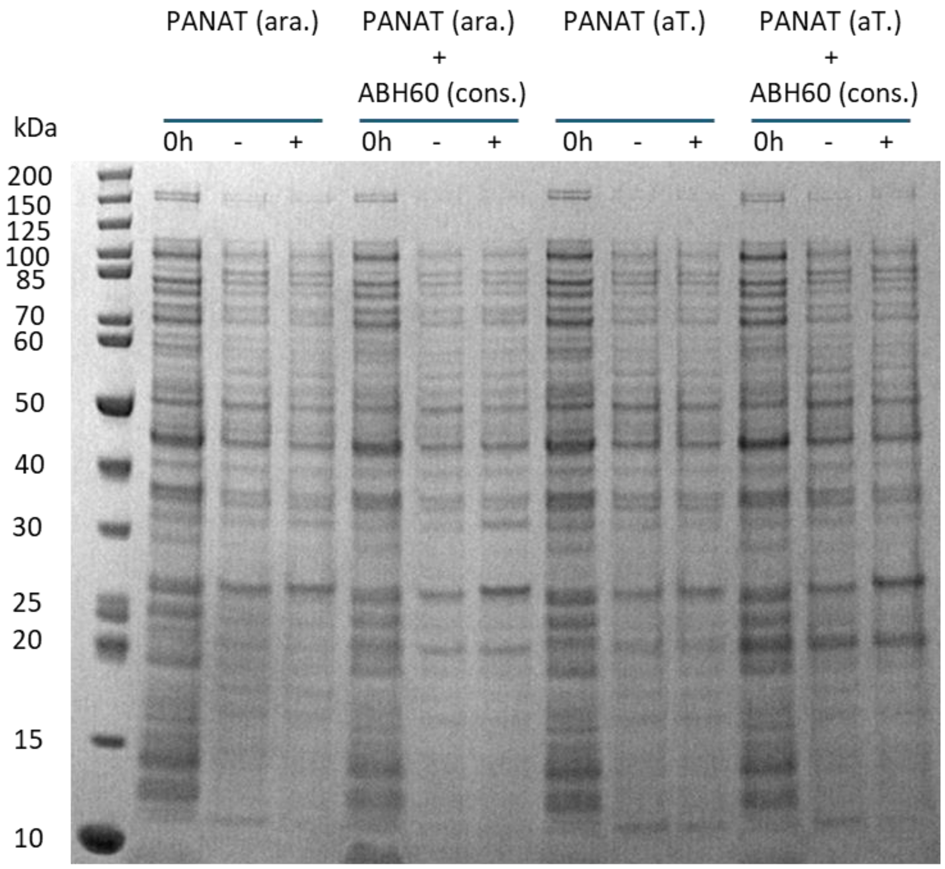

Figure S13. SDS-PAGE gel analysis of co-expression of ABH or PANAT. *E. coli* BW25113Δ*pabB* expressing PANAT with ararbionse-inducible plasmid pSWL354 (ara.) or the anhydrotetracycline-inducible pSWL355 (aT.) and, if indicated, co-expressing ABH60 from constitutive from pSWL156. Displaying: pre-induction samples (0h), induced (+) for 20 h or non-induced (-) for 20 h. Expected masses: ABH (53 kDa), PANAT (28 kDa).

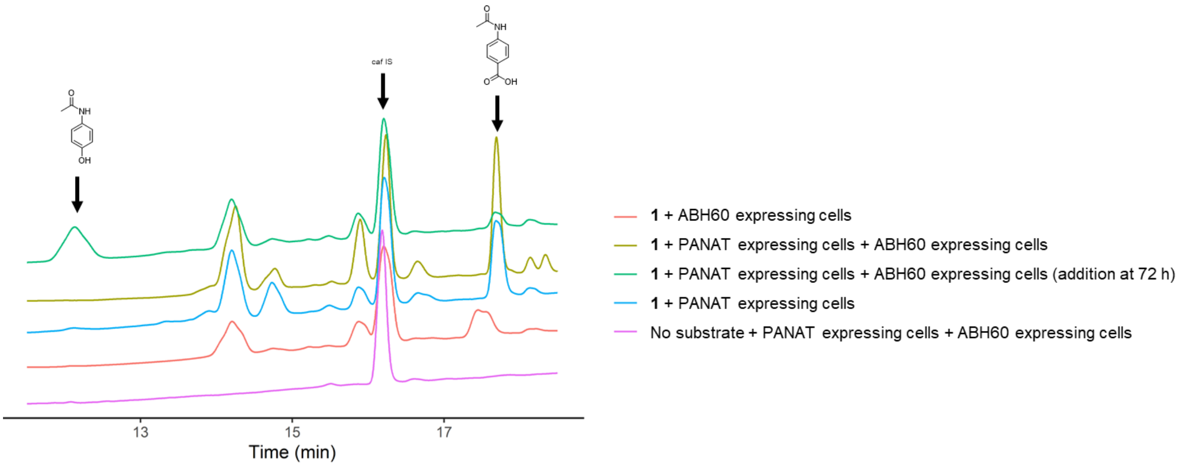

Figure S14. Aligned HPLC chromatographs of **10** biosynthesis from **1** using combinations of Lossen-dependent growth of *E. coli* BW25113Δ*pabB* expressing ABH60 and *E. coli* BL21(DE3)_pSWL157 expressing PANAT.

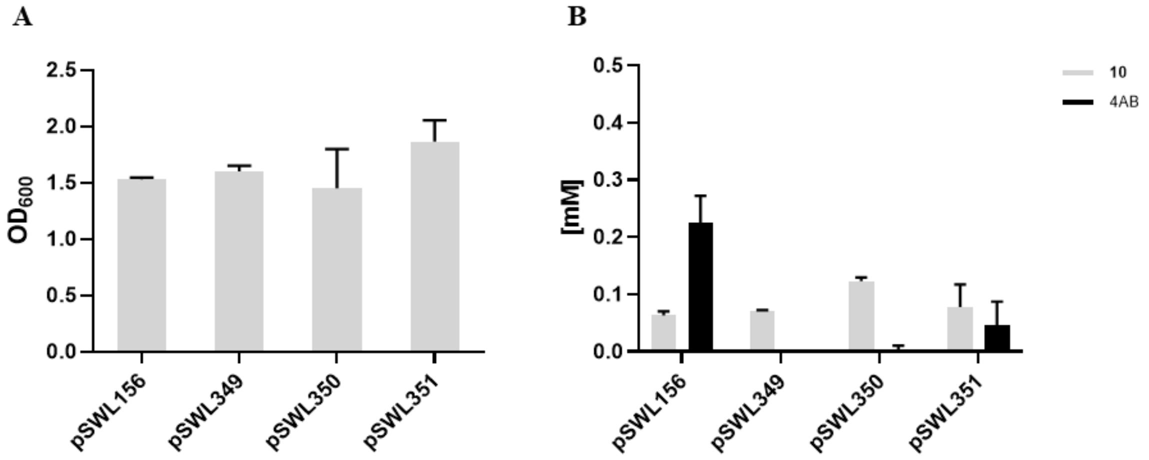

Figure S15. Biosynthesis of **10** from **1** (0.42 mM) using Lossen-dependent growth of *E. coli* BW25113Δ*pabB* expressing ABH60 and then addition of *E. coli* BL21(DE3)_pSWL157 expressing PANAT. **A** Cell density of *E. coli* BW25113Δ*pabB* at 72 h. **B** Concentration of **10** and 4-AB 24 h after addition of PANAT-expressing cells.

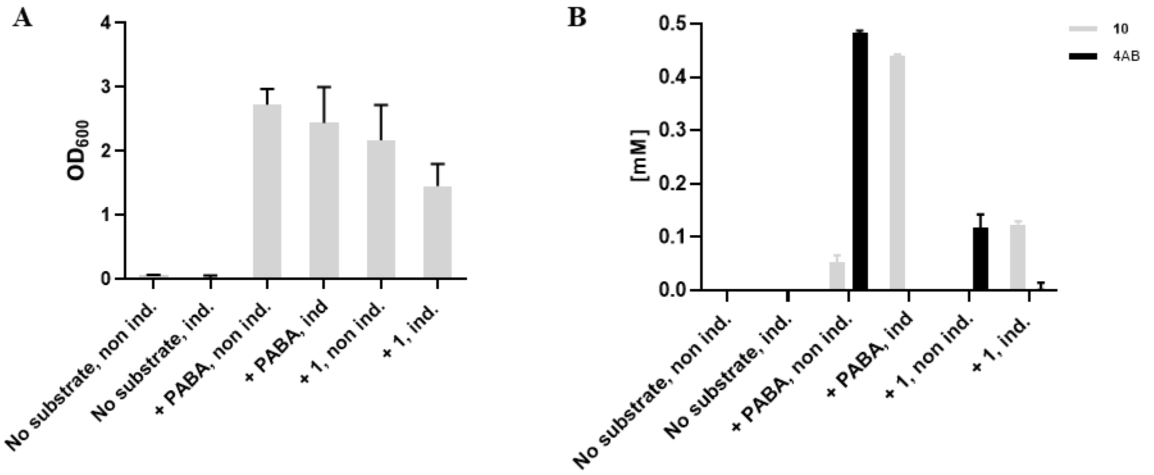

Figure S16. Control reactions of the biosynthesis of **10** from **1** (0.42 mM) or PABA (0.42 mM) using Lossen-dependent growth of strains *E. coli* BW25113Δ*pabB*_pSWL350 and then addition of *E. coli* BL21(DE3)_pSWL157 expressing PANAT at 72 h. **A** Cell density of *E. coli* BW25113Δ*pabB* at 72 h, induction at 24 h. **B** Concentration of **10** and 4AB at 96 h. All data are shown as triplicate experiments to one standard deviation.

S8. NMR Spectroscopy

^{1}H NMR spectrum of **1** in DMSO-d_6

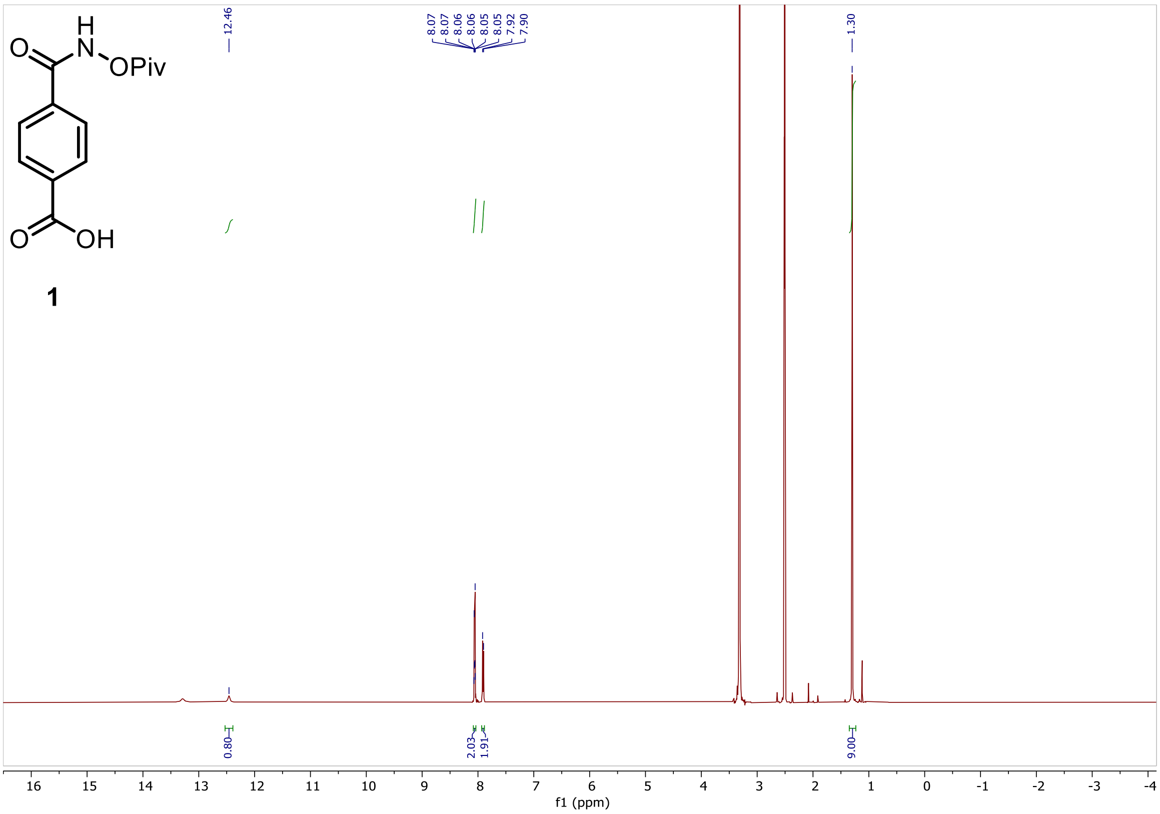

^{13}C NMR spectrum of **1** in DMSO-d_6

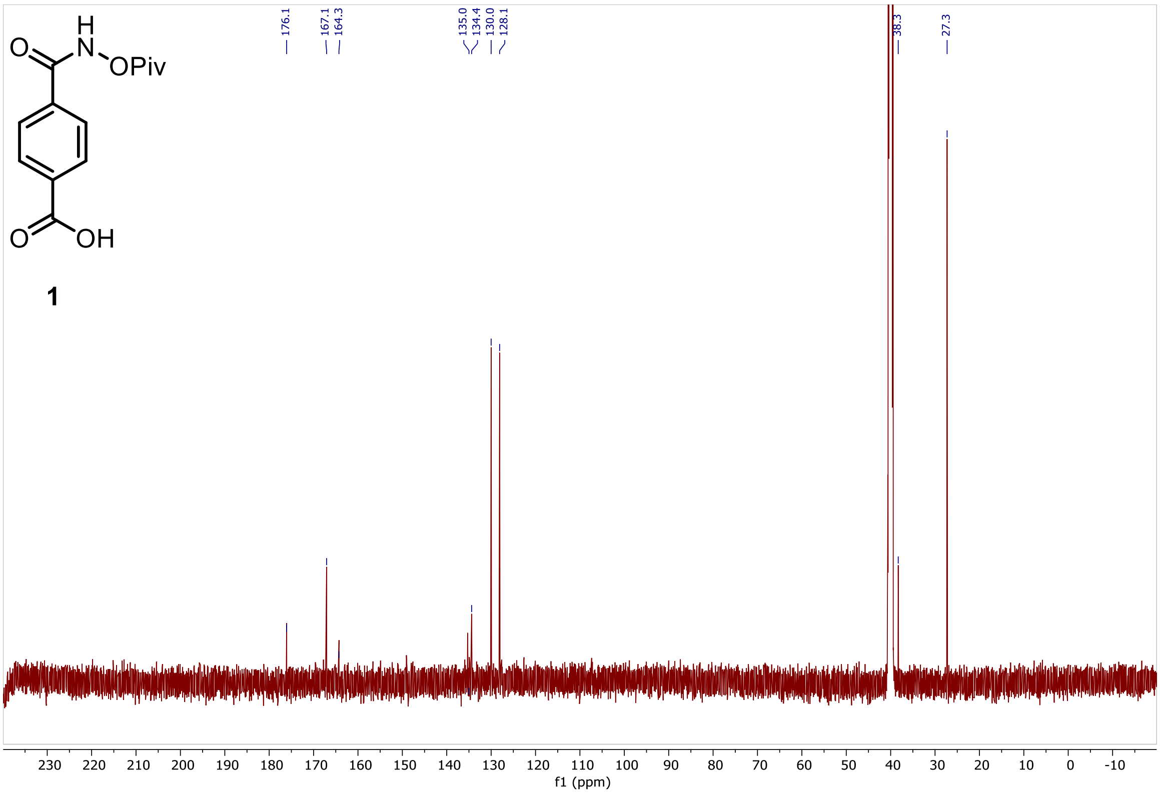

^{1}H NMR spectrum of **2** in DMSO-d_6.

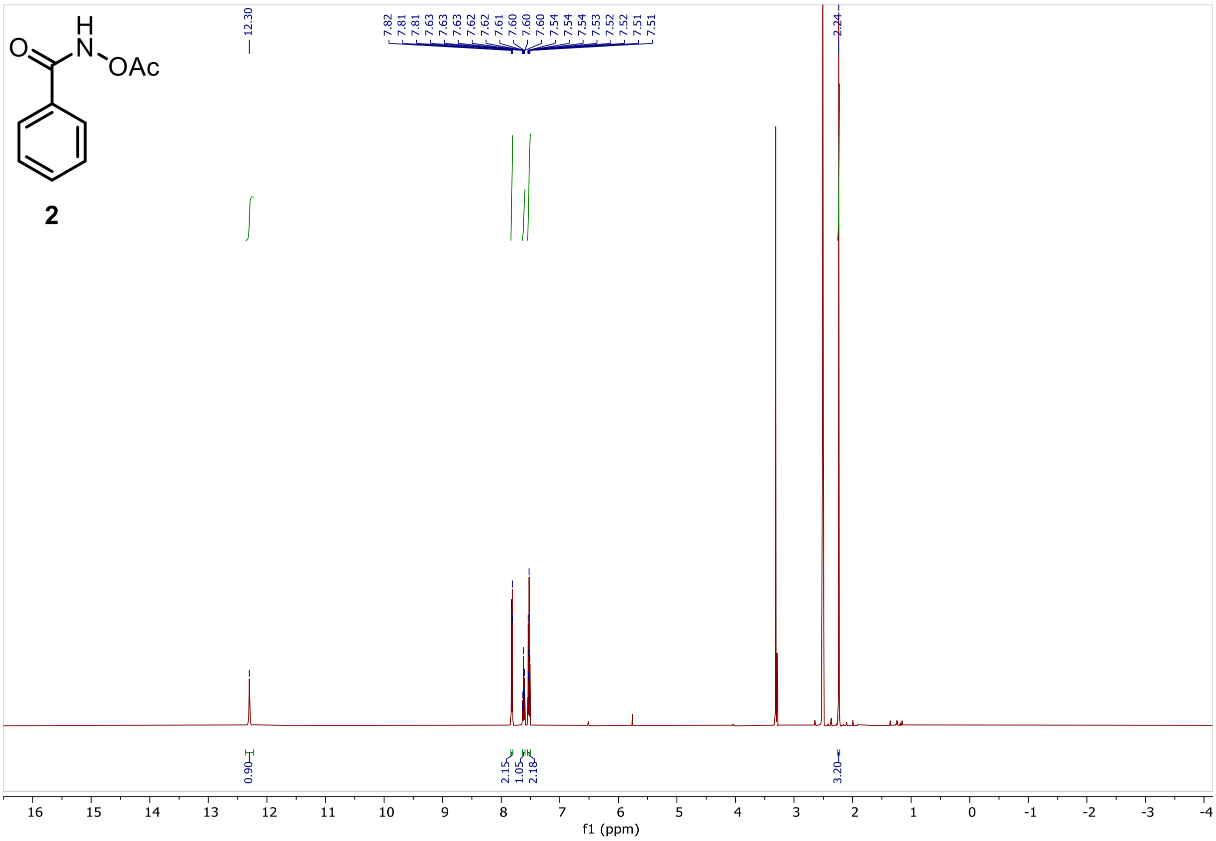

^{13}C NMR spectrum of **2** in DMSO-d_6.

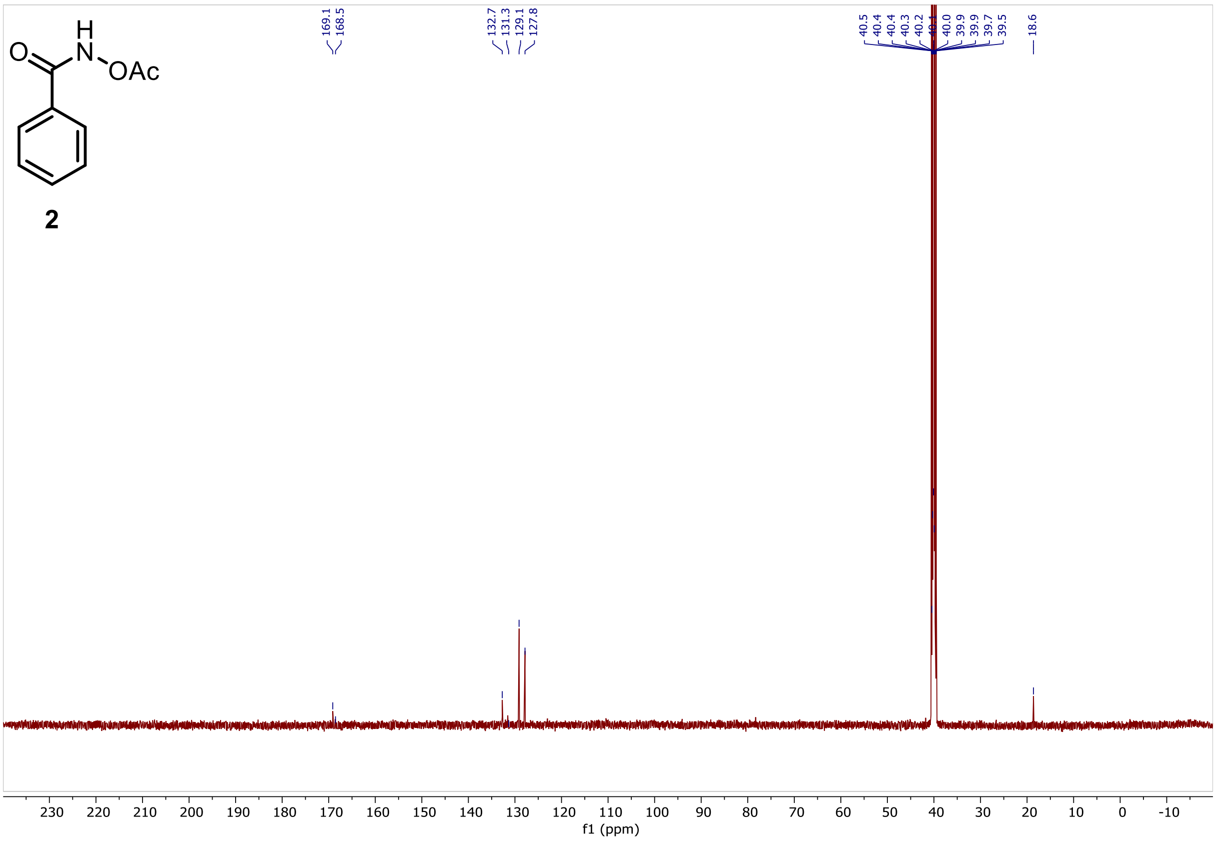

^{1}H NMR spectrum of **3** in MeOD-d$_4$

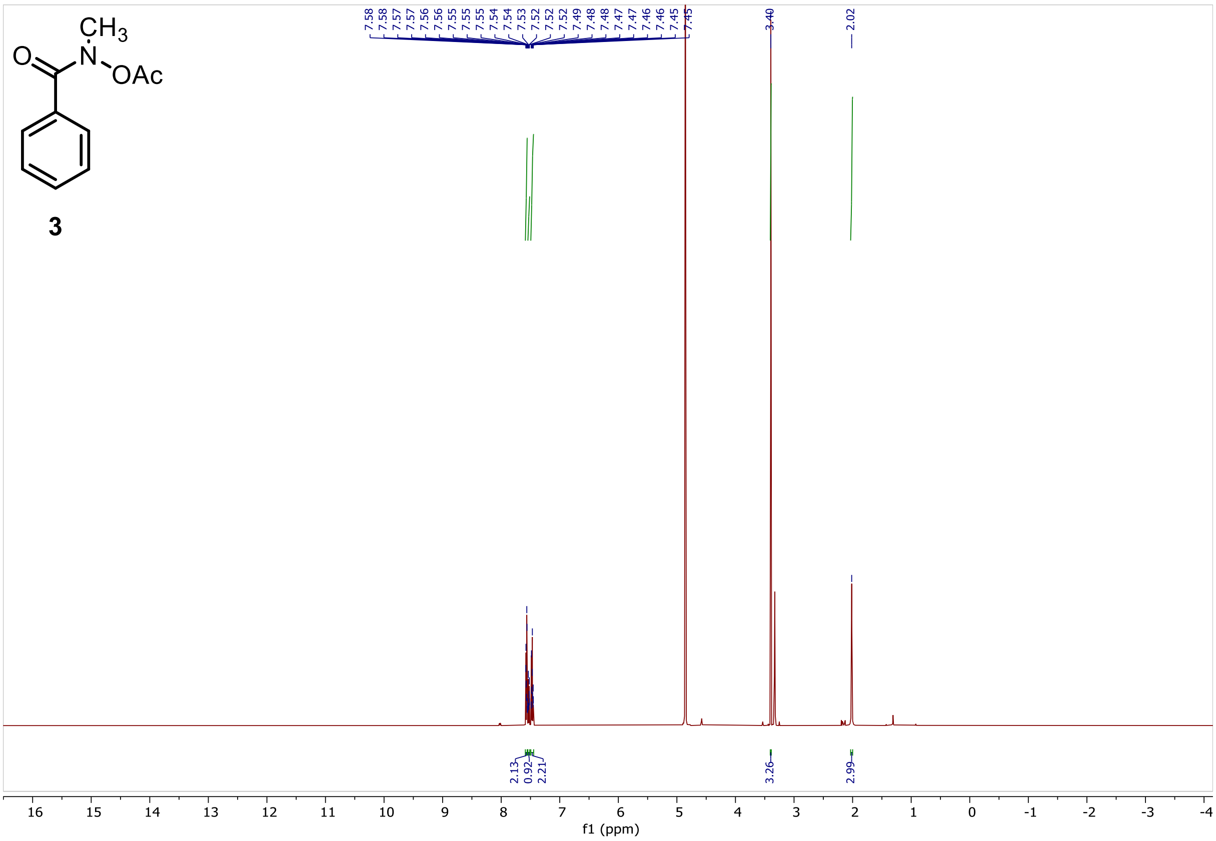

^{13}C NMR spectrum of **3** in MeOD-d$_4$

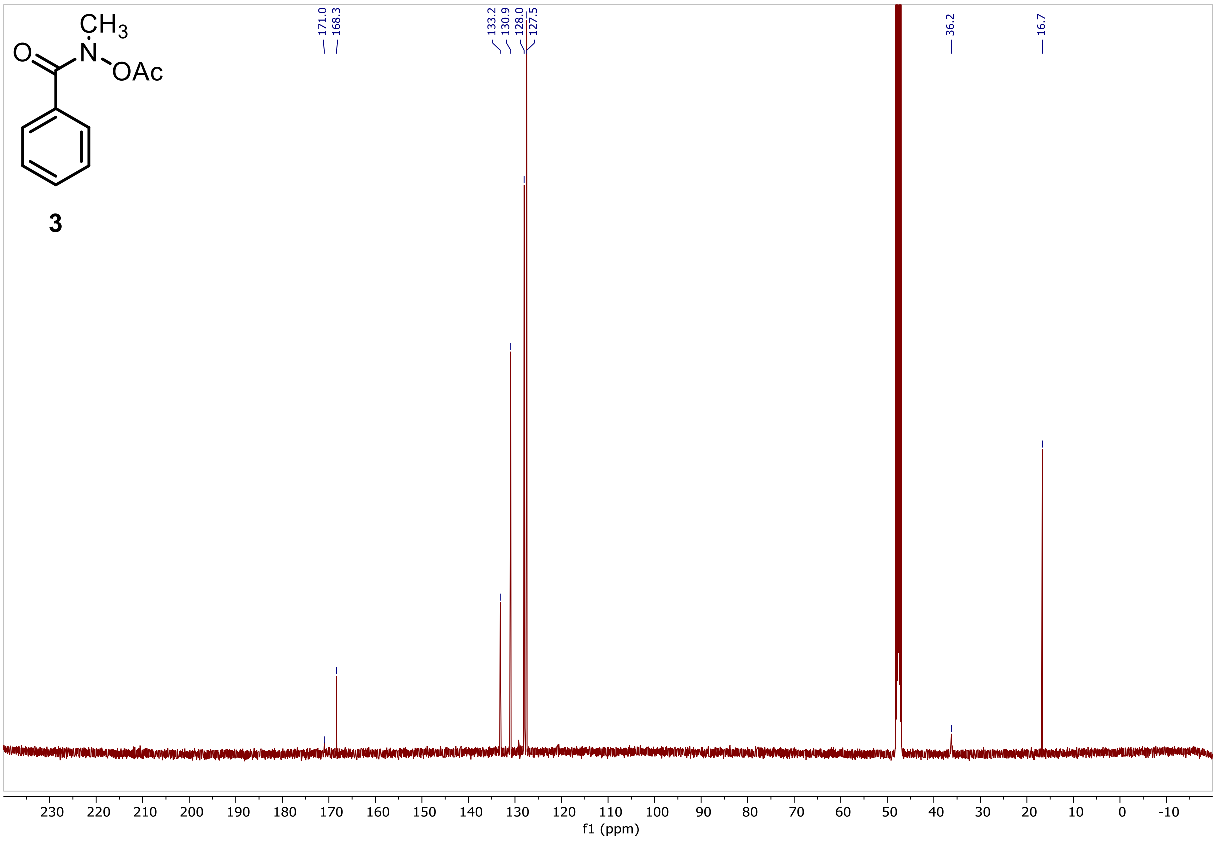

^{1}H NMR spectrum of **S1** in DMSO-d_6.

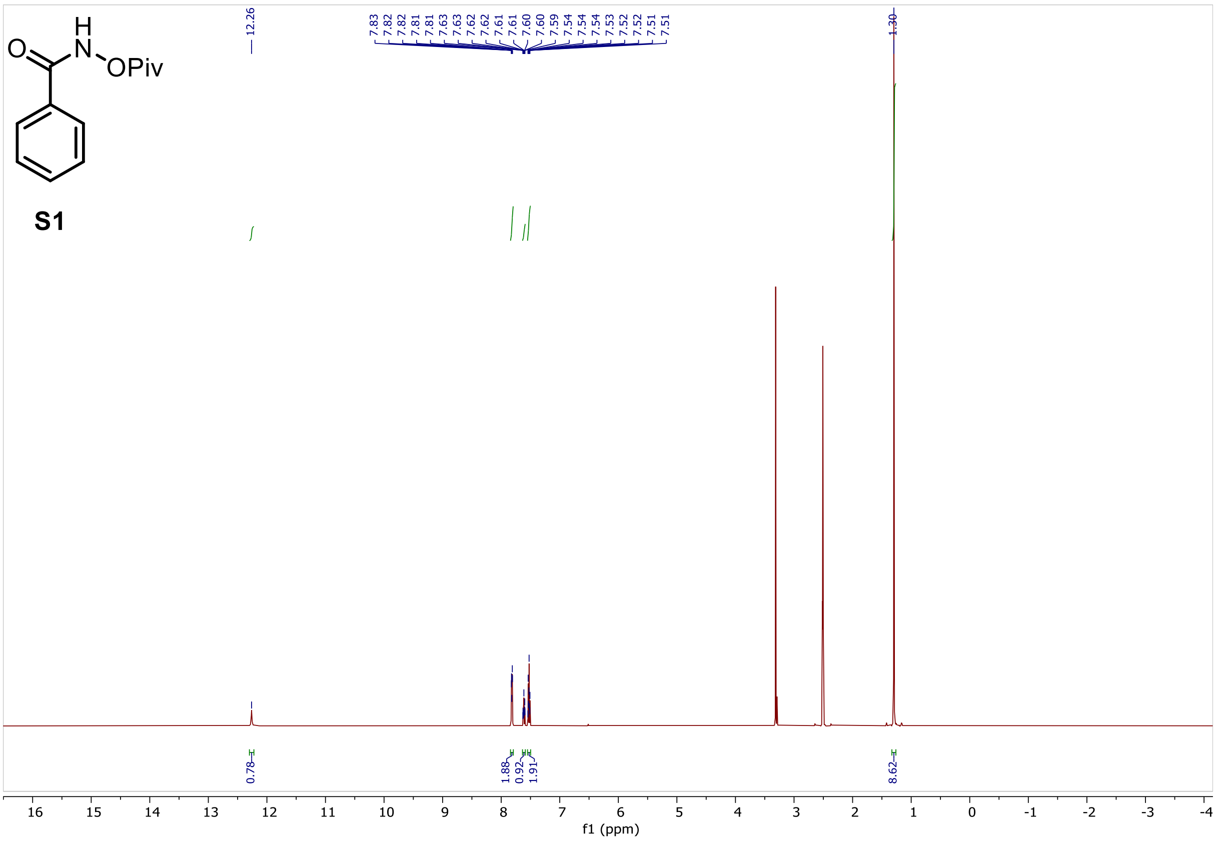

^{13}C NMR spectrum of **S1** in DMSO-d_6.

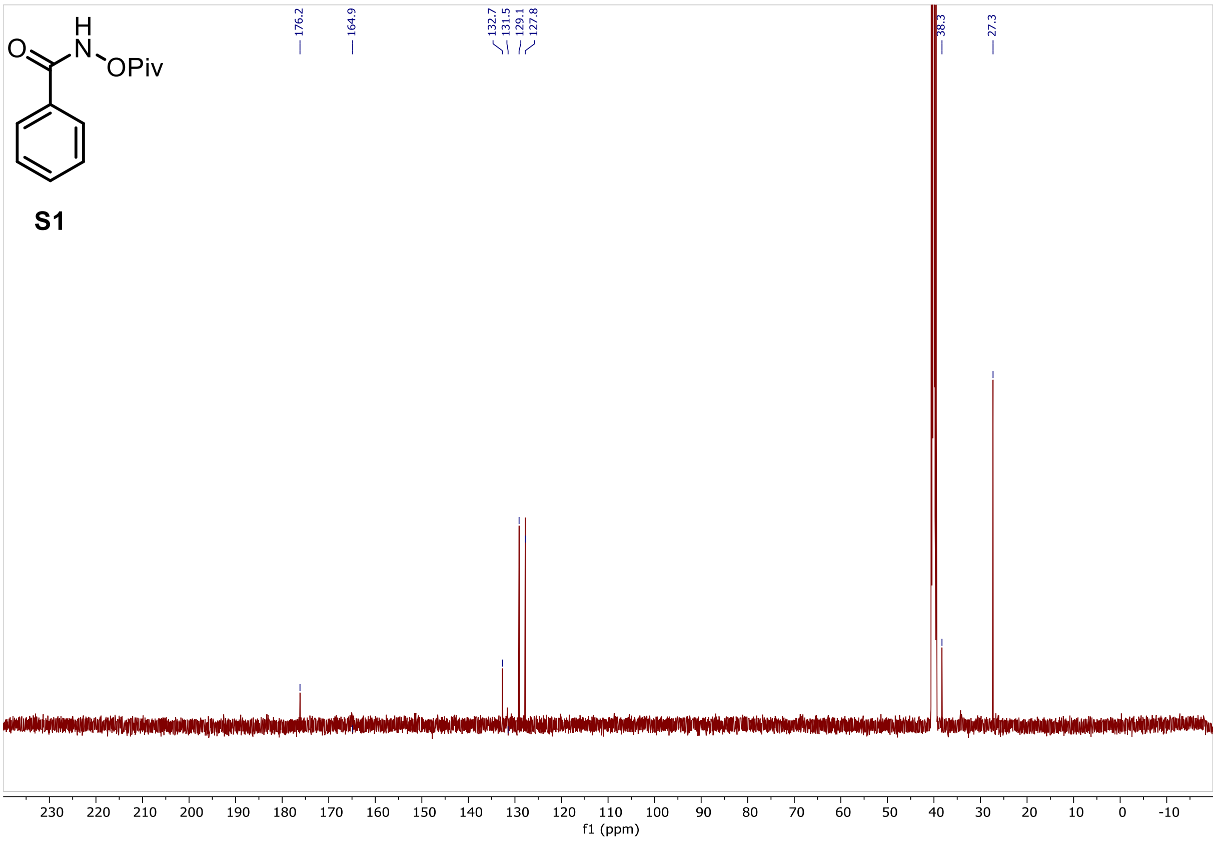

^{1}H NMR spectrum of **S2** in DMSO-d_6.

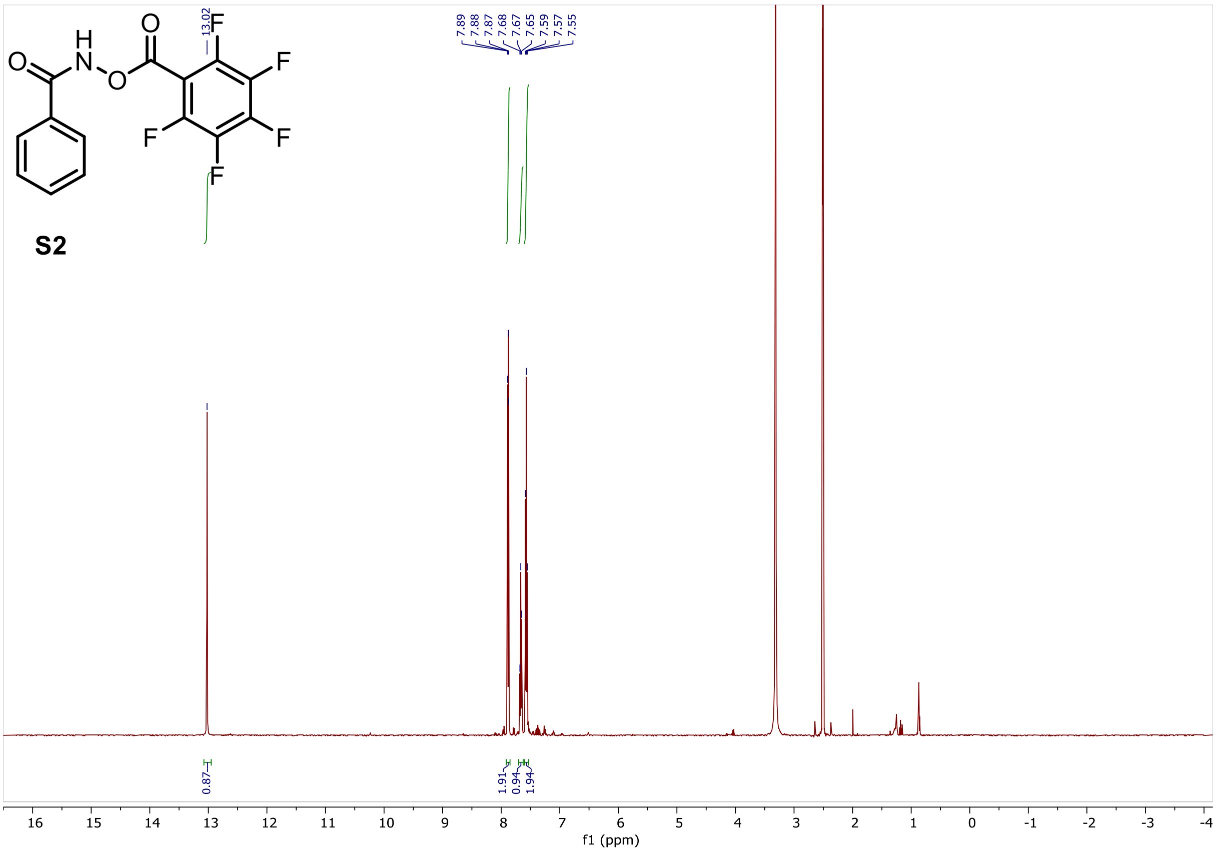

^{13}C NMR spectrum of **S2** in DMSO-d_6.

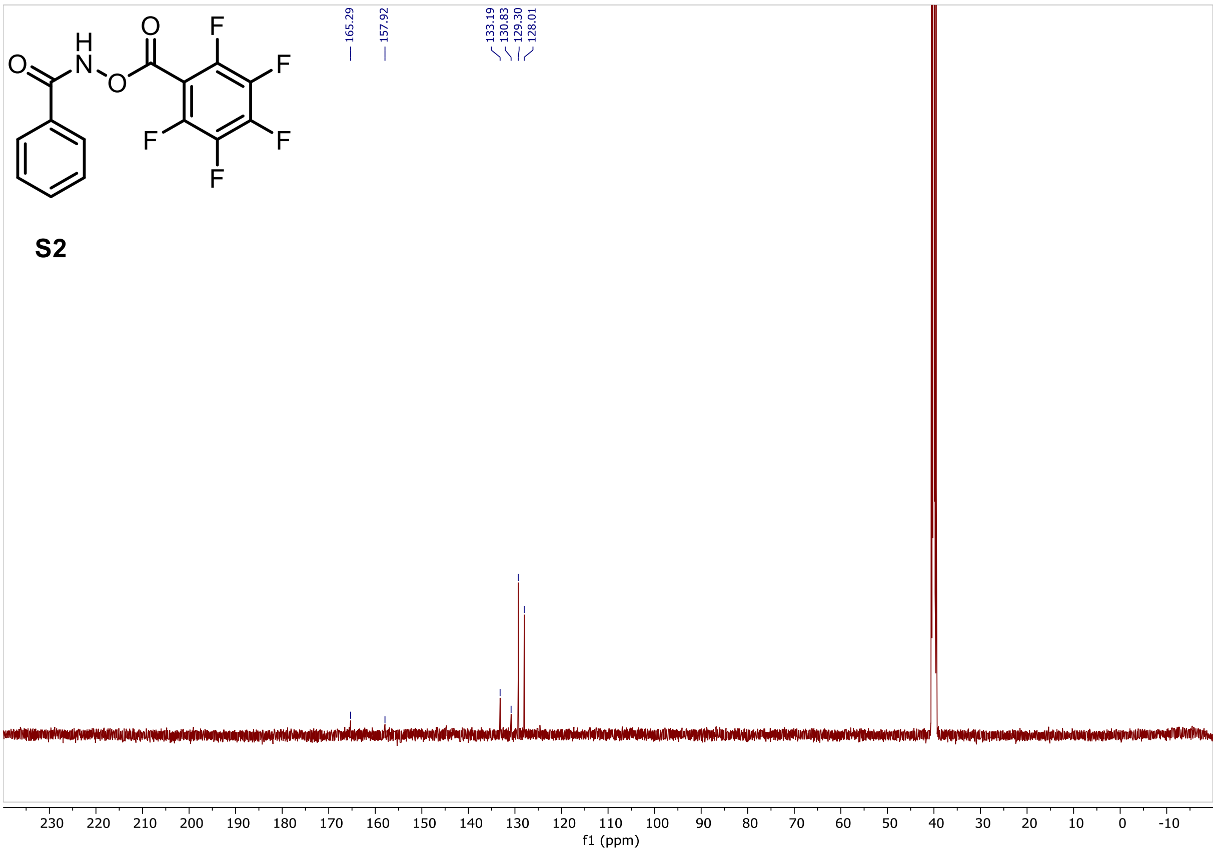

^{1}H NMR spectrum of **S3** in DMSO-d_6.

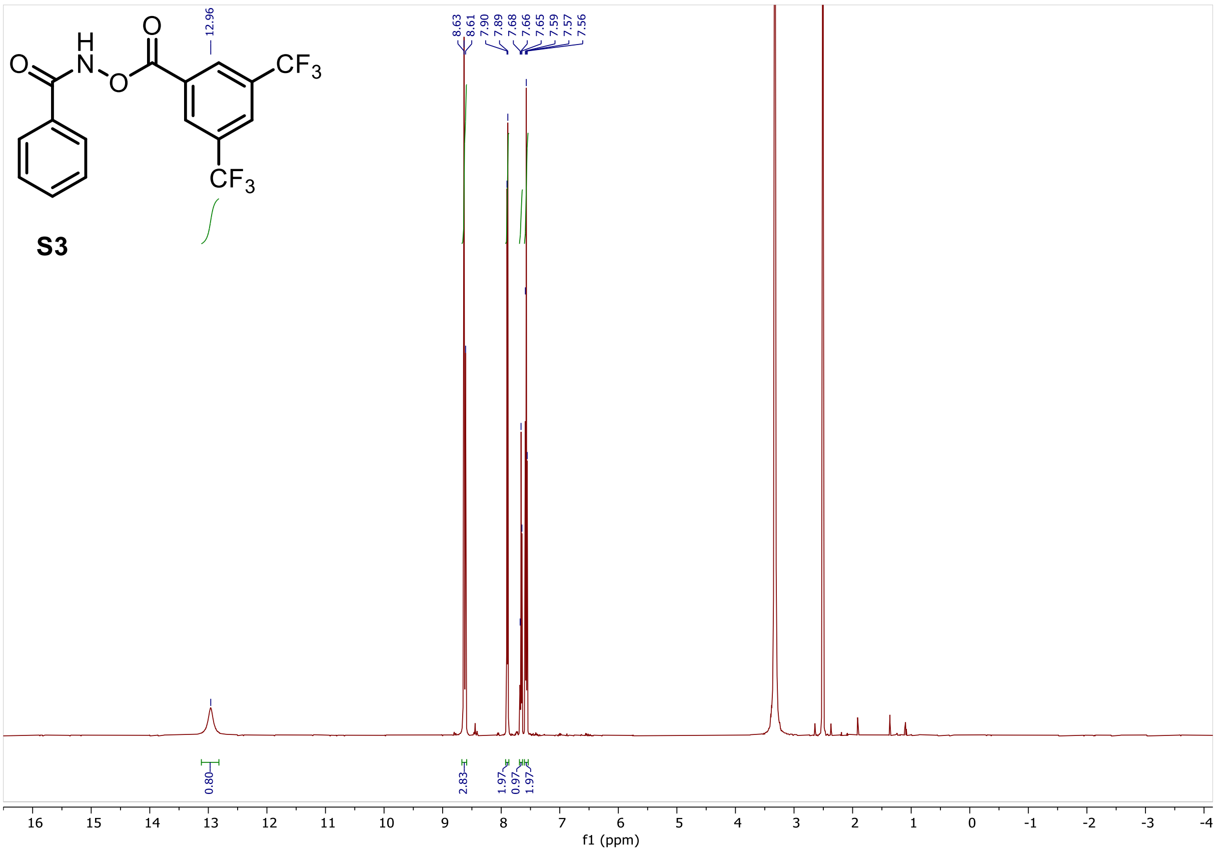

^{13}C NMR spectrum of **S3** in DMSO-d_6.

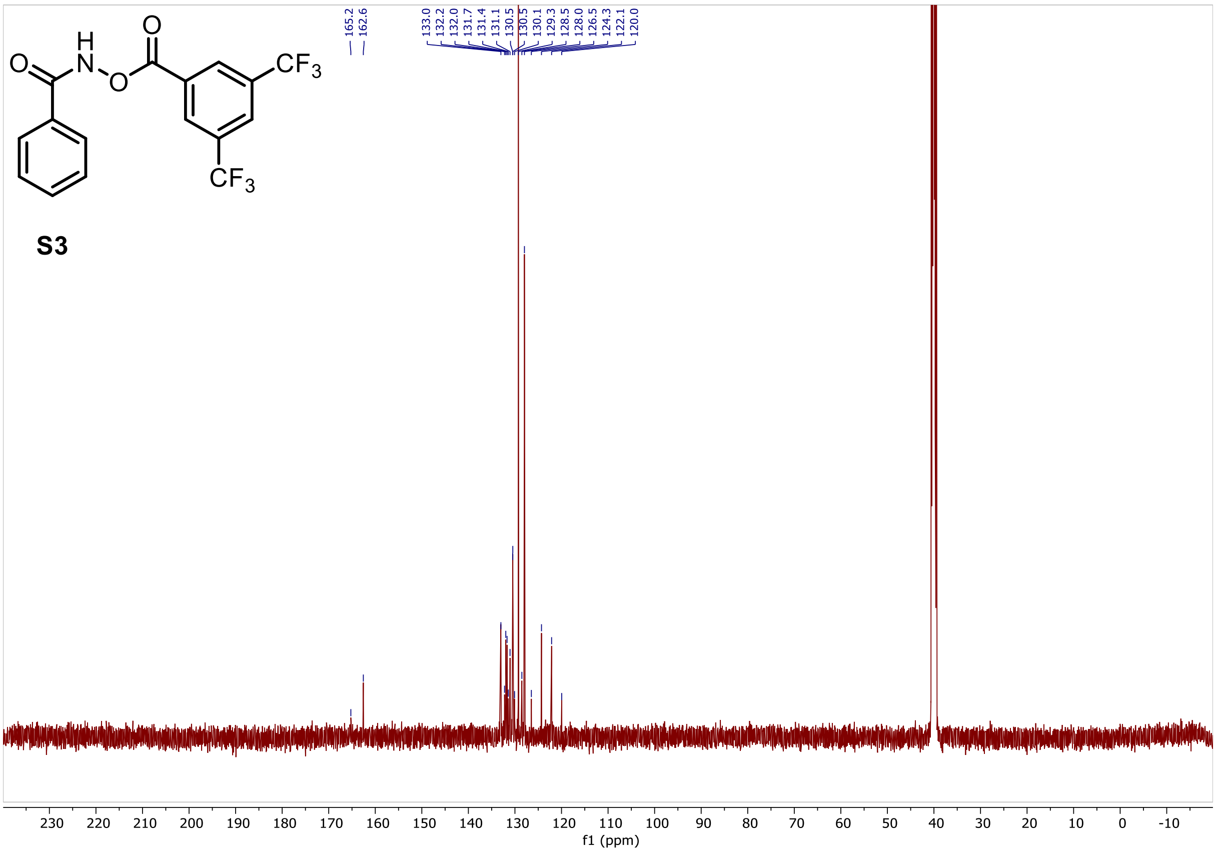

^{1}H NMR spectrum of **S4** in $CDCl_3$

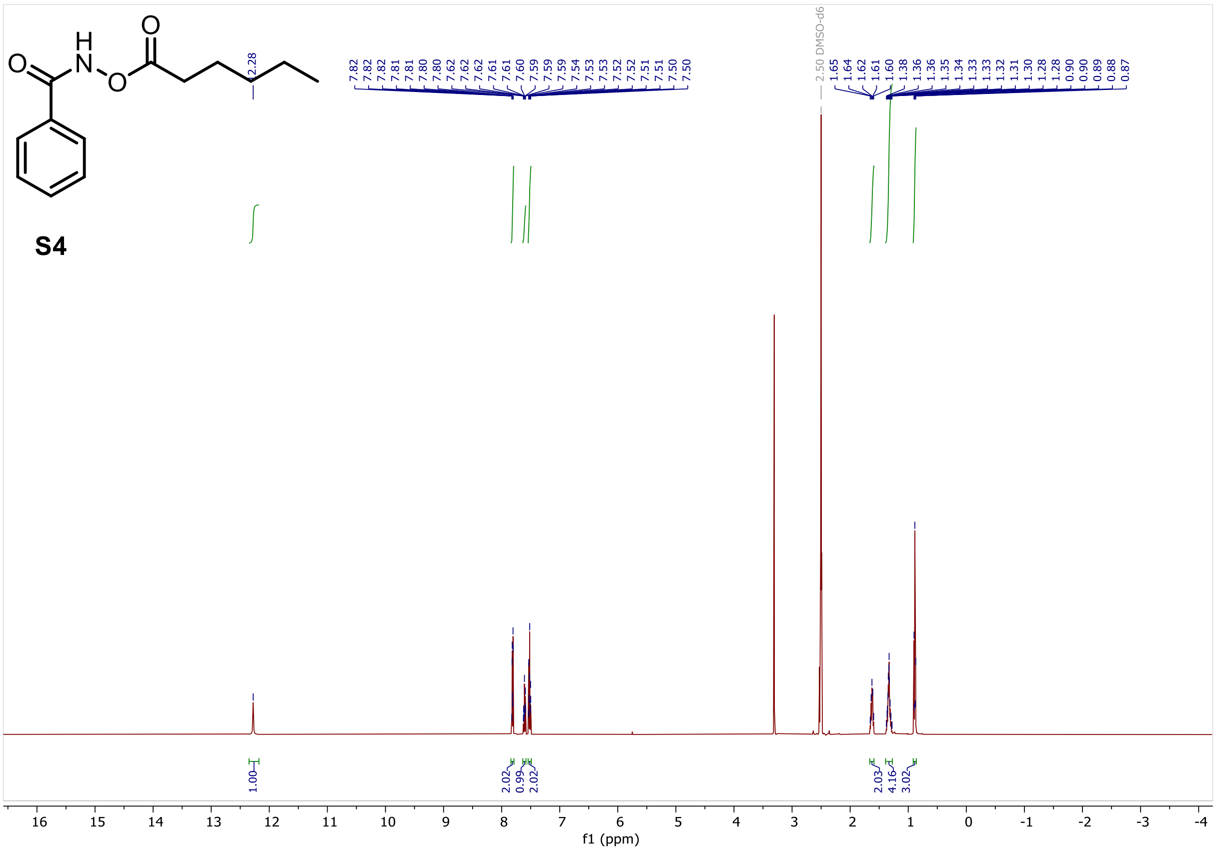

^{13}C NMR spectrum of **S4** in $CDCl_3$

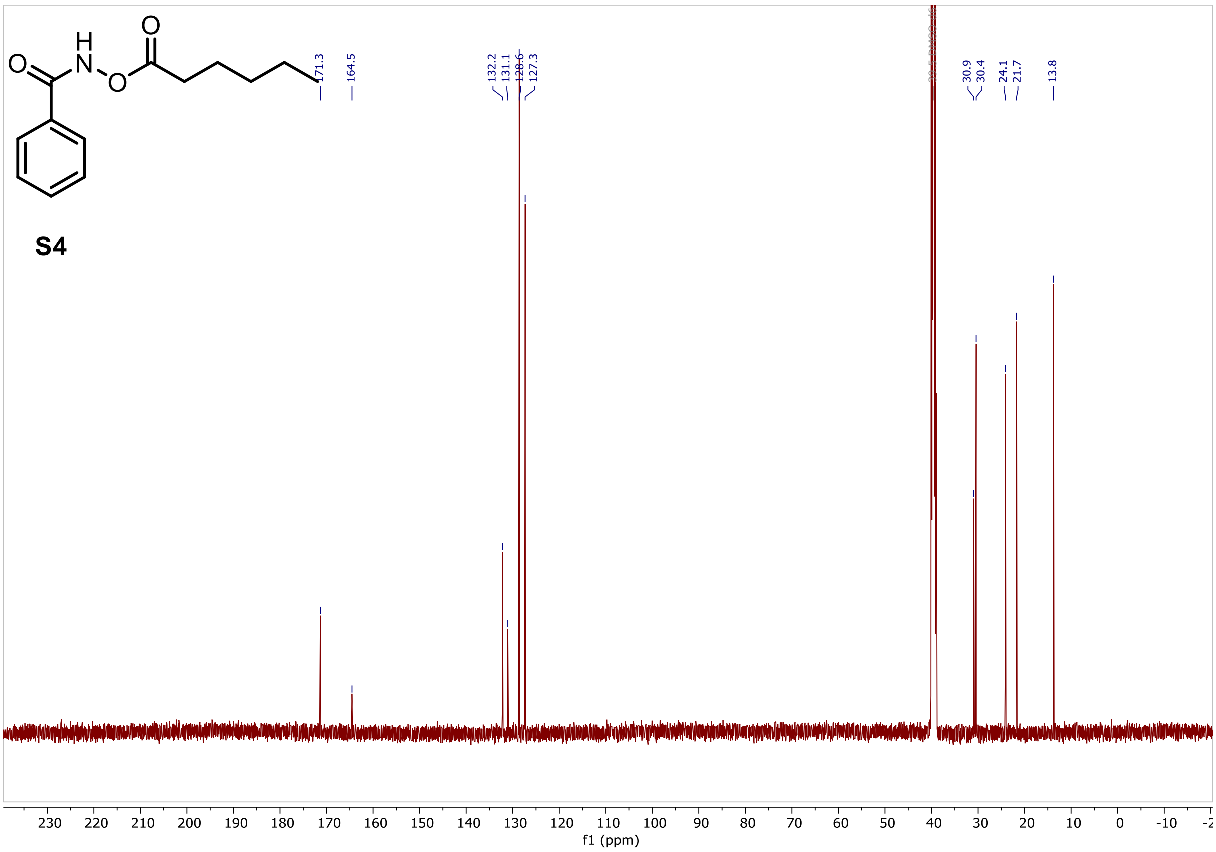

^{1}H NMR spectrum of **S5** in $CDCl_3$

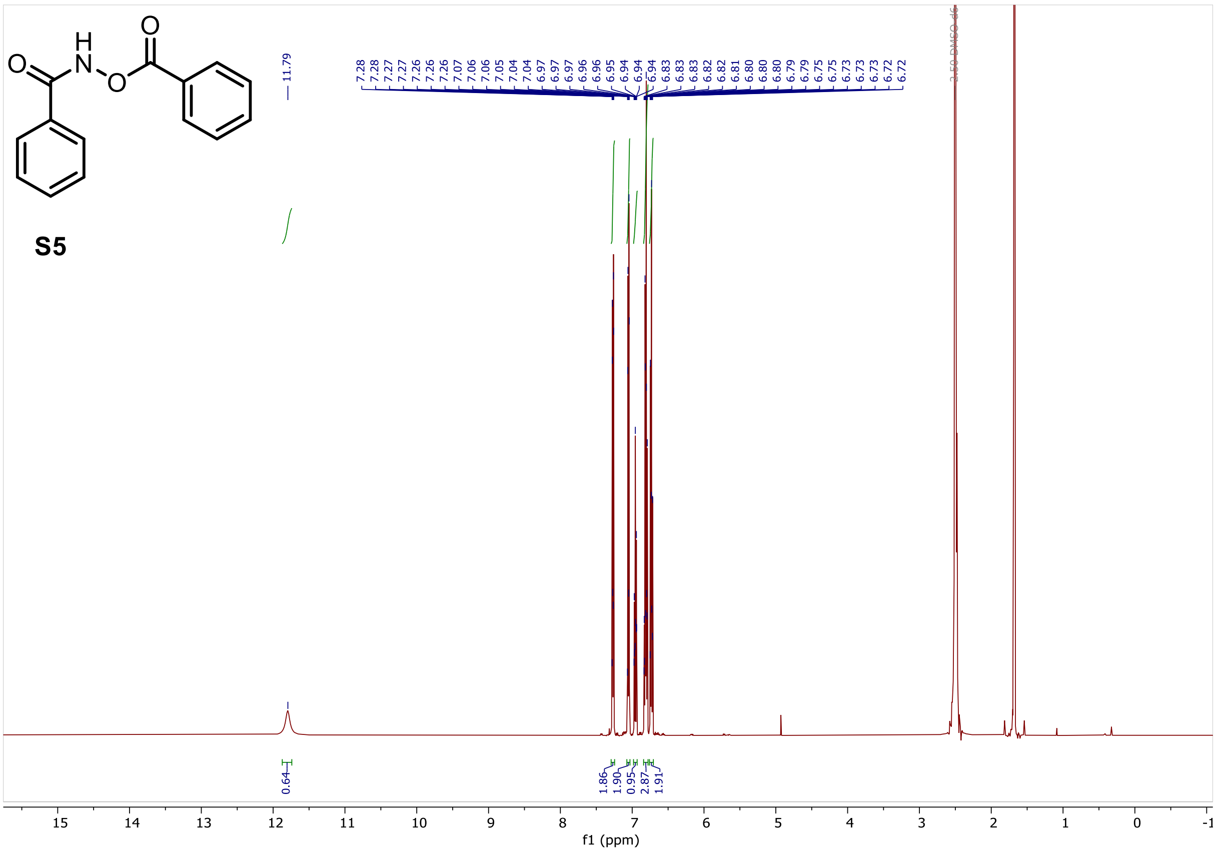

^{13}C NMR spectrum of **S5** in $CDCl_3$

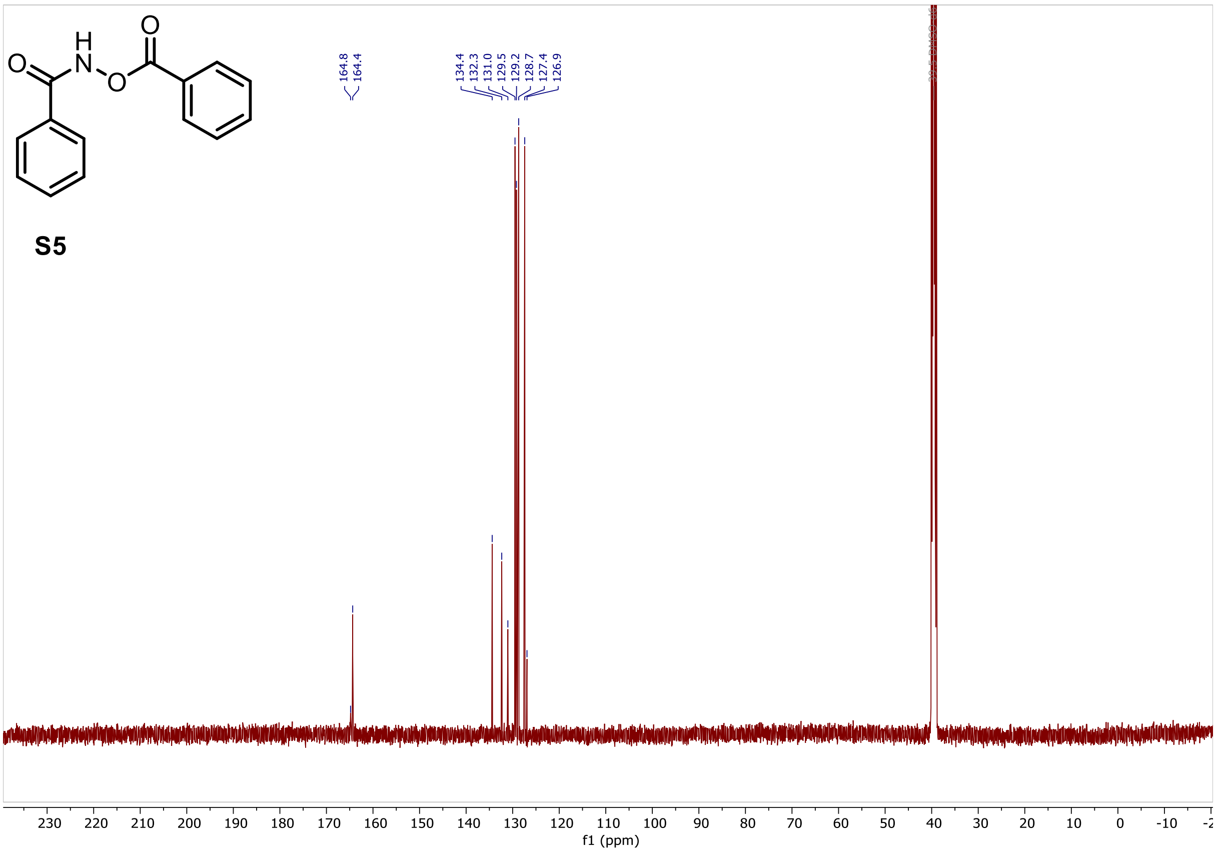

^{1}H NMR spectrum of **S6** in $CDCl_3$

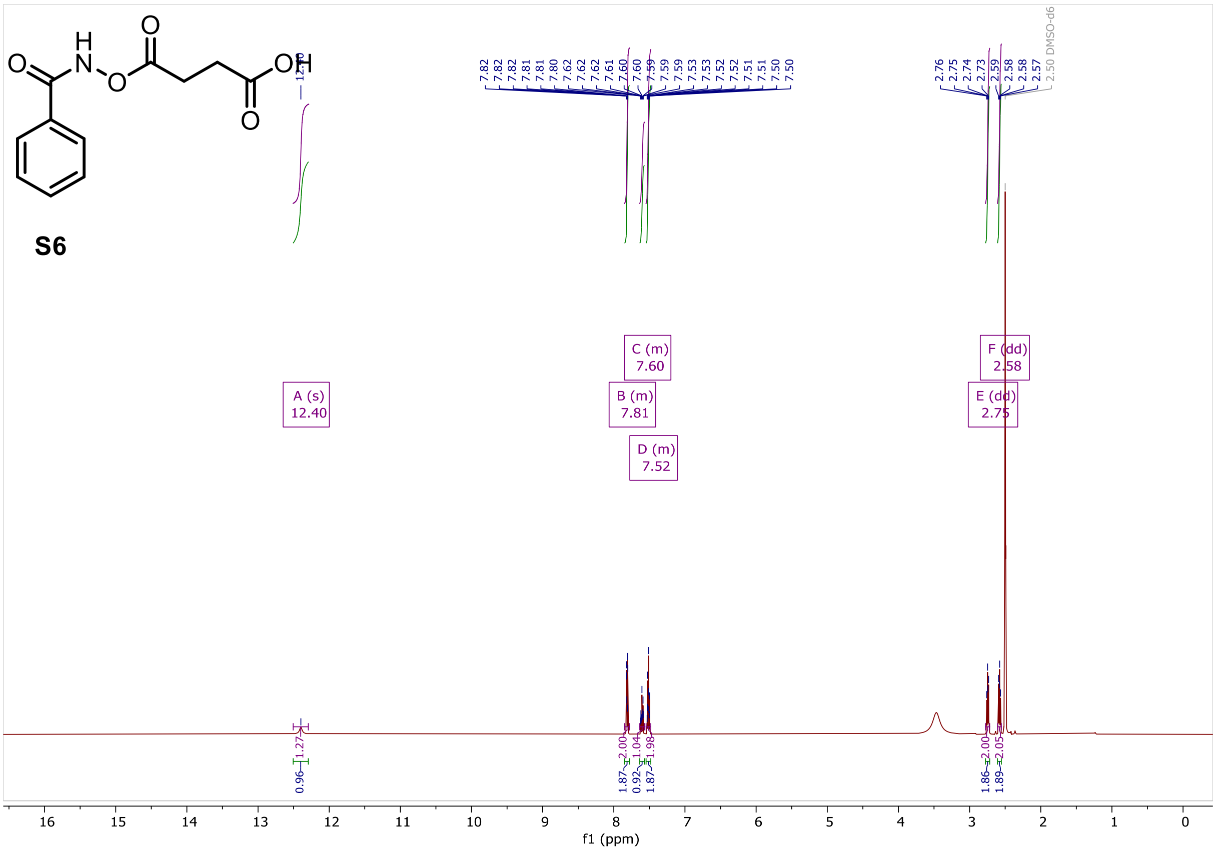

^{13}C NMR spectrum of **S6** in $CDCl_3$

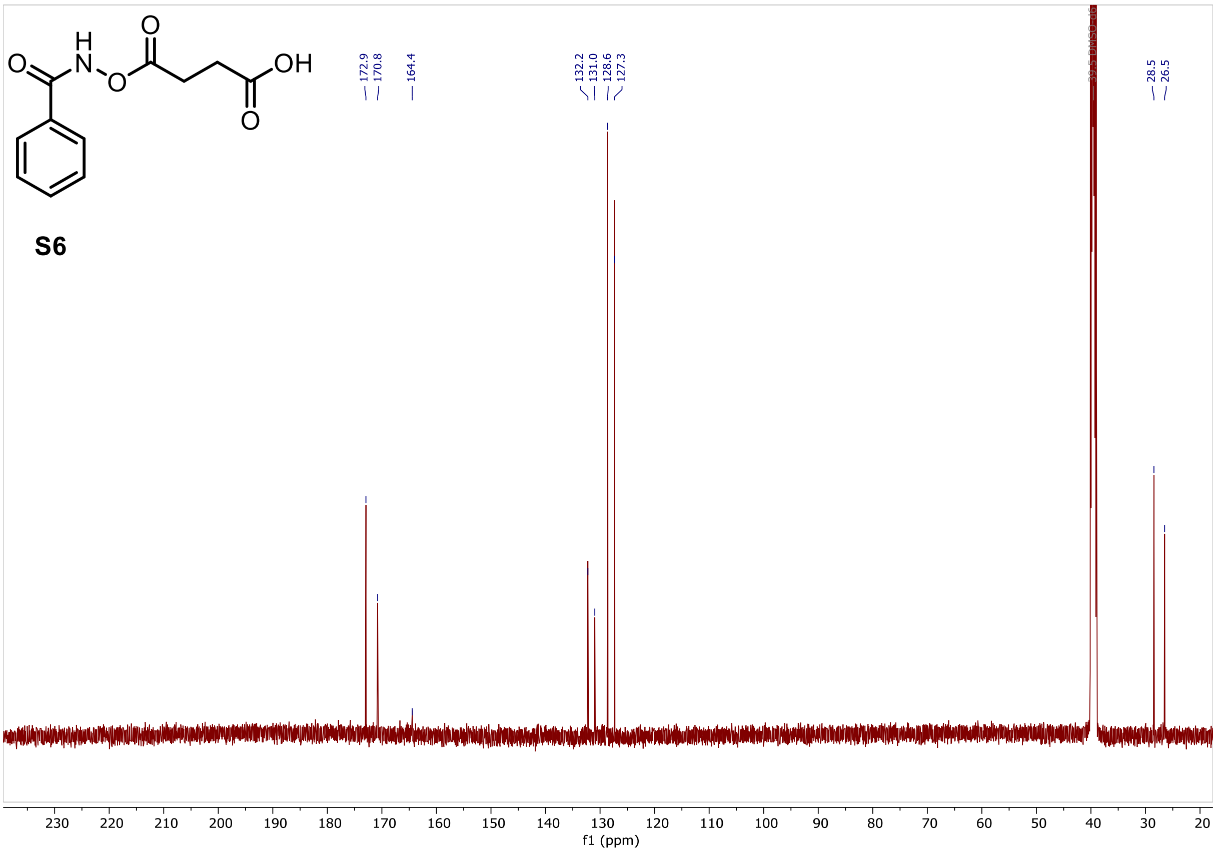

^{1}H NMR spectrum of **PET-S8** in DMSO-d_6

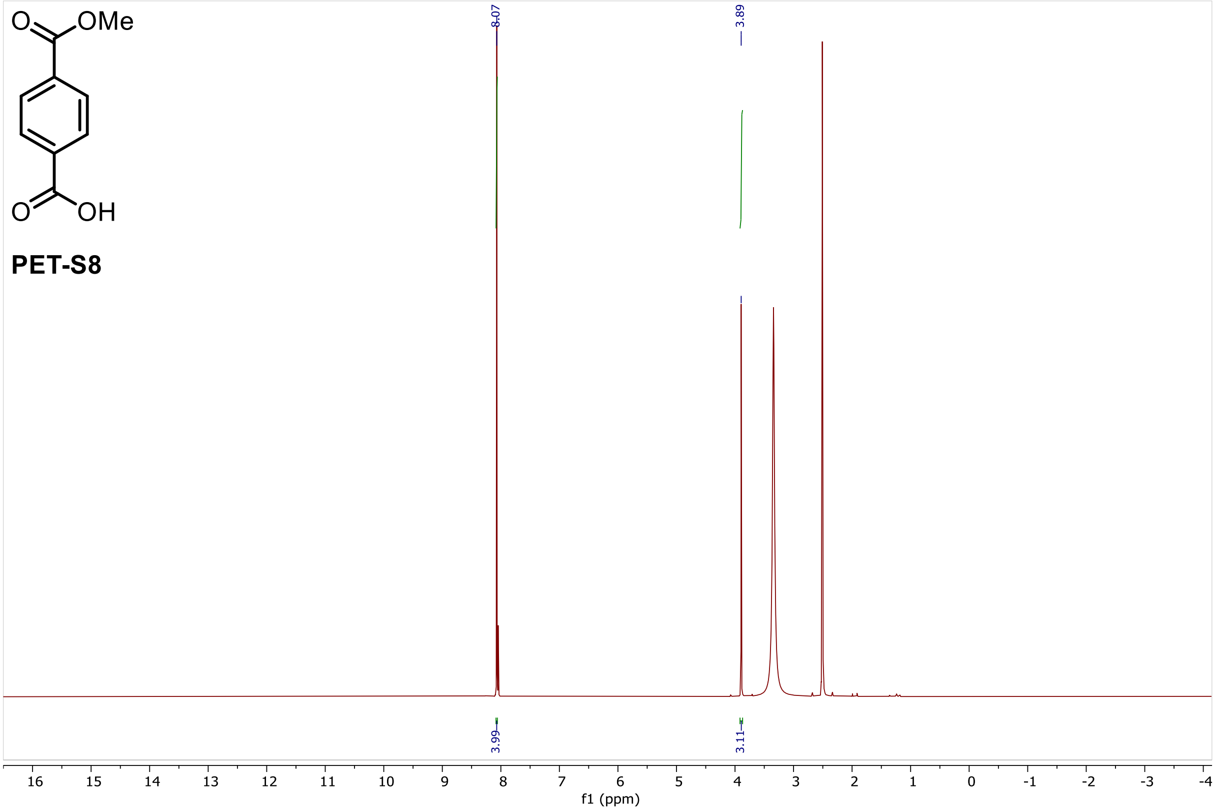

^{13}C NMR spectrum of **PET-S8** in DMSO-d_6.

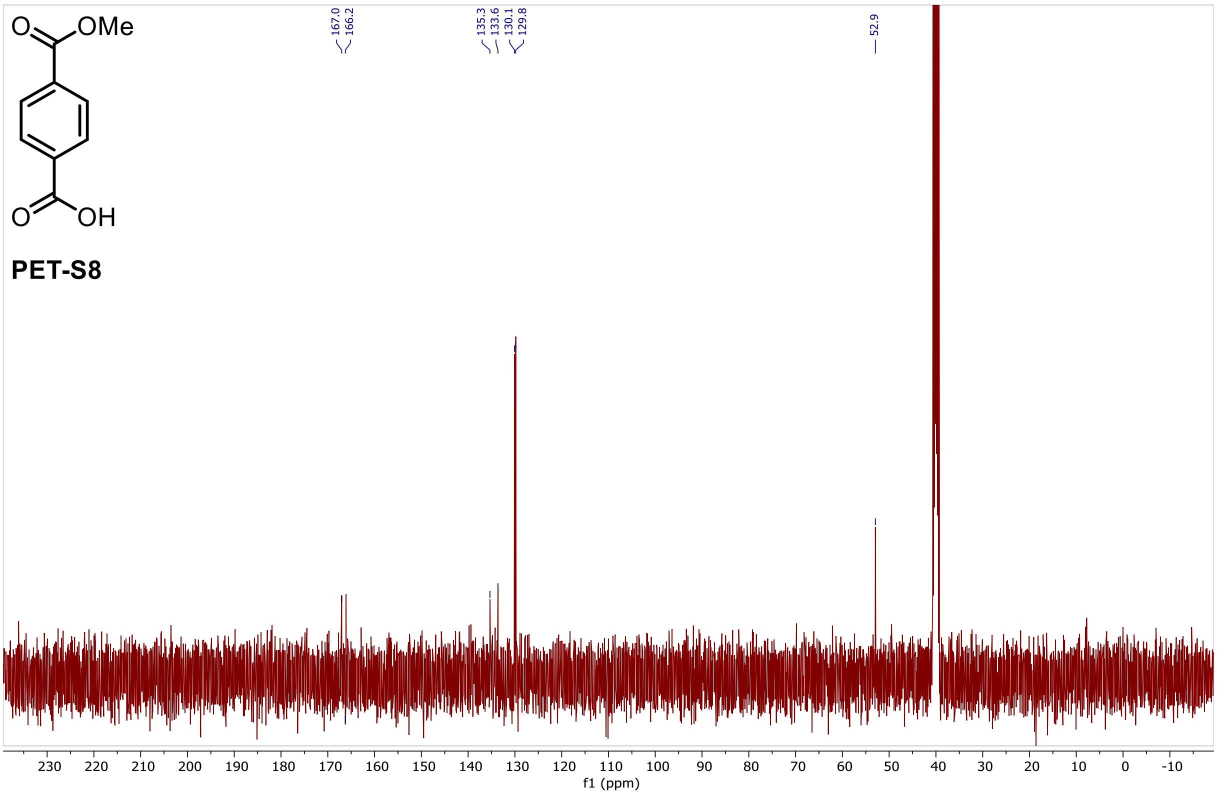

^{1}H NMR spectrum of **PET-1** in DMSO-d_6

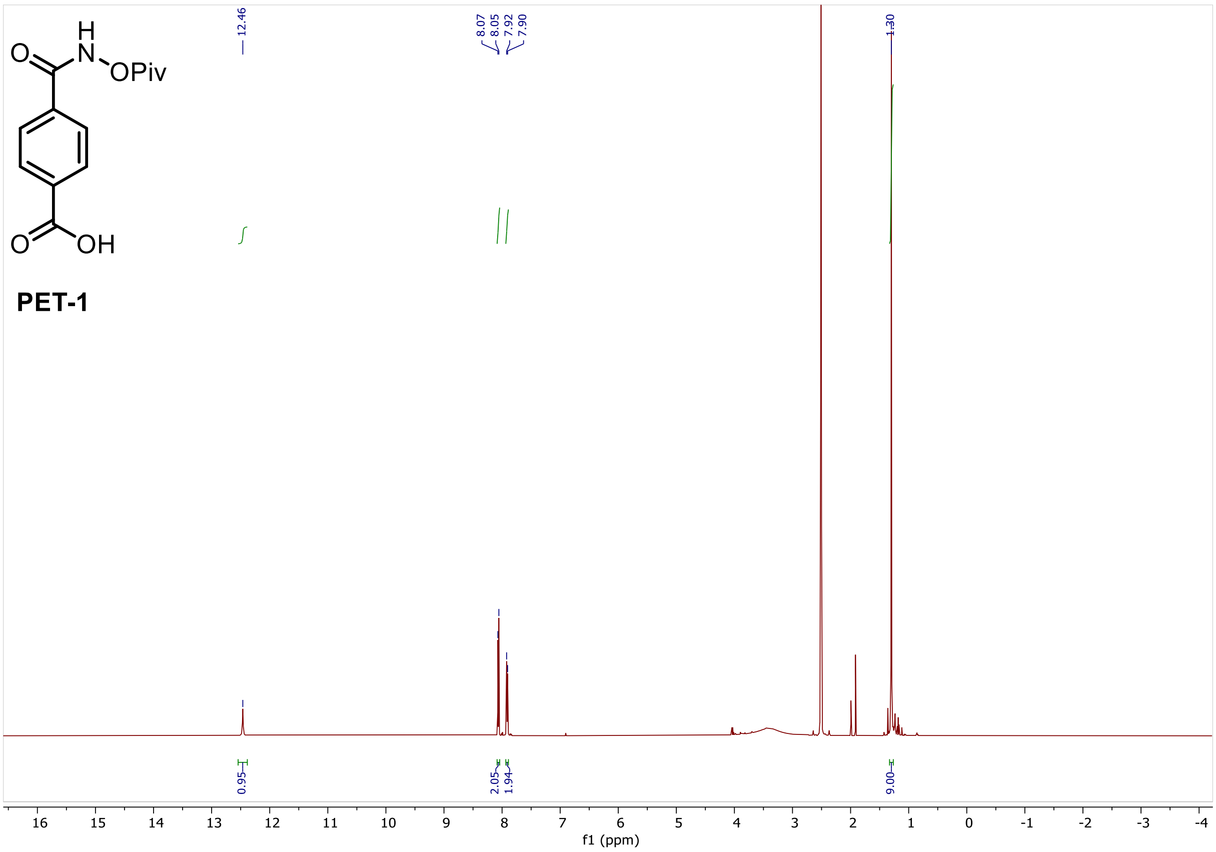

^{13}C NMR spectrum of **PET-1** in DMSO-d_6.

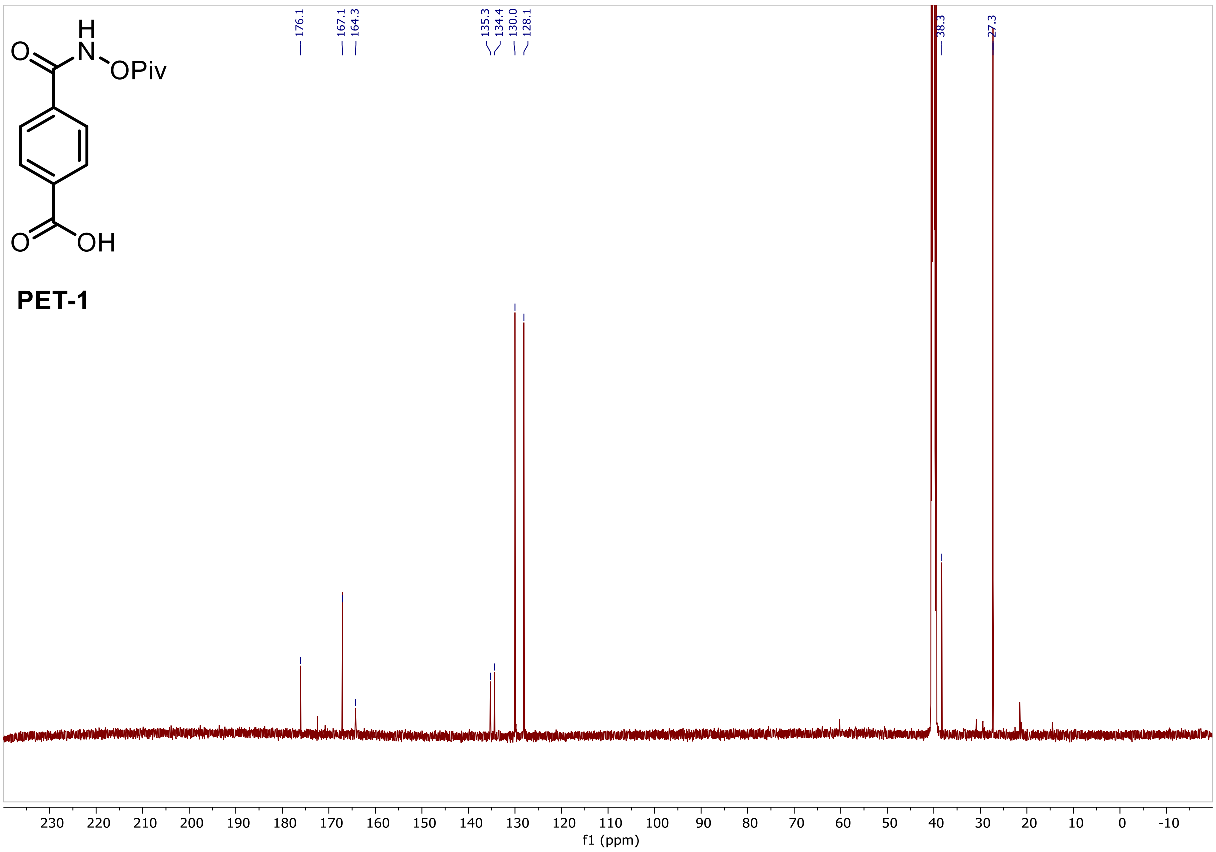

S9. References

1. Germeroth, A. I. *et al.* Triazole biotin: A tight-binding biotinidase-resistant conjugate. *Org Biomol Chem* **11**, 7700–7704 (2013).

2. Cohen, S. N., Chang, A. C. & Hsu, L. Nonchromosomal antibiotic resistance in bacteria: genetic transformation of Escherichia coli by R-factor DNA. *Proc Natl Acad Sci U S A* **69**, 2110–2114 (1972).

3. Valenzuela-Ortega, M. & French, C. Joint universal modular plasmids (JUMP): A flexible vector platform for synthetic biology. *Synth Biol* **6**, ysab003 (2021).

4. Hou, F., Xian, M. & Huang, W. De novo biosynthesis and whole-cell catalytic production of paracetamol on a gram scale in Escherichia coli. *Green Chemistry* **23**, 8280–8289 (2021).

5. Meyer, A. J., Segall-Shapiro, T. H., Glassey, E., Zhang, J. & Voigt, C. A. Escherichia coli "Marionette" strains with 12 highly optimized small-molecule sensors. *Nat Chem Biol* **15**, 196–204 (2019).

6. Presset, M., Oehlrich, D., Rombouts, F. & Molander, G. A. Complementary regioselectivity in Rh(III)-catalyzed insertions of potassium vinyltrifluoroborate via C-H activation: Preparation and use of 4-trifluoroboratotetrahydroisoquinolones. *Org Lett* **15**, 1528–1531 (2013).

7. Pham, D. D. & Cho, J. Low-energy catalytic methanolysis of poly(ethyleneterephthalate). *Green Chemistry* **23**, 511–525 (2021).

8. Li, Z. *et al.* An improved and practical synthesis of tranexamic acid. *Org Process Res Dev* **19**, 444–448 (2015).

9. Ye, Y. *et al.* Construction of Isoquinolone Scaffolds on DNA via Rhodium(III)-Catalyzed C–H Activation. *Org Lett* **26**, 3338–3342 (2024).

10. Brewster, R. C., Suitor, J. T., Bennett, A. W. & Wallace, S. Transition Metal-Free Reduction of Activated Alkenes Using a Living Microorganism. *Angewandte Chemie International Edition* **58**, 12409–12414 (2019).
